# Interventions for preventing back pain among office workers – a systematic review and network meta-analysis

**DOI:** 10.5271/sjweh.4070

**Published:** 2022-12-30

**Authors:** Angelika Eisele-Metzger, Daria S Schoser, Meik D Klein, Kathrin Grummich, Guido Schwarzer, Lukas Schwingshackl, Robin Hermann, Bianca Biallas, Christiane Wilke, Joerg J Meerpohl, Cordula Braun

**Affiliations:** 1 Institute for Evidence in Medicine, Medical Center - University of Freiburg, Faculty of Medicine, University of Freiburg, Freiburg, Germany; 2 Cochrane Germany, Cochrane Germany Foundation, Freiburg, Germany; 3 Institute of Movement Therapy and Movement-oriented Prevention and Rehabilitation, German Sport University Cologne, Cologne, Germany; 4 Institute of Medical Biometry and Statistics, Faculty of Medicine and Medical Center, University of Freiburg, Freiburg, Germany; 5 Institute for Workplace Health Promotion, Cologne, Germany

**Keywords:** computer worker, musculoskeletal pain, neck pain, occupational health, primary prevention, sedentary behavior, workplace

## Abstract

**Objective:**

Back pain is common in the working population. This systematic review with network meta-analysis (NMA) aimed to compare the effects of interventions for preventing back pain among office workers.

**Methods:**

We searched eight databases and additional sources up to March 2021. We included randomized controlled trials (RCT) and cluster RCT focusing on office workers, comparing work-related interventions aimed at preventing back pain (defined as pain in any part of the spine) to a control condition and assessing back pain and/or work absence. Further outcomes considered were adverse events and participants’ satisfaction. We performed both frequentist and component NMA. Risk of bias (RoB) was evaluated using RoB 2 and certainty of the evidence (CoE) was assessed using GRADE.

**Results:**

We screened 9809 records and included 24 studies with a total of 7080 participants. RoB was assessed as “some concerns” or “high” for all studies and outcomes. Included studies investigated multicomponent interventions, ergonomics, physical activity, education, behavioral interventions and no/minimal interventions. Effects were mostly not statistically significant and based on low/very low CoE. Physical activity probably reduces days of work absence slightly [mean difference (MD) -1.10, 95% confidence interval (CI) -2.07– -0.13], and combining physical activity and ergonomics may reduce back pain intensity (standardized MD -0.41, 95% CI -0.80– -0.02) when compared to no/minimal intervention. A large proportion of participants were satisfied with the interventions, adverse events were rarely assessed.

**Conclusions:**

We observed mostly minor effects of interventions on back pain and work absence among office workers. The practical relevance of these effects is questionable.

Low-back and neck pain are leading causes of years lived with disability worldwide (1, 2). The global point prevalence of low-back pain was 7.0% in 2019, with over half a billion persons worldwide suffering from low-back pain (3). Back problems cause high direct healthcare costs and additionally indirect costs due to high numbers of lost work days (4). A sedentary lifestyle is regarded as an important risk factor for back pain (5, 6). Accordingly, work-related interventions to prevent and manage back pain among office workers have become increasingly popular (7–9).

In previous systematic reviews, physical activity (8, 10) and ergonomic interventions (7, 8, 10) have demonstrated certain beneficial effects on back pain among office workers, however, results are inconsistent across different reviews and studies (7–9). For other interventions, such as education or behavioral interventions, no beneficial effects for back pain among office workers have been found so far (8). It has to be noted that interventions examined in previous systematic reviews were often heterogeneous (8, 9) and partly contained intervention components that were not individually evaluated, such as additional behavioral components (8, 10). The available meta-analyses are mostly based on small subsamples of the included studies, partly because of the mentioned heterogeneity (8–10). Over the past years, work-related back pain interventions including multiple components and focusing on various aspects, such as physical, psychosocial and workplace factors, have gained increased attention (11). Such multicomponent interventions have, eg, been included in recent systematic reviews targeting workers (12) or the general population (13). A quantitative summary of results of the included multicomponent interventions and other types of interventions has, however, only been possible to a very limited extent in those systematic reviews (12, 13). To address the complexity and heterogeneity of work-related back pain interventions, we therefore aimed at conducting a comprehensive systematic review with network meta-analysis (NMA). While traditional pairwise meta-analysis is a useful method to compare two interventions, NMA enables comparing and ranking the effects of multiple interventions in a single analysis (14). As a further advantage, direct evidence from available pairwise comparisons of interventions is complemented by indirect evidence derived from the available network of interventions (14). Additionally, effects of single components included in multicomponent interventions can be further investigated using component NMA (CNMA) (15). To the best of our knowledge, interventions for preventing back pain among office workers have not been summarized in a systematic review with NMA or CNMA so far.

Therefore, this systematic review with NMA aimed to investigate the effects of different work-related interventions for preventing back pain among office workers. The specific aims were (i) to determine the effects of different interventions on back pain (defined as pain in any part of the spine) and work absence, (ii) to explore potential intervention-related adverse events and participants’ satisfaction with the interventions, (iii) to combine direct and indirect evidence and rank different intervention strategies based on their effects using NMA, and (iv) to rate the certainty of evidence (CoE) for the investigated outcomes.

## Methods

This systematic review is reported in accordance with the Preferred Reporting Items for Systematic reviews and Meta-Analyses (PRISMA) 2020 statement (16) and the extension for reporting NMA of healthcare interventions (17) (supplementary material, www.sjweh.fi/article/4070, table S1).

### Protocol and registration

This systematic review was conducted according to an a priori developed and published protocol (18). Additionally, the protocol is registered in PROSPERO (www.crd.york.ac.uk/prospero/display_record.php?ID=CRD42021232469). Any deviations from the protocol as well as methods that were planned but could not be conducted, are reported in supplementary table S2.

### Eligibility criteria

Parallel-group and crossover randomized controlled trials (RCT) and cluster RCT fulfilling the following criteria were included:

*Types of participants*. Studies had to focus on people performing office work in an employment-related context. We aimed at including studies primarily investigating people without back pain at baseline. However, given the widespread prevalence and recurrent nature of back pain (19), we also considered studies including proportions of participants with back pain (defined as pain in any part of the back, ie, the neck, upper back and/or lower back, possibly extending to other parts of the body, eg, the shoulder or buttocks). When we developed our review, we anticipated that the majority of studies would include both participants with and without back pain. This was backed by the results of our preliminary searches and was consequently backed by the results of our final searches (only one study included exclusively people without back pain at baseline). We excluded studies specifically designed to investigate back pain treatment effects, ie, studies in which all participants had back pain at baseline, were unable to work, or had a specific predisposition such as reduced spinal mobility or a history of back pain in recent months.

*Types of interventions*. We included studies investigating work-related interventions aimed at preventing back pain and delivered within workplace settings. We have predefined the considered types of interventions in our protocol (18). Based on these predefined categories and in alignment with the actual interventions found, the following intervention categories were chosen (for definitions see supplementary table S3): “Behavioral intervention”, “education”, “ergonomics”, “exercise equipment”, “physical activity”, “multicomponent intervention with physical activity”, “other multicomponent intervention”, and “no/minimal intervention”. We decided to differentiate between multicomponent interventions including and not including physical activity considering previous reviews that demonstrated beneficial effects of physical activity alone or in combination with other interventions on back pain (eg, 13, 20, 21.).

*Types of outcome measures*. The primary outcomes of interest for our review were non-specific back pain (numbers of participants with at least one episode of back pain or intensity of back pain) and work absence (numbers of participants absent from work or numbers of work absence days). Non-specific back pain was defined as pain in any part of the back, ie, the neck, upper back and/or lower back (possibly extending to other parts of the body, eg, the shoulder or buttocks), without an underlying specific pathology such as trauma, inflammatory disease or neoplasm (22). To be included in our review, studies had to contain a follow-up assessment of either back pain or work absence of ≥24 weeks post-baseline. The secondary outcomes of interest were adverse events (numbers of participants who experienced an adverse event) and self-reported satisfaction with the intervention.

### Information sources and search strategy

*Electronic searches*. We searched the following electronic databases from their inception up to 3 March 2021: Cochrane CENTRAL, MEDLINE via PubMed, Web of Science, CINAHL, PsycINFO, PEDro, SPORTDiscus and Academic Search Premier. We used a sensitive search strategy with terms (MeSH terms and relevant keywords) related to the population/health problem of interest (back pain) and intervention setting (workplace) (supplementary table S4). We applied no language restrictions for the search but included only studies reported in English or German. We documented any potentially relevant studies published in other languages (supplementary table S5). Studies for which only a brief summary (eg, conference abstract) was available were excluded.

*Searching other resources*. We searched the following sources for ongoing or unpublished studies on 16 April 2021: International Clinical Trials Registry Platform of the World Health Organization (ICTRP), German Clinical Trials Register (DRKS) and ClinicalTrials.gov. We checked the reference lists of all included studies and of relevant systematic reviews, and we contacted experts in the field for further potentially relevant studies.

### Data collection and analysis

*Selection of studies*. Two sets of two reviewers independently assessed all retrieved records in a two-stage process (screening of titles/abstracts followed by screening of potentially relevant full texts). Any disagreements were discussed and resolved, if necessary, involving a third reviewer.

*Data extraction and management*. Two sets of two reviewers independently extracted the following details for each included study using a purpose-developed, piloted data extraction sheet: First author, publication year, study design, country, study duration, sample sizes (total and per intervention group), participants’ age, gender and highest level of education, work setting and occupations, description of interventions, outcomes (and outcome measures), results for each outcome of interest and each intervention group and information on funding sources and potential conflicts of interest. Any discrepancies between reviewers were resolved through discussion, involving, if needed, a third reviewer. For studies reported in multiple records, we aggregated the available information and defined a primary report which we used as a reference for the results section. See supplementary table S6 for an example of extracted data.

We extracted results for the primary outcomes for all reported time points ≥24 weeks from baseline and for secondary outcomes for all time points reported. In order to consider a timeframe appropriate for our primary preventive research question and at the same time include as many studies as possible in our analyses, we conducted our main analyses using the results assessed closest to 12 months. Further follow-up measurements were considered in additional analyses (see further).

*Assessment of risk of bias in included studies*. We assessed risk of bias (RoB) using the updated Cochrane RoB 2 tool (23). Two sets of two reviewers independently assessed RoB for each included study and primary outcome (discrepancies were resolved as described above). Where measurement methods were comparable for multiple outcomes (eg, questionnaire assessment), we grouped them and assessed them together per study. The RoB 2 tool comprises five domains and an additional domain for cluster RCT (23). RoB is rated for each domain resulting in an overall RoB judgement of either “low RoB”, “some concerns” or “high RoB” (23).

*Measures of treatment effect*. We used risk ratios (RR) with 95% confidence intervals (CI) for dichotomous data and mean differences (MD) with standard deviations (SD) for continuous data. Where an outcome had been obtained using different measurements (eg, different pain scales), we used Hedges’ g as standardized mean difference (SMD). We prioritized mean change scores from baseline to follow-up over mean scores at follow-up, where both were available.

*Unit of analysis issues*. For cluster RCT, we used cluster-adjusted effect estimates reported in the original studies, if study authors applied an appropriate analysis method to account for clustering (24). If cluster-adjusted effect measures were unavailable, we adjusted for clustering following Cochrane guidance (25), ie, we reduced the sample size of the respective cluster RCT to its “effective sample size” considering the “design effect” [which depends on the average cluster size as well as the intracluster correlation coefficient (ICC)]. The same approach was applied to binary outcomes. As none of the included cluster RCT reported calculated ICC, we assumed an ICC of either 0.05 or 0.02 based on the reported assumed ICC in included cluster RCT and carried out the analyses for both scenarios. As differences in the results were small, we present the results of the analyses using the more conservative ICC of 0.05. If the average cluster size for a trial could not be determined, we calculated the effective sample size by assuming the maximum of all design effects across all other included cluster RCT.

*Dealing with missing data*. If data were missing or unclear, we contacted the study authors for further information. In case of missing SD and standard errors (SE), we calculated SD using reported CI following Cochrane guidance (26). If neither SD, SE, CI or P-values were reported in a study, we imputed the median of reported SD from other included studies (27).

*Assessment of risk of publication bias*. We assessed publication bias in networks with at least ten studies by examining comparison-adjusted funnel plots (28) and conducting Egger’s linear regression test for funnel plot asymmetry (29).

*Data synthesis*. We performed three analysis steps. First, we conducted random-effects pairwise meta-analyses for all direct comparisons of interventions with other interventions. For two studies (30, 31) which examined “ergonomics” in more than one study arm, we pooled results for this intervention category for the meta-analyses and the subsequent NMA (32). We present the results of the pairwise comparisons using forest plots (supplementary figure S1a–c). For each pairwise meta-analysis, we examined statistical heterogeneity among studies using Cochran’s Q test and the I^2^ statistic (33). If we found considerable heterogeneity for a direct comparison (ie, I^2^ >75%) (34), we performed leave-one-out meta-analyses and generated Baujat plots (35) to identify potential outliers. Based on this, we excluded one study (36) from the pairwise meta-analyses and further NMA for the outcomes back pain intensity and days of work absence (supplementary table S7a–b and supplementary figure S2a–b).

In a second step, we conducted NMA using a frequentist approach (37) including all available interventions categorized as described above. Summary effect estimates along with their 95% CI are provided in league tables (displaying NMA effects compared with direct effects) (14). We illustrated the network structure for each outcome using network graphs (38). For each NMA, we ranked interventions according to P-scores (37), which can take values between 0 and 1 and indicate the probability of being more effective compared to other interventions. To illustrate the potential impact of RoB, we generated network graphs with colored edges representing the overall RoB of the studies contributing to the respective comparisons (28).

Lastly, we conducted additional CNMA to evaluate the effects of single components in multicomponent interventions (15). For these analyses, we identified the intervention components within our intervention categories “multicomponent intervention with physical activity” and “other multicomponent intervention”.

Most studies reported defined back pain localizations (eg, neck or lower back) which we combined to an overall outcome “back pain” (ie, back pain intensity or participants with back pain) for our main analyses. If studies reported more than one localization, we used only one of them for our analyses, following the hierarchy (i) lower back, (ii) neck (-shoulder), (iii) upper back, (iv) back including various regions. We chose this approach considering global burden of disease rankings (1) and the known high numbers of lost work days due to low-back and neck pain (4).

All analyses were performed with R using the R packages meta (39) and netmeta (40). See supplementary table S8 for an example of the analytical code.

*Additional analyses*. Pre-specified additional analyses were conducted (18). We performed sensitivity analyses excluding studies with high RoB from the NMA to assess the impact of RoB on our results. To account for a possible impact of follow-up duration, we performed additional NMA that separately considered results assessed in the timeframe from 24 weeks to <12 months (medium-term) and after ≥12 months (long-term) using the longest available follow-up per study. We furthermore conducted additional NMA for different back pain localizations (lower-back pain, neck [-shoulder] pain, upper back pain and back pain including various regions) and different intervention durations (≤6 months and >6 months, based on the longest intervention in each individual study). The additional NMA for different follow-ups and intervention durations could only be performed for participants with back pain as there were too few studies with overlap for the same intervention categories for the other outcomes.

*Assessment of transitivity*. To explore the assumption of transitivity (41), we assessed the distribution of possible effect modifiers (age, gender and intervention duration) across the available direct comparisons as well as the characteristics of further variables (delivery of interventions and study settings). We found no serious imbalances and therefore concluded that the assumption of transitivity was not violated.

*Assessment of consistency*. To evaluate consistency, we used the node splitting approach (42) and a design-by-treatment interaction model to test for design inconsistency in the whole network (43). We did not detect substantial inconsistency in our NMA.

*Rating the certainty of the evidence*. Two sets of two reviewers independently rated CoE derived from our NMA following the GRADE (Grading of Recommendations Assessment, Development and Evaluation) approach (44, 45) (disagreements were resolved as described above). Applying GRADE for NMA involves a three-step procedure including rating the certainty of the available direct and indirect estimates as well as network estimates (44–46). The approach results in four possible levels of certainty: High, moderate, low and very low (44). For the reporting of results, we followed the GRADE guidance on communicating findings of systematic reviews (47).

## Results

**Figure 1:**
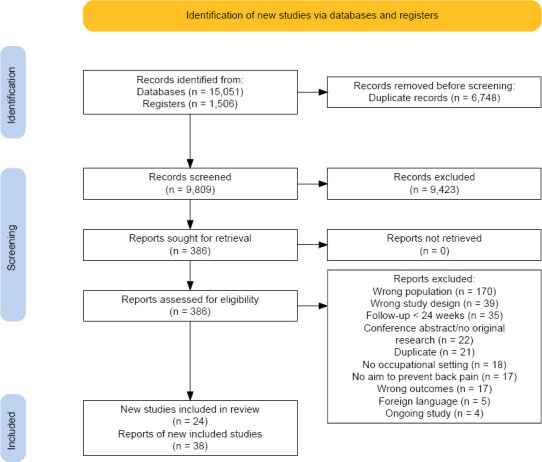
PRISMA flow diagram of the study search and selection process. The diagram was created using software provided by Haddaway et al (87).

Our search resulted in 16 557 records, with 9809 records remaining after removal of duplicates. Screening of titles and abstracts resulted in 386 full texts that were assessed for eligibility. We finally included 24 studies, reported in 38 articles (30, 31, 36, 48–82), in our qualitative synthesis and 18 studies, reported in 29 articles (30, 31, 36, 48–51, 55–59, 62, 63, 65, 66, 68–75, 78–82), in our meta-analyses. We identified three potentially relevant ongoing studies, reported in four articles (83–86). The study selection process including the main reasons for the exclusion of studies after full text screening is shown in a PRISMA flow diagram (16, 87) (figure 1). We contacted 17 authors (36, 49, 50, 52, 54–57, 59, 60, 64–66, 70, 74, 77, 80) to request additional data, of which 9 (36, 50, 55–57, 66, 74, 77, 80) responded and 3 (55, 66, 74) were able to provide the requested information.

### Study characteristics

An overview of the study characteristics is given in table 1. Of the 24 studies included, 13 were cluster RCT (49, 50, 54–56, 59, 60, 68, 70, 71, 74, 77, 80) and 11 parallel-group RCT (30, 31, 36, 52, 57, 64–66, 72, 75, 76). Of the studies, 18 had two intervention arms, 3 had three (31, 49, 52) and another 3 had four (30, 60, 76). The studies reported on a total of 7080 participants. The study duration (from enrolment of participants to the last follow-up) ranged from approximately 9 to 40 months but was not clearly reported in many cases. Maximum follow-up from baseline ranged from 6 to 24 months. The majority of included studies were conducted in Europe or the USA.

**Table 1:** Characteristics of included studies. [SD=standard deviation; RCT=randomised controlled trial; NR=not reported].

Study, country	Study design (total N randomised)	Mean age (SD); N female gender (%)	Highest level of education [N (%)]	Setting / occupations	Intervention classification	Name of interventions (N randomised)	Longest follow-up (months)	Outcomes
Andersen 2008, Denmark	Cluster RCT (549)	45.0 (9.4^a^; 397 (64.4%)	NR	Office workers (public administration authority)	Physical activity	Specific resistance training (180)	12	Participants with neck (-shoulder) pain^b^, neck (-shoulder) pain intensity^b^, adverse events
					Multicomponent intervention including physical activity	All-round physical exercise (187)		
					Education	Reference intervention (182)		
Baydur2016,Turkey	Cluster RCT (116)	36 (8.4); 69 (59.5%)	Elementary school: 1 (0.9%), Junior high school: 2 (1.7%), Senior high school: 37 (31.9%), University: 76 (65.5%)	Office workers (municipality)	Other multicomponent intervention	Participatory ergonomic intervention (58)	13	Participants with neck (-shoulder) pain ^b^
					No/minimal intervention	Control group (58)		
Bohr2000,USA	RCT (154)	42.5 (SD NR); 133 (86.4%)	NR	Agents (centralised reservation facility for an international transportation company)	Education	Traditional education (51)	12	Participants with pain in various regions
					Ergonomics	Participatory education (50)		
					No/minimal intervention	Control group (53)		
Brakenridge2018,Australia	Cluster RCT (153)	38.9 (8.0); 70 (45.8%)	≥University: 121 (84%)	Office workers (international property and infrastructure group)	Other multicomponent intervention	Organisational support (87)	12	Participants with neck (-shoulder) pain, participants with lower back pain, participants with upper back pain, neck (-shoulder) pain intensity, lower back pain intensity, upper back pain intensity, intervention satisfaction
					Other multicomponent intervention	Organisational support + tracker (66)		
Brisson1999,Canada	Cluster RCT (774)	43 (SD NR); N female gender NR (80%)	NR	Workers employed in a large university and in other institutions involved in university services	Ergonomics	Ergonomic training program (N randomised NR)	6	Participants with neck (-shoulder) pain^b^, participants with lower back pain ^b^
					No/minimal intervention	Reference group (N randomised NR)		
Coenen2017,Australia	Cluster RCT (231)	45.6 (9.4); 158 (68.4%)	Post school education: 151 (66.8%)	Office workers at a government department (managers/administrators, professionals/associates, clericals/sales/service)	Other multicomponent intervention	Stand up Victoria (136)	12	Days of work absence^b^, intervention satisfaction, adverse events
					No/minimal intervention	Control group (95)		
Conlon 2008,USA	RCT (206)	42.9 (9.8) ^a^; 57 (27.8%) ^a^	High school: 17 (8.5%) ^a^, College: 70 (33.8%) ^a^, Graduate school: 121 (58.5%) ^a^	Engineers and professionals that support engineering projects (e.g. programmers, graphic designers, project developers) at an aerospace engineering firm	No/minimal intervention	Conventional mouse (52)	12	Participants with neck (-shoulder) pain ^b^, neck (-shoulder) pain intensity
					Ergonomics	Alternative mouse (52)		
					Ergonomics	Conventional mouse + forearm support board (51)		
					Ergonomics	Alternative mouse plus forearm support board (51)		
Dalager 2017,Denmark	RCT (387)	44 (10); 286 (74%)	NR	Office workers from two private companies, two public municipalities, and two national boards	Physical activity	Training group (193)	12	Neck (-shoulder) pain intensityb, lower back pain intensityb, upper back pain intensityb, days of work absence ^b^
					No/minimal intervention	Control group (194)		
Edwardson 2018, UK	Cluster RCT (146)	41.2 (11.1); 116 (79%)	NR	Hospital employees conducting sedentary work at the office	Other multicomponent intervention	Stand More At Work Intervention (77)	12	Participants with neck (-shoulder) painb, participants with lower back pain ^b^, days of work absenceb, intervention satisfaction
					No/minimal intervention	Control group (69)	
Eklöf 2006, Sweden	Cluster RCT (396)	NR	NR	White-collar workers (banking, transport, manufacturing industry, software engineering, public administration and wholesale)	Other multicomponent intervention	Individual feedback (97)	6	Participants with pain in various regions
					Other multicomponent intervention	Supervisor feedback (106)		
					Other multicomponent intervention	Group feedback (98)		
					No/minimal intervention	Control group (95)		
Gerr 2005, USA	RCT (356)	Age NR; 279 (77.1%) ^a^	college graduates: 258 (71.3%) ^a^	Newly hired persons working with a computer workstation in office settings	Ergonomics	Alternate intervention (121)	6	Participants with neck (-shoulder) pain ^b^
					Ergonomics	Conventional intervention (122)		
					No/minimal intervention	No Intervention (113)		
					Other multicomponent intervention	Usual practice (123)		
Joines 2015, USA	RCT (100)	Age NR; 85 (89.4%)	High school: 9 (9.5%), Junior College (1-2 yrs.): 28 (29.5%), College graduates: 42 (44.2%), Graduate school: 16 (16.8%)	Clinical researchers at a university/medical centre	Ergonomics	Intervention group (N randomised NR)	6	Neck (-shoulder) pain intensity, lower back pain intensity, upper back pain intensity, intervention satisfaction
					No/minimal intervention	Control group (N randomised NR)		
Karatrantou2020,Greece	RCT (40)	43.4 (5.9); 21 (58.3%)	NR	Administrative office staff	Physical activity	Training group (20)	6	Participants with neck (-shoulder) pain ^b^, participants with lower back pain ^b^, participants with upper back pain ^b^, pain intensity - various regions ^b^, days of work absence ^b^, intervention satisfaction, adverse events
					No/minimal intervention	Control group (20)		
King2013,Canada	RCT (23)	NR	NR	Office workers (research organisation)	Behavioural intervention	Biofeedback mouse (11)	6	Pain intensity - various regions^b^, intervention satisfaction
					No/minimal intervention	Control group (12)		
Konradt2020,Germany	RCT (127)	Age NR; 68 (58.6%)	NR	University employees (scientific, services)	Other multicomponent intervention	Sit-stand office desks (58)	24	Participants with neck (-shoulder) pain ^b^, participants with lower back pain ^b^, neck (-shoulder) pain intensity ^b^, lower back pain intensity ^b^
					No/minimal intervention	Control group (69)		
Lee2020,Brazil	Cluster RCT (64)	29.0 (6.1) ^a^; 41 (64.1%)^a^	Undergraduate: 16 (25%), Graduate: 48 (75%)	Office workers (distance education sector of a university)	Ergonomics	Ergonomic workstation intervention (32)	9	Neck (-shoulder) pain intensity ^b^, lower back pain intensity ^b^, upper back pain intensity ^b^
					No/minimal intervention	Control group (32)		
Mahmud2010,Malaysia	Cluster RCT (179)	34.4 (9.4) ^a^; 134 (74.9%) ^a^	High school: 78 (43.5%) ^a^, Technical certificate/diploma: 50 (28.0%) ^a^, Degree (bachelor’s/post-degree): 36 (20.2%) a, Other: 15 (8.3%) ^a^	Office workers (bursary, registry, library, research management centre, professional and continuing education, centre of information and communication technology)	Other multicomponent intervention	Office ergonomics training (89)	12	Participants with neck (-shoulder) pain ^b^, participants with lower back pain ^b^, participants with upper back pain ^b^, days of work absence ^b^
					No/minimal intervention	Control group (90)		
Meijer2009,Netherlands	Cluster RCT (354)	44 (SD NR); 166 (46.9%)	NR	Office workers (regional governmental institute)	Behavioural intervention	Computer mouse with feedback signal (178)	8	Participants with pain in various regions ^b^
					No/minimal intervention	Control group (176)		
Moore2012,USA	RCT (54)	49.3 (SD NR); 23 (76.7%)^a^	NR	University employees (clerical, educational, administration, nursing, laboratory assistant) with sedentary job	Physical activity	Daily exercise (N randomised NR)	12	Participants with lower back pain ^b^
					No/minimal intervention	Normal activity (N randomised NR)		
Pereira2019,Australia	Cluster RCT (763)	42.7 (10.7^a^; 452 (59%)	Primary to year 12: 85 (23.2%) ^a^, University: 230 (62.7%) ^a^, Trade college: 52 (14.2%) ^a^	Office workers (public service, administration, manufacturing/construction, higher education, insurance and local government)	Multicomponent intervention including physical activity	Ergonomics and exercise training (381)	12	Neck (-shoulder) pain intensity ^b^, days of work absence ^b^, intervention satisfaction, adverse events
					Other multicomponent intervention	Ergonomics and health promotion (382)		
Proper2003,Netherlands	RCT (299)	43.9 (8.9) ^a^; 99 (32.8%)^a^	Highly educated: 196 (65.5%) ^a^	Civil servants of three municipal services	Other multicomponent intervention	Individual counselling (131)	9	Participants with lower back pain ^b^
					No/minimal intervention	Control group (168)		
Rempel2006,USA	RCT (182)	40.0 (12.1)^a^; 175 (95.3%) ^a^	High school: 47 (25.7%) ^a^, Some college: 77 (42.5%) ^a^, Completed college: 59 (32.3%) ^a^	Registered nurses or healthcare specialists working at two customer service centre sites of a large healthcare company	Ergonomics	Ergonomic training (46)	12	Participants with neck (-shoulder) pain, neck (-shoulder) pain intensity, intervention satisfaction
					Ergonomics	Ergonomic training + trackball (45)		
					Ergonomics	Ergonomic training + armboard (46)		
					Ergonomics	Ergonomic training + trackball + armboard (45)		
Renaud 2020,Netherlands	Cluster RCT (244)	42.3 (10.2) ^a^; 146 (59.8%) ^a^	No/primary education: 7 (2.9%) ^a^, Secondary education: 78 (32.0%) ^a^, Professional education: 65 (26.6%) ^a^, University education: 94 (38.5%) ^a^	Office workers (insurance company)	Other multicomponent intervention	Dynamic work intervention (121)	8	Participants with neck (-shoulder) pain, participants with lower back pain, pain intensity - various regions, participants absent from work
Speklé2010,Netherlands	Cluster RCT (1,183)	44.1 (9.4) ^a^; 471 (39.8%) ^a^	NR	Office workers (office staff, local government officials, engineers, consultants, teachers, health care personnel, nature conservation professionals, researchers, managers)	Multicomponent intervention including physical activity	Intervention group (605)	12	Participants with neck (-shoulder) pain ^b^, days of work absence ^b^
					No/minimal intervention	Usual care (578)		

a Not reported for total sample and calculated from reported groups.

b Included in quantitative data synthesis (NMA).

*Participant characteristics and setting*. Participants were office workers, employed in different areas, such as administration, engineering or healthcare. Participants’ highest level of education was not reported in many studies, but in those in which it was reported, often a substantial proportion of participants had higher education (eg, university degrees; see table 1). All but one (72) study included participants with back pain or some other musculoskeletal complaints at baseline in addition to pain-free participants. Mean age of populations ranged from 29 to 49 years. All studies included male and female participants, with a higher proportion of females in most studies.

*Intervention characteristics*. Following the classification mentioned before (supplementary table S3), studies included the following intervention categories: “behavioral intervention” (N=2), “education” (N=2), “ergonomics” (N=13), “physical activity” (N=4), “multicomponent intervention with physical activity” (N=3), “other multicomponent intervention” (N=14) and “no/minimal intervention” (N=19). “Exercise equipment” was not included in a study as a stand-alone intervention but as an intervention component in one “multicomponent intervention with physical activity” and two “other multicomponent interventions”. Interventions varied in their modes of delivery, eg, they were group-based (eg, training sessions or lectures), delivered on an individual basis (eg, counselling), included environmental changes (eg, adjusting the workplace or adding equipment to the workplace) or included self-directed information material (eg, leaflets or online-videos). Durations of interventions ranged from zero (for no intervention) up to a maximum of 24 months. See supplementary table S9 for a more detailed description of all interventions with their assignment to the intervention categories.

*Funding and conflicts of interest*. Most of the included studies (N=14) were not commercially funded, 3 were (at least partially) commercially funded (57, 66, 77) and for 7, funding was unclear (30, 50, 52, 54, 64, 72, 75). In 11 studies, authors declared that they had no potential conflicts of interest (30, 31, 36, 54, 56, 57, 70, 71, 74, 76, 77), in 2 studies, potential conflicts were reported (59, 80) and 11 studies did not disclose conflicts of interest.

### Risk of bias in the included studies

**Figure 2a–b:**
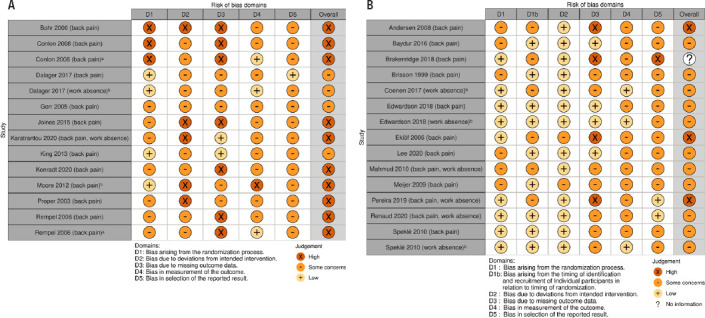
Risk of bias assessment for the included (a) randomised controlled trials (RCT) and (b) cluster RCT at outcome level (outcomes given in parentheses). Outcomes were assessed using questionnaires, if not stated otherwise. ^a^ Assessed by physician; ^b^ Assessed using organisational records; ^c^ Assessed using interviews and health diaries. Brakenridge 2018: We decided not to assess the overall RoB as no results were reported for the follow-up of interest. The figure has been created using the robvis tool provided by McGuinness et al (101).

The results of our RoB assessment for the included studies considering the primary outcomes are displayed in figure 2a–b. If RoB varied for different outcomes (eg, back pain assessed by questionnaire and work absence assessed using organizational records), we present both assessments. Overall RoB was judged as either some or high concerns for all studies. Main domains of concern in many studies were missing outcome data (high loss to follow-up) and lack of blinding (of participants, providers and outcome assessors). Furthermore, the majority of studies did not refer to a pre-specified analysis plan (accessible study protocol). The overall RoB for the comparisons contributing to the different NMA is illustrated in network graphs with colored edges (supplementary figures S3a–c).

### Results of the data synthesis

**Figure 3a–c:**
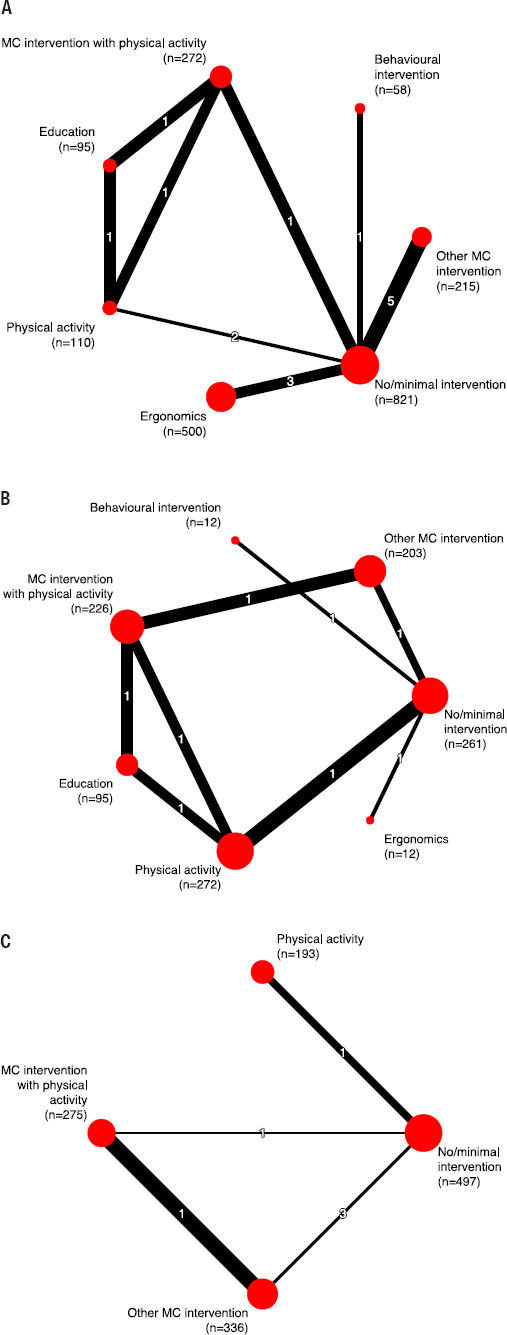
Network graphs ^a^ for the NMAs of the outcomes (a) participants with back pain, (b) back pain intensity, (c) days of work absence. ^a^ The size of each node is proportional to the total number of participants assigned to the respective intervention, the width of each line is proportional to the inverse of the standard error of the respective direct comparison, the numbers on the lines correspond to the numbers of studies contributing to the respective direct comparison. [MC=multicomponent.]

We were able to include 18 studies in our quantitative data synthesis (pairwise meta-analyses and NMA/CNMA). An overview of the NMA results for our primary outcomes is provided in table 2, and the structure of the networks is displayed in figure 3a–c. An overview of the GRADE evaluation of CoE for the NMA of the primary outcomes is provided in supplementary table S10a–c. CoE was rated as low or very low for most comparisons, with few exceptions for the outcome days of work absence.

**Table 2:** League tables with network and direct estimates ^a^ (95% confidence intervals) for the network meta-analysis of the primary outcomes. [CI=confidence interval; RR=risk ratio (RR<1 is beneficial); SMD=standardised mean difference (negative values are beneficial); MD=mean difference (negative values are beneficial); MCI=multicomponent intervention; int.=intervention]

Outcome: Participants with back pain (RR)						
**MCI with physical activity**	1.09 (0.73-1.65)	-	-	-	0.98 (0.67–1.45)	0.82 (0.55–1.22)
1.17 (0.80–1.72)	**Physical activity**	-	-	-	0.90 (0.60–1.36)	0.43 (0.16–1.16)
0.84 (0.53–1.32)	0.71 (0.41–1.25)	**Other MCI**	-	-	-	0.91 (0.70–1.18)
0.68 (0.40–1.17)	0.58 (0.31–1.09)	0.82 (0.52–1.30)	**Ergonomics**	-	-	1.11 (0.76–1.63)
0.71 (0.30–1.65)	0.60 (0.24–1.49)	0.84 (0.38–1.89)	1.03 (0.44–2.42)	**Behavioural int.**	-	1.08 (0.50–2.31)
1.01 (0.69–1.49)	0.86 (0.58–1.30)	1.21 (0.68–2.17)	1.48 (0.78–2.82)	1.44 (0.57–3.61)	**Education**	-
0.76 (0.52–1.11)	0.65 (0.40–1.07)	0.91 (0.70–1.18)	1.11 (0.76–1.63)	1.08 (0.50–2.31)	0.75 (0.45–1.26)	**No/minimal int.**
**Outcome: Back pain intensity (SMD)**						
**MCI with physical activity**	-0.05 (-0.34–0.25)	-0.10 (-0.33–0.14)	-	-	-0.12 (-0.40–0.16)	-
-0.10 (-0.36–0.16)	**Physical activity**	-	-	-	-0.07 (-0.37–0.22)	-0.11 (-0.31–0.09)
-0.06 (-0.28–0.15)	0.04 (-0.26–0.33)	**Other MCI**	-	-	-	-0.26 (-0.67–0.15)
0.25 (-0.58–1.08)	0.35 (-0.46–1.15)	0.31 (-0.52–1.15)	**Ergonomics**	-	-	-0.48 (-1.26–0.30)
-0.08 (-0.93–0.77)	0.02 (-0.80–0.85)	-0.01 (-0.87–0.84)	-0.33 (-1.45–0.79)	**Behavioural int.**	-	-0.15 (-0.95–0.65)
-0.15 (-0.42–0.13)	-0.05 (-0.33–0.24)	-0.08 (-0.41–0.25)	-0.39 (-1.24–0.45)	-0.07 (-0.93–0.80)	**Education**	-
-0.23 (-0.52–0.05)	-0.13 (-0.32–0.06)	-0.17 (-0.46–0.13)	-0.48 (-1.26–0.30)	-0.15 (-0.95–0.65)	-0.09 (-0.41–0.24)	**No/minimal int.**
**Outcome: Days of work absence (MD)**						
**MCI with physical activity**	-	-0.10 (-0.57-0.37)	-2.12 (-7.42–3.18)			
1.19 (-1.20–3.58)	**Physical activity**	-	-1.10 (-2.07– -0.13) ^b^			
-0.12 (-0.59–0.35)	-1.30 (-3.66–1.05)	**Other MCI**	0.64 (-1.71–2.99)			
0.09 (-2.10–2.27)	-1.10 (-2.07– -0.13) ^b^	0.20 (-1.95–2.35)	**No/minimal int.**			

a Network estimates are shown in the lower triangles (direction of comparisons: interventions in the columns versus interventions in the rows), estimates of the available direct pairwise comparisons in the upper triangles (direction of comparisons: interventions in the rows versus interventions in the columns).

b P<0.05.

*Participants with back pain*. For this outcome, we performed a NMA including 13 studies (15 pairwise comparisons, 2070 participants) (supplementary figure S4a–b). Compared to “no/minimal intervention”, the included active interventions [“multicomponent intervention with physical activity” (RR 0.76, 95% CI 0.52–1.11; low CoE), “physical activity” (RR=0.65, 95% CI 0.40–1.07; low CoE), “other multicomponent intervention” (RR 0.91, 95% CI 0.70–1.18; low CoE), “ergonomics” (RR 1.11, 95% CI 0.76–1.63; low CoE), “behavioral intervention” (RR 1.08, 95% CI 0.50–2.31; very low CoE), “education” (RR 0.75, 95% CI 0.45–1.26; low CoE)] may not reduce the number of participants with back pain. For the other comparisons, results were comparable (supplementary table S10a). Ranking interventions using P-scores indicated that “physical activity” (P-score=0.87) might provide the greatest benefit compared to the other interventions, however, this needs to be interpreted with caution given low CoE and no statistically significant results (supplementary table S11). Results were similar for the additional CNMA (supplementary table S12 and supplementary figure S5). For six studies, results can be presented only qualitatively (supplementary table S13) as data were not reported in sufficient detail or in a format we could use for quantitative analysis (52, 54, 60) or the compared interventions were assigned to identical intervention categories (76, 77). Technically, the study by Renaud et al (77) could have been included in the CNMA (as intervention components differed between the interventions), however, this would have resulted in a disconnected network, so we refrained from doing so.

*Back pain intensity*. We performed a NMA including six studies (eight pairwise comparisons, 1081 participants) for this outcome (supplementary figure S6a–b). The investigated active interventions [“multicomponent intervention with physical activity” (SMD -0.23, 95% CI -0.52–0.05; low CoE), “physical activity” (SMD -0.13, 95% CI -0.32–0.06; low CoE), “other multicomponent intervention” (SMD -0.17, 95% CI -0.46–0.13; low CoE), “ergonomics” (SMD -0.48, 95% CI -1.26–0.30; very low CoE), “behavioral intervention” (SMD -0.15, 95% CI -0.95–0.65; very low CoE), “education” (SMD -0.09, 95% CI -0.41–0.24; low CoE)] may not reduce back pain intensity, compared to “no/minimal intervention”. Results were similar for the other comparisons (supplementary table S10b). The highest P-score (0.79) was obtained for “ergonomics” (supplementary table S14). In the additional CNMA, there was no statistically significant effect, except for a reduction in back pain intensity for the combination of “ergonomics” and “physical activity” (SMD -0.41, 95% CI: -0.80– -0.02) compared to “no/minimal intervention” (supplementary table S15 and figure S7). Results of five studies can be presented only qualitatively (supplementary table S13) as data were not reported in sufficient detail or in a format we could use for quantitative analysis (30, 54, 64, 77) or the compared interventions were assigned to identical intervention categories (76).

*Participants absent from work*. This outcome was assessed in only one study which compared two different “other multicomponent interventions” (77). No statistically significant differences were found between intervention groups (supplementary table S13).

*Days of work absence*. For this outcome, we performed a NMA including six studies (six pairwise comparisons, 1301 participants) (supplementary figure S8a–b). “Physical activity” probably reduces the number of days of work absence slightly compared to “no/minimal intervention” (MD -1.10, 95% CI -2.07– -0.13; moderate CoE). The evidence is very uncertain about the effect of “multicomponent intervention with physical activity” (MD 0.09, 95% CI -2.10–2.27; very low CoE) and “other multicomponent intervention” (MD 0.20, 95% CI -1.95–2.35; very low CoE) on days of work absence, compared to “no/minimal intervention”. Compared to each other, “multicomponent intervention with physical activity”, “physical activity” and “other multicomponent intervention” may not reduce days of work absence (low/moderate CoE; supplementary table 10c). “Physical activity” obtained the highest P-score (0.89) (supplementary table S16). The additional CNMA revealed no statistically significant effects, except for a reduction in days of work absence for “physical activity” compared with “no/minimal intervention” (MD -1.17, 95% CI -2.13– -0.21), supporting the results of the NMA (supplementary table S17 and figure S9).

*Intervention satisfaction*. Intervention satisfaction (or related constructs such as acceptability or enjoyment) was assessed in eight studies (36, 54, 56, 59, 64, 65, 74, 76) using questionnaires or qualitative methods such as interviews or focus groups. Due to heterogeneity of assessment methods, no quantitative synthesis could be conducted for this outcome. Results indicated that a large proportion of participants was satisfied with the interventions. There were few reports of problems that may be associated with lower satisfaction, eg, an activity tracker was perceived as not comfortable by one third of participants in Brakenridge et al (54, 88). Further results are summarized in supplementary table S18.

*Adverse events*. Adverse events were heterogeneously monitored in only four studies, which precluded a quantitative synthesis. Two studies included only a brief statement that adverse events were minor and transient (49), or that no adverse events occurred (36). Coenen et al (56) reported 31 adverse events in 29 participants (affecting, eg, upper body, back or lower limbs, and including, eg, headache, eye strain or tiredness) in their intervention group (N=136; “other multicomponent intervention”). Pereira et al (74) reported two adverse events in one group (N=381; “multicomponent intervention with physical activity”) and zero in the other group (N=382; “other multicomponent intervention”).

*Additional analyses*. Excluding studies with high overall RoB resulted in little to no change in NMA results (supplementary figure S10a–c and table S19a–c). Only for the outcome participants with back pain, “physical activity” (which formerly had the highest P-score) dropped out due to high RoB of the respective studies, resulting in some change. The results of the additional NMA for different back pain localizations (supplementary figure S11a–g and table S20a-g) were similar to those of the main analyses. However, some of the additional NMA for different localizations were based on very few studies, resulting in minor changes. The sensitivity analyses for medium- and long-term follow-up (supplementary figure S12a–b and table S21a–b) as well as different intervention durations (supplementary figure 13a–b and table S22a–b) for the outcome participants with back pain also confirmed the results of our main analysis.

### Risk of publication bias

Testing for funnel plot asymmetry is recommended only for syntheses of ≥10 studies (89). Therefore, we were able to assess publication bias only for the outcome participants with back pain. Examination of the comparison-adjusted funnel plot and Egger’s linear regression test (P=0.1582) revealed no evidence of funnel plot asymmetry/publication bias (supplementary figure S14).

## Discussion

### Summary of main results

Our systematic review included 24 studies and synthesized the available data for different interventions aimed at preventing back pain among office workers on pain- and work-related outcomes using NMA and CNMA methodology. Furthermore, we assessed participants’ satisfaction with the interventions and adverse events, where reported by the included studies. Overall, we found only minor effects on back pain and work absence that might be considered not practically important.

RoB of all included studies was judged as either “some concerns” or “high risk”, and CoE was judged as low to very low for most comparisons. This means that our findings need to be interpreted with caution as the effect estimates are partly very uncertain and future research is very likely to have an important impact on our confidence in the effect estimates and is likely to change them.

The included studies investigated multicomponent interventions, ergonomics, physical activity, education, behavioral interventions and no/minimal interventions. We found that physical activity probably reduces days of work absence slightly when compared to no/minimal intervention, however the effect was small and based on only one study (57). Additionally, we found that the combination of physical activity and ergonomics may result in a reduction of back-pain intensity when compared to no/minimal intervention. No statistically significant differences were found for any other comparison and outcome. Ranking interventions using P-scores indicated that physical activity may be considered more effective for the outcomes days of work absence and participants with back pain when compared to the other interventions, but the effects may be regarded as very small. For the outcome back-pain intensity, the highest P-score was obtained for ergonomics. The ranking must be interpreted with caution given the predominantly low CoE and absence of statistically significant effects. Participants’ satisfaction with the interventions was diversely assessed in the included studies, with mainly positive results. Adverse events were only rarely investigated and mostly poorly described.

### Comparison with other systematic reviews

This is the first systematic review with NMA and CNMA on interventions for preventing back pain among office workers. As the included individual studies investigated a range of different interventions, in particular many multicomponent interventions including various combinations of components, the analyses we performed can give more insights than traditional pairwise meta-analysis. Our results are broadly in line with previously published systematic reviews that found some, though inconsistent, effects for physical activity (8, 10) and ergonomics (7–10) for preventing back pain among office workers. Several other systematic reviews on interventions for preventing low-back pain in the general population have found more consistent effects in favor of physical activity or physical activity combined with education (13, 20, 90, 91), probably indicating that these interventions show clearer effects in other populations or specifically in low-back pain. In our additional analyses focusing on lower-back pain, however, we found no considerable differences from our main analyses, contradicting the argument that effects might be more evident in preventing lower-back pain. There are a range of other systematic reviews that examined physical activity as secondary prevention of back pain, i.e. as a treatment for patients with back pain: eg, a recent Cochrane review with 249 included studies showed small beneficial effects of physical activity in treating chronic low-back pain (92). This result is supported by further systematic reviews on the effects of physical activity in chronic low back and neck pain treatment (eg, 93, 94.). Comparing these results to the results of our systematic review, it seems conceivable that the primary preventive effects of physical activity on back pain may be smaller and more difficult to achieve and identify within trials than treatment effects. Furthermore, effects may vary for different populations, eg, patients with persistent back pain, general population with and without (subclinical) back pain and workers in different work settings.

### Strengths and limitations of the review and evidence

We conducted this systematic review in line with established quality standards: (i) the review was based on a prospectively registered and published protocol; (ii) we used rigorous methods following, as far as possible, Cochrane methods; (iii) an experienced information specialist (KG) co-developed and conducted our comprehensive searches, which comprised a broad range of electronic databases and additional sources; (iv) study selection, data extraction and RoB/GRADE assessments were done independently in duplicate, with a third reviewer being involved if needed; and (v) an experienced statistician with specific expertise in meta-analysis and NMA methods (GS) co-developed and conducted our comprehensive and rigorous analysis methods for synthesizing the results of the included studies.

The limitations of our review primarily relate to the low to very low CoE from the included studies. Results may be influenced by the moderate to high RoB that was identified for all original studies as well as the imprecision of nearly all of the obtained effect estimates. High loss to follow-up was one main point of concern in many studies. The resulting small sample sizes may have contributed to the high extent of imprecision for many comparisons. A further weakness of all included studies was lack of blinding of participants and intervention providers, resulting in certain RoB. Poor reporting in some of the original studies [eg, concerning outcome data and assessment methods, intervention characteristics and definitions of study types (eg, clustering)] led to a certain vagueness in our description of study characteristics as well as exclusions from our quantitative synthesis. Contacting authors was, unfortunately, often not successful in receiving the needed data.

We set out to determine the effects of work-based interventions for preventing back pain among office workers. Ideally, this would have resulted in a synthesis of studies on populations including exclusively people without back pain at baseline. However, all but one of the identified studies examined mixed samples of people with and without pain at baseline. This means that the results cannot unambiguously be attributed to people without back pain, ie, to primary prevention. While we consider that this may be viewed as a limitation of our review, we think that it tends to reflect “everyday life” and that it is therefore justified.

We had to limit our review to a selection of primary and secondary outcomes of interest. We acknowledge that further outcomes may be of interest for answering the review question, including, eg, the costs of the interventions. The included studies employed a variety of outcome measurements, which affected the comparability for the quantitative synthesis of results and may have caused certain heterogeneity. For example, studies used different reference periods to assess pain intensity, such as intensity in the preceding seven days or the past month. However, statistical heterogeneity was acceptable in all of our analyses. Although NMA allow the combination of direct and indirect evidence from the original studies, many of our results are based on only few studies and/or only indirect evidence (as can be seen in the network graphs). Lastly, for our NMA and CNMA, we had to classify interventions according to their content which may have been influenced by incomplete reporting of interventions in the original studies as well as subjective opinions of the reviewers. On the other hand, several reviewers conducted and agreed on classification which was built on previous reviews.

### Research implications

We were able to include a solid number of original studies in our systematic review. However, none of the included studies was judged to be at low RoB, and CoE in our results was predominantly low to very low. Therefore, future studies should particularly address methodological shortcomings. Froud et al (95) have pointed out that many trials on back pain interventions are not adequately powered. Future studies should thus include sample sizes large enough to detect also small-to-medium effect sizes (95). Furthermore, study investigators should try to minimize the extent of loss to follow-up and report transparently on the extent of and reasons for loss to follow-up if it occurred (96). A recent review indicates that insufficient blinding can lead to larger effect estimates and may therefore be an important source for potential bias (97). Thus, even though blinding may sometimes be difficult to implement in this type of trials, future trials should attempt to blind all persons involved as far as possible (conceivable options for blinding participants could, eg, be an attention control group or a placebo intervention) (98). Additionally, authors should refer to an a priori registered protocol and focus on concise reporting, eg, of the planned interventions and outcome assessment methods. Lastly, adverse events were collected in very few of the included studies and, apart from one study, only poorly reported. Future studies should adequately collect adverse events and precisely report assessment methods and number and nature of occurred events for all intervention groups.

### Practice implications

The findings from this systematic review indicate that interventions aimed at preventing back pain among office workers may lead to only small effects on back pain and work absence, which might be considered not practically meaningful in some contexts. When comparing all interventions that have been investigated in the included studies, physical activity and ergonomics appeared the most promising interventions. However, readers should keep in mind that the CoE is limited and that further research may lead to different results. As most of the included primary studies focused on mixed populations with and without baseline back pain, results from this systematic review can probably not unambiguously be transferred to people without back pain.

## Supplementary material

Supplementary material
