# Supplementary material for: Interventions for preventing back pain among office workers – a systematic review and network meta-analysis
Source: Scand J Work Environ Health. 2022 Dec 30;49(1):5–22. doi: 10.5271/sjweh.4070 (PMC10549919; doi:10.5271/sjweh.4070)
Supplement: Supplementary material [file SJWEH-49-5-S001.pdf]

Interventions for preventing back pain among office workers - a systematic review and network meta-analysis[1]

by Angelika Eisele-Metzger, PhD, Daria S Schoser, MEd, Meik D Klein, BSc, Kathrin Grummich, Health Sciences Dipl., Guido Schwarzer, PhD, Lukas Schwingshackl, PhD, Robin Hermann, PhD, Bianca Biallas, PhD, Christiane Wilke, PhD, Joerg J Meerpohl, MD, Cordula Braun, PhD [2]

1. Supplementary material
2. Correspondence to: Cordula Braun, PhD, Institute for Evidence in Medicine, Medical Center - University of Freiburg, Faculty of Medicine, University of Freiburg, Breisacher Straße 86, 79110 Freiburg, Germany. [E-mail: braun@ifem.uni-freiburg.de]

Supplementary material

Supplementary table S1. PRISMA NMA Checklist 3
Supplementary table S2. Deviations from the protocol 7
Supplementary table S3. Definitions of intervention categories 8
Supplementary table S4. Example search strategy 9
Supplementary table S5. Potentially relevant studies published in other languages 10
Supplementary table S6. Example of extracted data 11
Supplementary figure S1a-c. Forest plots for the pairwise comparisons 20
Supplementary table S7a-b. Leave-one-out meta-analyses to identify potential outliers 22
Supplementary figure S2a-b. Baujat plots for the leave-one-out meta-analyses to identify potential outliers 23
Supplementary table S8. Example of the analytical code 24
Supplementary table S9. Description of interventions 32
Supplementary figure S3a-c. Network graphs displaying overall risk of bias 39
Supplementary table S10a-c. GRADE evaluation for the network meta-analyses 41
Supplementary figure S4a-b. Forest plots for the network meta-analysis – outcome participants with back pain 44
Supplementary table S11. P-scores for the network meta-analysis – outcome participants with back pain 45
Supplementary table S12. Results of the component meta-analysis (additive model) – outcome participants with back pain 46
Supplementary figure S5. Network graph for the component meta-analysis – outcome participants with back pain 47
Supplementary table S13. Description of results for studies not included in quantitative synthesis 48

Supplementary figure S6a-b. Forest plots for the network meta-analysis – outcome back pain intensity ... 49

Supplementary table S14. P-scores for the network meta-analysis – outcome back pain intensity 50

Supplementary table S15. Results of the component meta-analysis (additive model) – outcome back pain intensity ... 51

Supplementary figure S7. Network graph for the component meta-analysis – outcome back pain intensity ... 52

Supplementary figure S8a-b. Forest plots for the network meta-analysis – outcome days of work absence ... 53

Supplementary table S16. P-scores for the network meta-analysis – outcome days of work absence ... 54

Supplementary table S17. Results of the component meta-analysis (additive model) - outcome days of work absence ... 54

Supplementary figure S9. Network graph for the component network meta-analysis – outcome days of work absence ... 55

Supplementary table S18. Intervention satisfaction ... 56

Supplementary figure S10a-c. Forest plots for the sensitivity analyses excluding studies with high risk of bias ... 58

Supplementary table S19a-c. P-scores for the sensitivity analyses excluding studies with high risk of bias ... 59

Supplementary figure S11a-g. Forest plots for the additional network meta-analyses for different localisations of back pain ... 60

Supplementary table S20a-g. P-scores for the additional network meta-analyses for different localisations of back pain ... 62

Supplementary figure S12a-b. Forest plots for the sensitivity analyses for medium and long term follow-up ... 63

Supplementary table S21a-b. P-scores for the sensitivity analyses for medium and long term follow-up ... 63

Supplementary figure S13a-b. Forest plot for the sensitivity analyses for different intervention durations ... 64

Supplementary table S22a-b. P-scores for the sensitivity analyses for different intervention durations ... 64

Supplementary figure S14. Comparison-adjusted funnel plot ... 65

References ... 66

Supplementary table S1. PRISMA NMA Checklist of Items to Include When Reporting A Systematic Review Involving a Network Meta-analysis (1).

Section/Topic	Item #	Checklist Item	Reported on Page #
**TITLE**			
Title	1	Identify the report as a systematic review *incorporating a network meta-analysis (or related form of meta-analysis).*	1
**ABSTRACT**			
Structured summary	2	Provide a structured summary including, as applicable:<br>**Background:** main objectives<br>**Methods:** data sources; study eligibility criteria, participants, and interventions; study appraisal; and *synthesis methods, such as network meta-analysis.*<br>**Results:** number of studies and participants identified; summary estimates with corresponding confidence/credible intervals; *treatment rankings may also be discussed. Authors may choose to summarize pairwise comparisons against a chosen treatment included in their analyses for brevity.*<br>**Discussion/Conclusions:** limitations; conclusions and implications of findings.<br>**Other:** primary source of funding; systematic review registration number with registry name.	1
**INTRODUCTION**			
Rationale	3	Describe the rationale for the review in the context of what is already known, *including mention of why a network meta-analysis has been conducted.*	3-4
Objectives	4	Provide an explicit statement of questions being addressed, with reference to participants, interventions, comparisons, outcomes, and study design (PICOS).	4
**METHODS**			
Protocol and registration	5	Indicate whether a review protocol exists and if and where it can be accessed (e.g., Web address); and, if available, provide registration information, including registration number.	4
Eligibility criteria	6	Specify study characteristics (e.g., PICOS, length of follow-up) and report characteristics (e.g., years considered, language, publication status) used as criteria for eligibility, giving rationale. *Clearly describe eligible treatments included in the treatment network, and note whether any have been clustered or merged into the same node (with justification).*	4-6
Information sources	7	Describe all information sources (e.g., databases with dates of coverage, contact with study authors to identify additional studies) in the search and date last searched.	6
Search	8	Present full electronic search strategy for at least one	Suppl.

		database, including any limits used, such that it could be repeated.	table 4
Study selection	9	State the process for selecting studies (i.e., screening, eligibility, included in systematic review, and, if applicable, included in the meta-analysis).	6-7
Data collection process	10	Describe method of data extraction from reports (e.g., piloted forms, independently, in duplicate) and any processes for obtaining and confirming data from investigators.	7-9
Data items	11	List and define all variables for which data were sought (e.g., PICOS, funding sources) and any assumptions and simplifications made.	7
**Geometry of the network**	**S1**	Describe methods used to explore the geometry of the treatment network under study and potential biases related to it. This should include how the evidence base has been graphically summarized for presentation, and what characteristics were compiled and used to describe the evidence base to readers.	9
Risk of bias within individual studies	12	Describe methods used for assessing risk of bias of individual studies (including specification of whether this was done at the study or outcome level), and how this information is to be used in any data synthesis.	7-8
Summary measures	13	State the principal summary measures (e.g., risk ratio, difference in means). *Also describe the use of additional summary measures assessed, such as treatment rankings and surface under the cumulative ranking curve (SUCRA) values, as well as modified approaches used to present summary findings from meta-analyses.*	8-9
Planned methods of analysis	14	Describe the methods of handling data and combining results of studies for each network meta-analysis. This should include, but not be limited to:<br>• *Handling of multi-arm trials;*<br>• *Selection of variance structure;*<br>• *Selection of prior distributions in Bayesian analyses; and*<br>• *Assessment of model fit.*	9-10
**Assessment of Inconsistency**	**S2**	Describe the statistical methods used to evaluate the agreement of direct and indirect evidence in the treatment network(s) studied. Describe efforts taken to address its presence when found.	11
Risk of bias across studies	15	Specify any assessment of risk of bias that may affect the cumulative evidence (e.g., publication bias, selective reporting within studies).	9
Additional analyses	16	Describe methods of additional analyses if done, indicating which were pre-specified. This may include, but not be limited to, the following:<br>• Sensitivity or subgroup analyses;<br>• Meta-regression analyses;<br>• *Alternative formulations of the treatment network; and*<br>• *Use of alternative prior distributions for Bayesian analyses (if applicable).*	10

**RESULTS†**			
Study selection	17	Give numbers of studies screened, assessed for eligibility, and included in the review, with reasons for exclusions at each stage, ideally with a flow diagram.	11
**Presentation of network structure**	**S3**	Provide a network graph of the included studies to enable visualization of the geometry of the treatment network.	Figure 3a-c
**Summary of network geometry**	**S4**	Provide a brief overview of characteristics of the treatment network. This may include commentary on the abundance of trials and randomized patients for the different interventions and pairwise comparisons in the network, gaps of evidence in the treatment network, and potential biases reflected by the network structure.	12-16
Study characteristics	18	For each study, present characteristics for which data were extracted (e.g., study size, PICOS, follow-up period) and provide the citations.	Table 1
Risk of bias within studies	19	Present data on risk of bias of each study and, if available, any outcome level assessment.	13
Results of individual studies	20	For all outcomes considered (benefits or harms), present, for each study: 1) simple summary data for each intervention group, and 2) effect estimates and confidence intervals. *Modified approaches may be needed to deal with information from larger networks.*	Table 1, Suppl. table 13
Synthesis of results	21	Present results of each meta-analysis done, including confidence/credible intervals. *In larger networks, authors may focus on comparisons versus a particular comparator (e.g. placebo or standard care), with full findings presented in an appendix. League tables and forest plots may be considered to summarize pairwise comparisons.* If additional summary measures were explored (such as treatment rankings), these should also be presented.	Table 2, Supple-ment
**Exploration for inconsistency**	**S5**	Describe results from investigations of inconsistency. This may include such information as measures of model fit to compare consistency and inconsistency models, *P* values from statistical tests, or summary of inconsistency estimates from different parts of the treatment network.	Suppl. figures 4a-b, 6a-b, 8a-b
Risk of bias across studies	22	Present results of any assessment of risk of bias across studies for the evidence base being studied.	17
Results of additional analyses	23	Give results of additional analyses, if done (e.g., sensitivity or subgroup analyses, meta-regression analyses*, alternative network geometries studied, alternative choice of prior distributions for Bayesian analyses,* and so forth).	16-17
**DISCUSSION**			
Summary of evidence	24	Summarize the main findings, including the strength of evidence for each main outcome; consider their relevance to key groups (e.g., healthcare providers, users, and policy-makers).	17-18

Limitations	25	Discuss limitations at study and outcome level (e.g., risk of bias), and at review level (e.g., incomplete retrieval of identified research, reporting bias). *Comment on the validity of the assumptions, such as transitivity and consistency. Comment on any concerns regarding network geometry (e.g., avoidance of certain comparisons).*	19-20
Conclusions	26	Provide a general interpretation of the results in the context of other evidence, and implications for future research.	18-21
**FUNDING**			
Funding	27	Describe sources of funding for the systematic review and other support (e.g., supply of data); role of funders for the systematic review. This should also include information regarding whether funding has been received from manufacturers of treatments in the network and/or whether some of the authors are content experts with professional conflicts of interest that could affect use of treatments in the network.	22

PICOS = population, intervention, comparators, outcomes, study design.
* Text in italics indicates wording specific to reporting of network meta-analyses that has been added to guidance from the PRISMA statement.
† Authors may wish to plan for use of appendices to present all relevant information in full detail for items in this section.

Supplementary table S2. Deviations from the protocol

**Inclusion criteria**	• The population was limited to office workers due to considerable heterogeneity of study populations and interventions identified, which would have precluded overall pooled analyses.<br>• Only studies that covered a follow-up period of at least 24 weeks from baseline were included as we considered this an appropriate time frame, given the focus of this review on primary prevention.<br>• Crossover trials were considered only if they included a follow-up of at least 24 weeks before crossover (using this and the other inclusion criteria, no crossover trial could be included).
**Data manage-ment**	• Based on the included studies, we slightly adapted our planned categorization of interventions, e.g. the category "behavioural intervention" was added.<br>• We extracted results for the primary outcomes for all reported time points ≥ 24 weeks from baseline (instead of all time points available).
**Assessment of risk of bias (RoB)**	• We did not assess RoB for secondary outcomes (i.e. adverse events and intervention satisfaction) as poor reporting of outcome assessment methods prevented a thorough RoB assessment for these outcomes.<br>• If assessment methods for the primary outcomes were comparable, outcomes were grouped and assessed together per study.
**Data synthesis**	• We conducted our main analyses using the results assessed closest to 12 months (instead of the results ≥ 12 months from baseline).<br>• In case of considerable heterogeneity for a pairwise comparison, we performed leave-one-out meta-analysis to identify potential outliers and excluded identified outliers from the pairwise meta-analyses as well as further network meta-analyses (instead of not performing meta-analyses for the respective comparison)<br>• We generated network graphs with coloured edges according to the overall assessment of RoB (instead of single RoB domains)
**Additional analyses**	• As there was no considerable heterogeneity in our analyses, we decided to not perform network meta-analysis regressions for mean age and the proportion of female participants.<br>The following analyses were planned but could not be performed for the given reasons:<br>• Subgroup analysis for job exposure (due to the above-mentioned change of population; all office workers had similar job exposure)<br>• Subgroup analysis for gender (as the included studies rarely reported gender-specific subgroups)<br>• Subgroup analysis for the presence of baseline back pain (as almost all studies included mixed populations of participants with and without baseline back pain)<br>• Sensitivity analysis for short-term follow-up /< 6 months from baseline (due to the above-mentioned requirement for the follow-up period)<br>• Sensitivity analysis for medium term (24 weeks to <12 months) and long term (after ≥12 months) follow-up for the outcomes back pain intensity (as there were too few studies with overlap for the same intervention categories) and days of work absence (as there were only studies with 12 month follow-up)<br>• Additional analyses considering different intervention durations for the outcomes back pain intensity and days of work absence (as there were too few studies with overlap for the same intervention categories)

Supplementary table S3. Definitions of intervention categories

Category	Specification
**Behavioural intervention**	Intervention addressing/promoting (health-related) behavioural changes, e.g. goal setting, action planning, problem solving, risk assessment and feedback (including provision of feedback devices such as activity trackers or a feedback mouse)
**Education**	Educational instruction or training, through e.g. lectures, presentations, group sessions, providing information or advice on various health-related aspects (e.g. benefits of physical activity or stress management)
**Ergonomics**	Intervention with specific focus on ergonomic aspects of the workplace and work environment, including ergonomic adjustments (e.g. of table heights or monitor positions); introduction of new equipment (e.g. arm support, ergonomic mouse) with instructions on use of equipment; specific ergonomic training (e.g. lectures on recommended workplace set-ups)
**Exercise equipment**	Provision of equipment at the workplace to stimulate exercise/physical activity (e.g. steppers, desk bikes) not as part of a specific physical activity/exercise intervention
**Physical activity**	Practical application of a physical activity/exercise intervention, individualised or group-based, supervised or unsupervised (e.g. supervised walking classes, individually-tailored exercise program)
**Multicomponent intervention with physical activity**	Intervention combining two or more different intervention components including physical activity (e.g. physical activity and behavioural intervention)
**Other multicomponent intervention**	Intervention combining two or more different intervention components (e.g. ergonomics and behavioural intervention; without physical activity)
**No/minimal intervention**	Comparison/control intervention; including sham intervention (e.g. inactive feedback mouse)

Supplementary table S4. Example search strategy for PubMed/MEDLINE

Search	Query
**#15**	Search: **#13 AND #14** Sort by: **Most Recent**
**#14**	Search: **randomized controlled trial[Publication Type] OR random allocation [MeSH Terms] OR controlled clinical trial[Title/Abstract] OR random*[Title/Abstract]** Sort by: **Most Recent**
**#13**	Search: **#9 OR #12** Sort by: **Most Recent**
**#12**	Search: **(#1 OR #10) AND #11** Sort by: **Most Recent**
**#11**	Search: **(prevent*[Title/Abstract] OR prophyla*[Title/Abstract])** Sort by: **Most Recent**
**#10**	Search: **back pain*[Title/Abstract] OR „ low back pain"[Title/Abstract] OR „back ache*"[Title/Abstract] OR back dysfunction*[Title/Abstract] OR back strain*[Title/Abstract] OR backache*[Title/Abstract] OR low back ache*[Title/Abstract] OR low back syndrome*[Title/Abstract] OR low backpain*[Title/Abstract] OR lowback pain*[Title/Abstract] OR lower back pain*[Title/Abstract] OR lower backache*[Title/Abstract] OR lower backpain*[Title/Abstract] OR lumbago*[Title/Abstract] OR lumbal pain*[Title/Abstract] OR lumbal syndrome*[Title/Abstract] OR lumbalgia*[Title/Abstract] OR lumbar pain*[Title/Abstract] OR lumbar spine syndrome*[Title/Abstract] OR lumbar syndrome*[Title/Abstract] OR lumbodynia*[Title/Abstract] OR lumbosacral pain[Title/Abstract] OR musculoskeletal pain[Title/Abstract]** Sort by: **Most Recent**
**#9**	Search: **#5 AND #8** Sort by: **Most Recent**
**#8**	Search: **#6 OR #7** Sort by: **Most Recent**
**#7**	Search: **Workplace[Title/Abstract] OR work setting[Title/Abstract] OR work site[Title/Abstract] OR work environment[Title/Abstract] OR industry[Title/Abstract] OR company[Title/Abstract] OR factory[Title/Abstract] OR office[Title/Abstract] OR offices[Title/Abstract] OR computer user*[Title/Abstract] OR laborer[Title/Abstract] OR employ*[Title/Abstract] OR personnel [Title/Abstract] OR occupation*[Title/Abstract] OR job [Title/Abstract] OR jobs[Title/Abstract] OR profession*[Title/Abstract] OR staff[Title/Abstract]** Sort by: **Most Recent**
**#6**	Search: **Workplace[MeSH Terms] OR Occupational Health[MeSH Terms] OR Occupational Diseases / prevention & control*** Sort by: **Most Recent**
**#5**	Search: **#1 OR #4** Sort by: **Most Recent**
**#4**	Search: **#2 AND #3** Sort by: **Most Recent**
**#3**	Search: **pain[Title/Abstract] OR discomfort[Title/Abstract] OR ache[Title/Abstract] OR sore*[Title/Abstract] OR injur*[Title/Abstract] OR symptom* [tiab] OR disorder* [tiab] OR problem* [tiab]** Sort by: **Most Recent**
**#2**	Search: **back[Title/Abstract] OR lumbar[Title/Abstract] OR neck[Title/Abstract] OR cervical[Title/Abstract] OR musculoskeletal[Title/Abstract]** Sort by: **Most Recent**
**#1**	Search: **Low Back Pain[MeSH Terms] OR Back Pain[MeSH Terms] OR Neck Pain[MeSH Terms] OR Musculoskeletal Pain[MeSH Terms]** Sort by: **Most Recent**

Supplementary table S5. Potentially relevant studies published in other languages

(records excluded for the reason “foreign language”)

Ferreira MB, Zanin LA, Ferreira VC, Barbosa D, Kerppers, II. Influência da ginástica laboral com base em exercícios de pilates na dor osteo muscular e qualidade do sono: estudo controlado, aleatório e randomizado. Revista Brasileira de Prescrição e Fisiologia do Exercício. 2019;13(87):1131-1140.
Kamerbeek-Buisman A, Kippersluis S. No measurable effect of lifting belt and lifting instructions for the prevention of low back pain at the workplace; a randomized, controlled trial. Nederlands tijdschrift voor geneeskunde. 1999;143(49):2490-2491.
Mohammadi Zeidi I, Mohammadi Zeidi B. The effect of stage-matched educational intervention on reduction in musculoskeletal disorders among computer users. Journal of babol university of medical sciences. 2012;14(SUPPL. 1):42-49.
Sadra Abarqhouei N, Hosseini Nasab H, Fakhrzad MB. Macro Ergonomics Interventions and their Impact on Productivity and Reduction of Musculoskeletal disorders: Including a Case Study. Iran Occupational Health. 2012;9(1):27-39.
Staal JB, Hlobil H, van Mechelen W. "Graded activity" for low back pain in company health care. Tijdschrift voor sociale gezondheidszorg. 1999;77(1):30.

Supplementary table S6. Example of extracted data

Identification			Study details			
**First author**	**Year of publication**	**Extracted by**	**Additional reports**	**Corresponding author**	**Contact details**	**Contact necessary?**
insert last name		*DS/AE/CB/MK*	*where applicable: insert last name and publication year for any additional reports (e.g. protocols) consulted for data extraction*	*insert last name, first name e.g. Mueller, An*	*insert e-mail-address*	*If applicable, insert issues requiring clarification with authors (e.g. lack of baseline data)*
**Brisson**	**1999**	DS, AE		Brisson, Chantal	chantal.brisson@gre.ulaval.ca	Baseline (T1) and follow-up (T2) values (n, % and total n) separately for the two study groups for prevalence of musculoskeletal symptoms of the neck-shoulder (measured by questionnaire) and prevalence of musculoskeletal symptoms of the lower back (measured by questionnaire) requested; data received from authors; added in data extraction
**Dalager**	**2017**	DS, AE	Sjogaard 2014, Justesen 2017	Dalager, Tina	tdalager@health.sdu.dk	2-year follow-up data for musculoskeletal pain (scale from 0 to 9) for neck, upper back and low back (each 3 mt and 7d) and sickness absence days requested; information from authors: 2 year data got lost in a data transfer

**Start date** (dd.mm.yy)	**End date** (dd.mm.yy)	**Study duration** (months)	**Study location**	**Funding sources**	**Details on funding sources**	**Potential conflicts of interest**	**Study design**	**Comments**
insert date of the first enrolment of participants (if no day is reported insert 1st; if other start date is reported, please note in the column "Comments")	*insert date of the last follow-up (if no day is reported insert last day of the month; if other end date is reported, please note in the column "Comments")*	*automatically calculated*	*insert country in which the study was conducted*	*as reported by the authors*	*choose from the list (if not apparent, look it up on the internet)*	*as reported by the authors (insert "none declared" if no conflicts are clearly reported)*	*choose from the list*	
01.01.94	30.06.96	30.4	Canada	Institut de Recherche en Santé et en Sécurité du Travail du Québec (IRSST), Social Sciences and Humanities Research Council of Canada (SSHRC), Fonds pour la Formation de Chercheurs et l'Aide à la Recherche (FCAR), national health research scholarship for Health Canada	Non-commercial	/	Cluster-RCT	exact start and end date not given: Data were collected over 30 months (1994-1996)
01.05.11	31.03.14	35.5	Denmark	companies Implement Consulting Group, PreviaSundhed, and the Simon Fougner Hartmanns Family Foundation	Commercial	none declared	RCT (parallel group)	

Population									
Inclusion/exclusion criteria				*Baseline characteristics*					
**Inclusion criteria** (individuals)	**Exclusion criteria** (individuals)	**Inclusion criteria** (cluster)	**Exclusion criteria** (cluster)	**Occupations / type(s) of work**	**Baseline back pain**	**Age**	**Female gender**	**Male gende r**	**Other/ divers e gender**
insert short description	*insert short description*	*insert short description (leave blank if no cluster)*	*insert short description (leave blank if no cluster)*	*(insert "not specified" if not reported)*	*Did participants suffer from back pain at baseline? (choose from the list)*	*mean years (SD) of the total sample e.g. 42.6 (8.3)*	*n (%) of the total sample e.g. 369 (51.9%)*	*n (%) of the total sample*	*n (%) of the total sample*
working 5 hours or more per week with a video display unit (VDU)	/	/	/	workers employed in a large university and in other institutions involved in university services (over 75% clerical workers)	yes	43	80%	/	/
Office workers who worked ≥25 h per week within an office environment	(a) cardiovascular disease, chest pain during physical exercise, myocardial infarction (lifetime history), stroke, severe musculoskeletal disorders, symptomatic herniated disc, and other severe disorders of the spine, postoperative conditions, or lifetime history of severe trauma and (b) pregnancy	/	/	office workers from six different companies located across Denmark: two private companies, two public municipalities, and two national boards	yes	44.0 (10.0)	74%	/	/

**Highest level of education 1**	**Education** baseline value 1	**Highest level of education 2**	**Education** baseline value 2	**Highest level of education 3**	**Education** baseline value 3	**Number of participants randomised**	**Number of participants randomised per intervention group**		**Comments**
insert first category	*n (%) e.g. 36 (21.2%)*	*insert second category*	*n (%)*	*insert third category*	*n (%)*	*total n randomised to the different intervention arms*	*n randomised to intervention 1*	*n randomised to intervention 2*	
/	/	/	/	/	/	774	/	/	n randomised calculated from p. 256 ("The 627 workers who participated in both the base line and the 6-month measurements represented 81% of the persons eligible at the base line")
/	/	/	/	/	/	387	193	194	

Interventions							
Intervention 1							
**Name**	**Intervention classification**	**Contents**	**Setting**	**Provider**	**Length of intervention period**	**Timing of intervention**	**Mode(s) of delivery of intervention**
insert name of first intervention	*choose from the list*	*insert short description of contents of intervention 1*	*insert work setting in which intervention 1 was delivered*	*insert who delivered/provided intervention 1*	*insert total duration of intervention 1 in months (1 month=30 days)*	*if applicable, insert number and duration of sessions or similar (e.g. one-hour-sessions, 3 times a week)*	*insert whether intervention 1 was e.g. group-based, one-to-one, online, telephone-based, text-message-based, self-directed, environmental…*
Ergonomic training program	Ergonomics	program based on to the PRECEDE (predisposing, reinforcing and enabling causes in educational diagnosis evaluation) model; targeting 3 types of behaviour: (i) adjusting the postural components of the workstation correctly; (ii) adjusting the visual components of the workstation correctly; and (iii) organising work activities in a preventive manner; a training guide was given to each participant; the sessions involved demonstrations, simulations, discussions, and lectures; + self-diagnosis of his (her) workstation using a photograph; each session was presented to about 15 workers with their supervisor at one time.	/	occupational health and safety professionals	0.5	2 sessions of 3 hours each at a 2-week interval	group-based, self-directed
Training group	Physical activity	training intervention, based on the theoretical framework of IPET; Each participant received an individually tailored exercise training program based on the baseline health check and questionnaire data; the exercise training program was performed during working hours, at or near the workplace; The program lasted one hour a week for 2 years, the first year was fully supervised, and, during the second year, monthly supervision of a weekly training session was provided; program included strength training and cardiorespiratory fitness training<br>+ participants in TG were encouraged by health ambassadors (peers) to engage in moderate physical activity	At or near the workplace	instructor was a sports science based exercise training specialist; health ambassadors were trained peers	24	one hour a week for 2 years	group-based, one-to-one

Intervention 2								
**Name**	**Intervention classification**	**Intervention classification - multicomponent**	**Contents**	**Setting**	**Provider**	**Length of interventio n period**	**Timing of intervention**	**Mode(s) of delivery of intervention**
insert name of second intervention	*choose from the list*	*for multicomponent interventions only*	*insert short description of contents of intervention 2*	*insert work setting in which intervention 2 was delivered*	*insert who delivered/ provided intervention 2*	*insert total duration of intervention 2 in months (1 month=30 days)*	*if applicable, insert number and duration of sessions or similar (e.g. one-hour-sessions, 3 times a week)*	*insert whether intervention 2 was e.g. group-based, one-to-one, online, telephon-based, text-message-based, self-directed, environmental…*
Reference group	No intervention / minimal intervention					0		
Control group	No intervention / minimal intervention		The participants in CG received no workplace physical exercise training or other information regarding recommended leisure time physical activity but were encouraged to maintain their lifestyle as usual			0		

**Outcomes**								
Participants with neck (-shoulder) pain								
**Outcome definition**	**Outcome measurement**	**Validation**	**Type of outcome**	**Unit of measuremen t**	**Categories / scale range**	**Directio n of outcome**	**Outcome assessor(s)**	**Time points of assessment**
insert definition of outcome (e.g. numbers of participants with at least one new low back pain episode during the follow-up period)	*insert short description of measurement instrument*	*Is the outcome measurement instrument validated? (choose from the list)*	*choose from the list*	*if applicable (for continuous outcomes)*	*if applicable (e.g. no pain/pain; 0-10)*	*if applicable: choose from the list*	*insert who assessed the outcome (e.g. researcher, physician, questionnaire)*	*insert all time points reported (if other than "from baseline", define reference point, e.g. from the end of intervention)*
Prevalence of musculoskeletal symptoms of the neck-shoulder	prevalent symptoms were defined as those which were present on 3 days or more during the last 7 days and for which the intensity of pain was greater than half the visual analogue scale (VAS)	not specified	dichotomous	/	pain / no pain		questionnaire	baseline and 6 months

Participants with lower back pain								
**Outcome definition**	**Outcome measurement**	**Validation**	**Type of outcome**	**Unit of measurement**	**Categories / scale range**	**Direction of outcome**	**Outcome assessor(s)**	**Time points of assessment**
insert definition of outcome (e.g. numbers of participants with at least one new low back pain episode during the follow-up period)	*insert short description of measurement instrument*	*Is the outcome measurement instrument validated? (choose from the list)*	*choose from the list*	*if applicable (for continuous outcomes)*	*if applicable (e.g. no pain/pain; 0-10)*	*if applicable: choose from the list*	*insert who assessed the outcome (e.g. researcher, physician, questionnaire)*	*insert all time points reported (if other than "from baseline", define reference point, e.g. from the end of intervention)*
Prevalence of musculoskeletal symptoms of the lower back	prevalent symptoms were defined as those which were present on 3 days or more during the last 7 days and for which the intensity of pain was greater than half the visual analogue scale (VAS)	not specified	dichotomous	/	pain / no pain		questionnaire	baseline and 6 months

Neck (-shoulder) pain intensity								
**Outcome definition**	**Outcome measurement**	**Validation**	**Type of outcome**	**Unit of measurement**	**Categories / scale range**	**Direction of outcome**	**Outcome assessor(s)**	**Time points of assessment**
insert definition of outcome (e.g. intensity of low back pain)	*insert short description of measurement instrument*	*Is the outcome measurement instrument validated? (choose from the list)*	*choose from the list*	*if applicable (for continuous outcomes)*	*if applicable (e.g. no pain/pain; 0-10)*	*if applicable: choose from the list*	*insert who assessed the outcome (e.g. researcher, physician, questionnaire)*	*insert all time points reported (if other than "from baseline", define reference point, e.g. from the end of intervention)*
Musculoskeletal pain in neck - past 7 days	participants rated their pain intensity, "on average, how intense was your pain in the neck during the past 7 days?," on a 10-point numerical box scale ranging from 0 (no pain) to 9 (worst possible pain); Nordic Musculoskeletal Questionnaire	not specified	continuous		0-9	lower is better	questionnaire	baseline, one year, two years (two year results not reported)

Lower back pain intensity								
**Outcome definition**	**Outcome measurement**	**Validation**	**Type of outcome**	**Unit of measurement**	**Categories / scale range**	**Direction of outcome**	**Outcome assessor(s)**	**Time points of assessment**
insert definition of outcome (e.g. intensity of neck pain)	*insert short description of measurement instrument*	*Is the outcome measurement instrument validated? (choose from the list)*	*choose from the list*	*if applicable (for continuous outcomes)*	*if applicable (e.g. no pain/pain; 0-10)*	*if applicable: choose from the list*	*insert who assessed the outcome (e.g. researcher, physician, questionnaire)*	*insert all time points reported (if other than "from baseline", define reference point, e.g. from the end of intervention)*
Musculoskeletal pain in lower back past 7 days	participants rated their pain intensity, "on average, how intense was your pain in the lower back during the past seven days?," on a 10-point numerical box scale ranging from 0 (no pain) to 9 (worst possible pain); Nordic Musculoskeletal Questionnaire	not specified	continuous		0-9	lower is better	questionnaire	baseline, one year, two years (two year results not reported)

Upper back pain intensity								
**Outcome definition**	**Outcome measurement**	**Validation**	**Type of outcome**	**Unit of measurement**	**Categories / scale range**	**Direction of outcome**	**Outcome assessor(s)**	**Time points of assessment**
insert definition of outcome (e.g. intensity of neck pain)	*insert short description of measurement instrument*	*Is the outcome measurement instrument validated? (choose from the list)*	*choose from the list*	*if applicable (for continuous outcomes)*	*if applicable (e.g. no pain/pain; 0-10)*	*if applicable: choose from the list*	*insert who assessed the outcome (e.g. researcher, physician, questionnaire)*	*insert all time points reported (if other than "from baseline", define reference point, e.g. from the end of intervention)*
Musculoskeletal pain in upper back past 7 days	participants rated their pain intensity, "on average, how intense was your pain in the upper back during the past 7 days?," on a 10-point numerical box scale ranging from 0 (no pain) to 9 (worst possible pain) Nordic Musculoskeletal Questionnaire	not specified	continuous		0-9	lower is better	questionnaire	baseline, one year, two years (two year results not reported)

Days of work absence								
**Outcome definition**	**Outcome measurement**	**Validation**	**Type of outcome**	**Unit of measuremen t**	**Categories / scale range**	**Direction of outcome**	**Outcome assessor(s)**	**Time points of assessment**
insert definition of outcome (e.g. numbers of days with work absenteeism due to back pain during the follow-up period)	*insert short description of measurement instrument*	*Is the outcome measurement instrument validated? (choose from the list)*	*choose from the list*	*if applicable (for continuous outcomes) (e.g. days absent)*	*if applicable (e.g. not absent/absent; 0-10)*	*if applicable: choose from the list*	*insert who assessed the outcome (e.g. researcher, physician, questionnaire)*	*insert all time points reported (if other than "from baseline", define reference point, e.g. from the end of intervention)*
Sickness absence days	Absence data were accrued by years and months, and care days, weekends, and child first and second day of illness were removed. The focus of this study is short-term SA (periods of 1 to 10 days' absence); thus, long-term SA (11 days, which is the official cut-off point in Denmark) and part-time leave were discarded before analysis	no	continuous	days	/		Human Resources managers of the companies (Company Registration Data)	1 year before start of the intervention; 1 year follow up

Identification		Results								
**First author**	**Year of publication**	**Outcome**	**Time frame**	**For pain intensity: Scale range**		**Intervention**	**Intervention category**	**Length of intervention period**	**Time point**	**N**
insert last name		*choose the respective outcome from the list*	*to which the outcome measurement refers (e.g. pain intensity in the last 7 days)*	*lower value*	*upper value*	*choose the respective intervention from Study_Charact and link it using "= " (F4)*		*total duration of intervention in months (1 month = 30 days)*	*point of measurement from baseline in months (baseline=0)*	*insert number of participants for this outcome, intervention group and time point*
**author**	**year**	**outcome**	**timeframe**	**scale.lower**	**scale.upper**	**intervention**	**int.cat**	**int.duration**	**timepoint**	**n**
**Brisson**	**1999**	P with lower back pain	7 days			Ergonomic training program	Ergonomics	0.5	6	283
**Brisson**	**1999**	P with lower back pain	7 days			Reference group	No intervention / minimal intervention	0	6	339
**Brisson**	**1999**	P with lower back pain	7 days			Ergonomic training program	Ergonomics	0.5	0	278
**Brisson**	**1999**	P with lower back pain	7 days			Reference group	No intervention / minimal intervention	0	0	341
**Brisson**	**1999**	P with neck (-shoulder) pain	7 days			Ergonomic training program	Ergonomics	0.5	6	282
**Brisson**	**1999**	P with neck (-shoulder) pain	7 days			Reference group	No intervention / minimal intervention	0	6	341
**Brisson**	**1999**	P with neck (-shoulder) pain	7 days			Ergonomic training program	Ergonomics	0.5	0	275
**Brisson**	**1999**	P with neck (-shoulder) pain	7 days			Reference group	No intervention / minimal intervention	0	0	343
**Dalager**	**2017**	Days of work absence	12 months			Control group	No intervention / minimal intervention	0	12	194
**Dalager**	**2017**	Days of work absence	12 months			Training group	Physical activity	24	12	193
**Dalager**	**2017**	Days of work absence	12 months			Control group	No intervention / minimal intervention	0	0	194
**Dalager**	**2017**	Days of work absence	12 months			Training group	Physical activity	24	0	193
**Dalager**	**2017**	Lower back pain intensity	7 days	0	9	Control group	No intervention / minimal intervention	0	12	194
**Dalager**	**2017**	Lower back pain intensity	7 days	0	9	Training group	Physical activity	24	12	193
**Dalager**	**2017**	Neck (-shoulder) pain intensity	7 days	0	9	Control group	No intervention / minimal intervention	0	12	194
**Dalager**	**2017**	Neck (-shoulder) pain intensity	7 days	0	9	Training group	Physical activity	24	12	193
**Dalager**	**2017**	Upper back pain intensity	7 days	0	9	Control group	No intervention / minimal intervention	0	12	194
**Dalager**	**2017**	Upper back pain intensity	7 days	0	9	Training group	Physical activity	24	12	193

For continuous outcome							For dichotomous outcome	
**Define mean score**	**Mean**	**SD**	**SE**	**95% CI**		**Median**	**n**	**%**
choose from the list	*insert mean value*	*insert standard deviation (if applicable)*	*insert standard error (if applicable)*	*insert 1st value of 95% confidence interval (if applicable)*	*insert 2nd value of 95% confidence interval (if applicable)*	*insert median (if applicable)*	*insert number of participants with event (e.g. n of participants with back pain)*	*insert % of participants with event (e.g. % of participants with back pain)*
**mean.details**	**mean**	**sd**	**se**	**lower**	**upper**	**median**	**n.event**	**p.event**
							22	7.77
							24	7.08
							28	10.07
							26	7.62
							36	12.77
							46	13.49
							30	10.91
							49	14.29
Mean change score from baseline	-0.1	4.4						
Mean change score from baseline	-1.2	5.3						
Mean score at measurement point	3.6	4.7						
Mean score at measurement point	4.4	6.3						
Mean change score from baseline	-0.5	1.8						
Mean change score from baseline	-0.7	1.9						
Mean change score from baseline	-0.7	1.8						
Mean change score from baseline	-0.9	2						
Mean change score from baseline	-0.6	1.4						
Mean change score from baseline	-0.7	1.7						

Supplementary figure S1a-c. Forest plots for the pairwise comparisons

a) Outcome participants with back pain

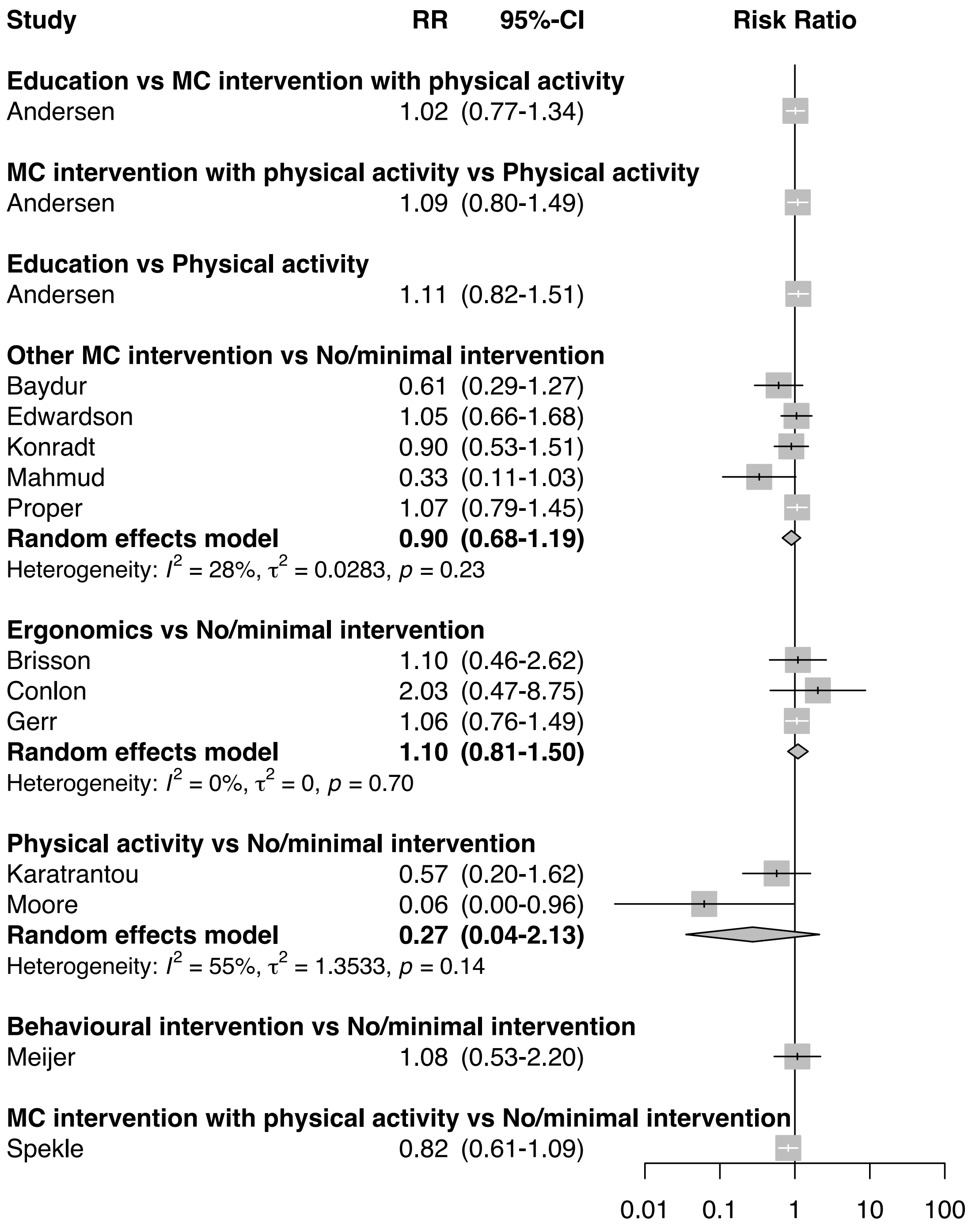

b) Outcome back pain intensity

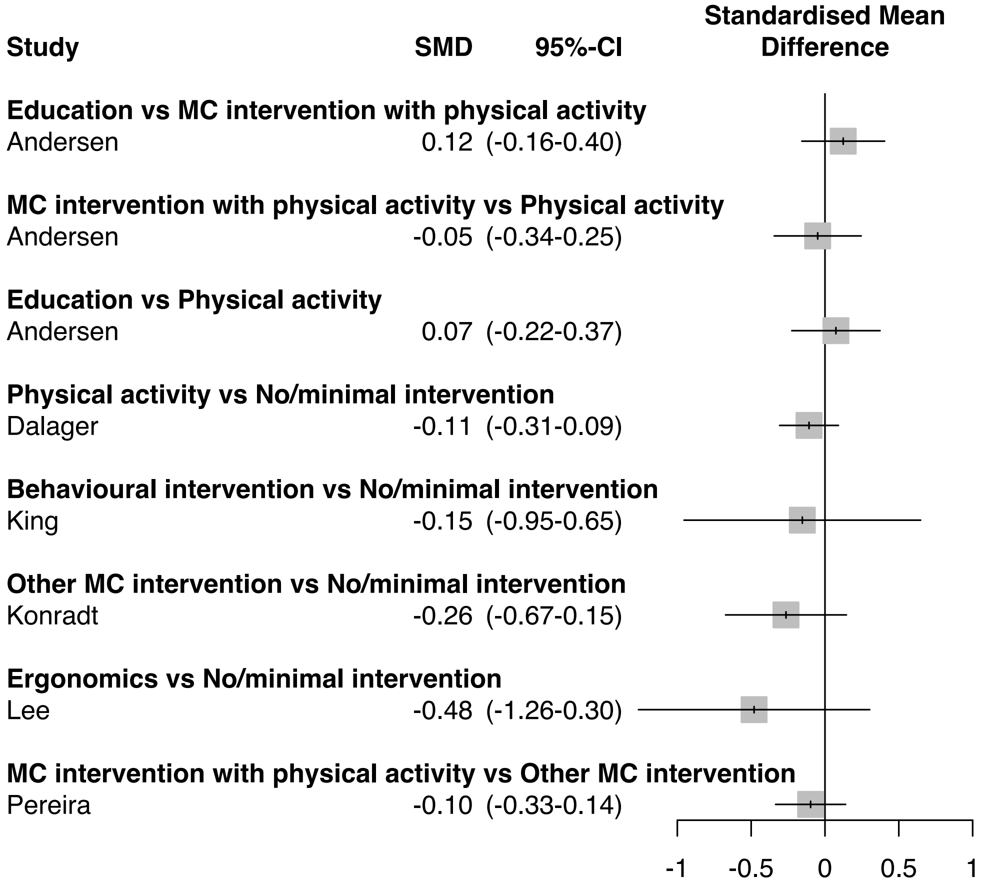

c) Outcome days of work absence

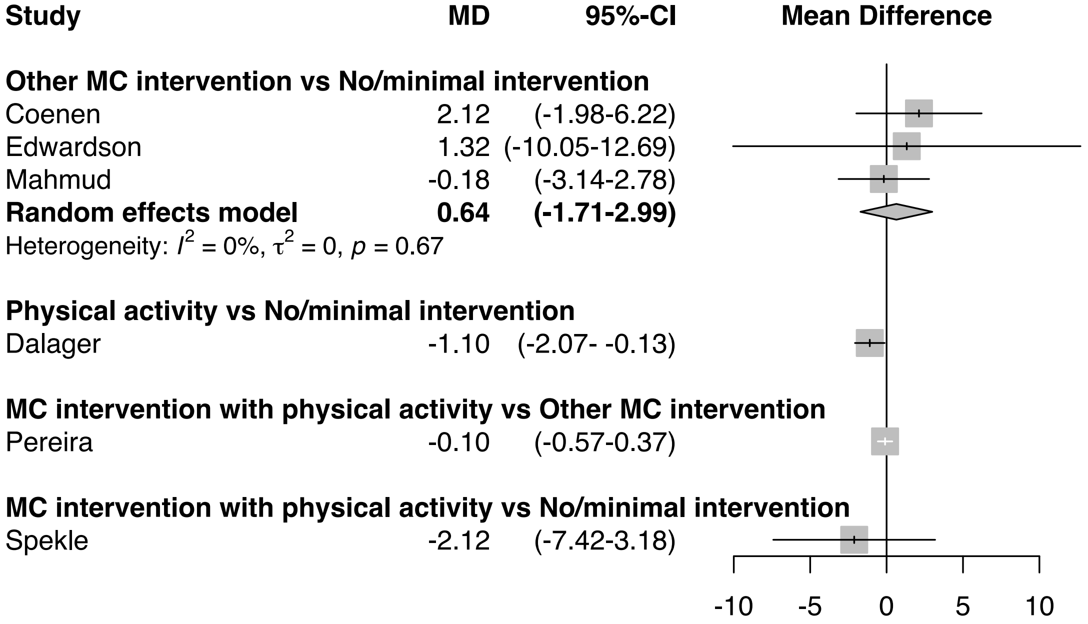

RR: risk ratio (RR<1 is beneficial); CI: confidence interval; MC: multicomponent; SMD: standardised mean difference (negative values are beneficial); MD: mean difference (negative values are beneficial).

Supplementary table S7a-b. Leave-one-out meta-analyses to identify potential outliers

a) Outcome back pain intensity

	SMD (95%-CI)	p-value	tau^2	tau	I^2
Omitting Dalager	-0.77 (-1.64-0.10)	0.0839	0.6576	0.8109	84.6%
Omitting Karatrantou	-0.15 (-0.33-0.02)	0.0753	0.0000	0.0000	0.0%
Omitting King	-0.69 (-1.37- -0.01)	0.0477	0.3977	0.6307	88.0%
Omitting Konradt	-0.72 (-1.61-0.17)	0.1128	0.6985	0.8357	88.1%
Omitting Lee	-0.62 (-1.30-0.06)	0.0749	0.3947	0.6283	87.9%
Pooled estimate	-0.58 (-1.16- -0.01)	0.0459	0.3274	0.5722	84.1%

b) Outcome days of work absence

	MD (95%-CI)	p-value	tau^2	tau	I^2
Omitting Coenen	-1.99 (-4.12-0.15)	0.0681	3.0107	1.7351	61.4%
Omitting Dalager	-1.32 (-4.51-1.87)	0.4165	7.8410	2.8002	66.8%
Omitting Edwardson	-1.47 (-3.64-0.69)	0.1818	3.7507	1.9367	69.4%
Omitting Karatrantou	-0.88 (-1.77-0.00)	0.0507	0.0000	0.0000	0.0%
Omitting Mahmud	-1.63 (-4.22-0.95)	0.2145	4.7757	2.1853	68.3%
Omitting Speklé	-1.28 (-3.61-1.04)	0.2803	4.0897	2.0223	69.7%
Pooled estimate	-1.40 (-3.46-0.65)	0.1809	3.3637	1.8340	62.4%

SMD: standardised mean difference; CI: confidence interval; MD: mean difference.

Supplementary figure S2a-b. Baujat plots for the leave-one-out meta-analyses to identify potential outliers

a) Outcome back pain intensity

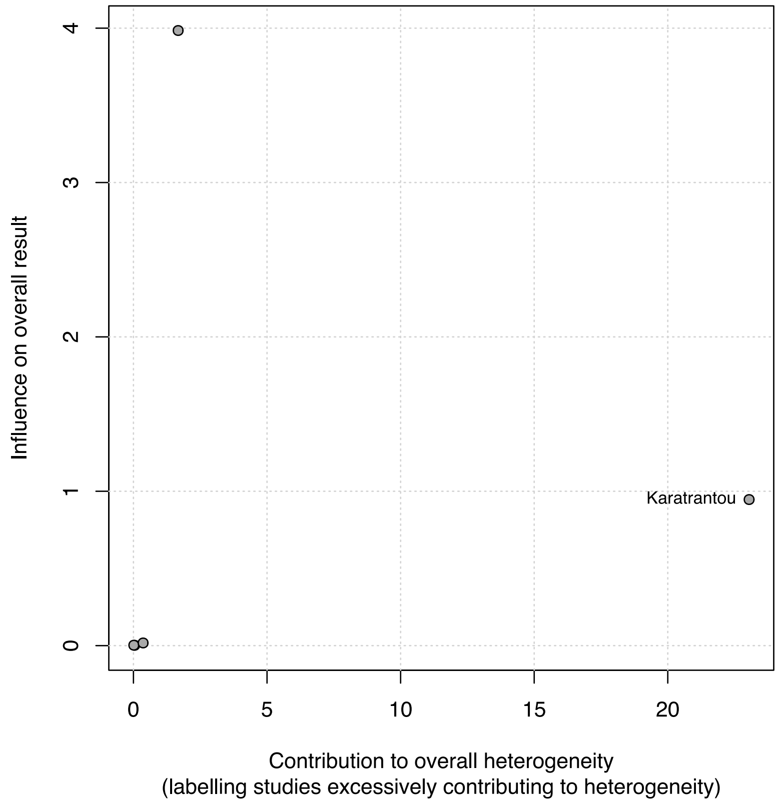

b) Outcome days of work absence

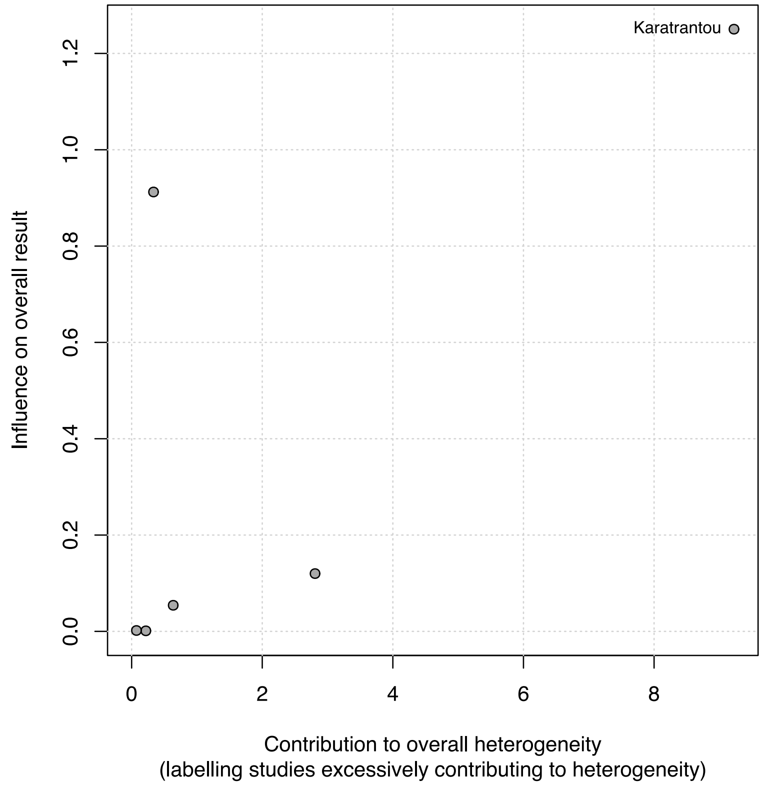

Supplementary table S8. Example of the analytical code

Outcome back pain intensity

```
---
title: 'Praev-Rueck'
subtitle: 'Outcome: Back pain intensity'
author: 'G. Schwarzer, IMBI, Freiburg, 23.03.2022'
output:
  word_document:
    fig_height: 7
    fig_width: 10
---

```{r setup, include = FALSE}


knitr::opts_chunk$set(eval = TRUE, echo = TRUE, message = FALSE, warning =
FALSE)
```

```{r settings, include = FALSE}
library(netmeta)
settings.meta(fixed = FALSE, digits = 2,
  test.subgroup = FALSE, method.tau = "DL")
##
cRCTs <- c("Andersen", "Baydur", "Brisson", "Coenen", "Edwardson",
  "Jelsma", "Lee", "Mahmud", "Pereira", "Spekle")
```

```{r readdata, include = FALSE}
load("bpi.rda")
```


Available data for all timepoints

```{r alldata, echo = FALSE}
bpi$diff <- bpi$timepoint - 12
bpi[, c("author", "timepoint", "diff", "int.mc.pa", "n")]
```


Data closest to 12 months

```{r data12, echo = FALSE}
bpi <- subset(bpi,
  !(author == "Andersen" & timepoint == 0) &
  !(author == "Karatrantou" & timepoint == 0) &
  !(author == "King" & timepoint == 0) &
  !(author == "Konradt" & timepoint %in% c(0, 24)) &
  !(author == "Lee" & timepoint %in% c(0, 6)) &
  !(author == "Pereira" & timepoint == 0))
bpi[, c("author", "timepoint", "diff", "int.mc.pa", "n")]
```
```

````
## Data to calculate SMDs

```{r smddata, echo = FALSE}
vars <- c("author", "n", "mean", "sd", "se", "lower", "upper", "median")
bpi[, vars]
```


## Impute median of available SDs

```{r imputesd, echo = FALSE}
bpi$sd.orig <- bpi$sd
bpi$sd[is.na(bpi$sd)] <- median(bpi$sd, na.rm = TRUE)
vars <- c("author", "n", "mean", "sd", "sd.orig")
bpi[is.na(bpi$sd.orig), vars]
```


##  Effektive sample size for cluster RCTs

- *N / designeffect* with *designeffect = 1 + (M - 1) x ICC*

```{r neff, echo = FALSE}
bpi$M <- ifelse(bpi$author %in% cRCTs, bpi$n.total / bpi$n.cluster, 1)
##
bpi$deseff.0.05 <- 1 + (bpi$M - 1) * 0.05
bpi$deseff.0.02 <- 1 + (bpi$M - 1) * 0.02
##
bpi$n.icc0.05 <- bpi$n / bpi$deseff.0.05
bpi$n.icc0.02 <- bpi$n / bpi$deseff.0.02
##
bpi$M <- round(bpi$M, 2)
```

### ICC = 0.05

```{r printeff.0.05, echo = FALSE}
tmp <- bpi
tmp$n.icc0.05 <- round(bpi$n.icc0.05, 1)
tmp$deseff.0.05 <- round(bpi$deseff.0.05, 3)
tmp[tmp$M != 1, c("author", "n", "n.icc0.05", "deseff.0.05", "n.total",
"n.cluster", "M")]
```

### ICC = 0.02

```{r printeff.0.02, echo = FALSE}
tmp <- bpi
tmp$n.icc0.02 <- round(bpi$n.icc0.02, 1)
tmp$deseff.0.02 <- round(bpi$deseff.0.02, 3)
tmp[tmp$M != 1, c("author", "n", "n.icc0.02", "deseff.0.02", "n.total",
"n.cluster", "M")]
```
````

```
## Pairwise meta-analysis - active vs no / minimal intervention (ICC =
0.05)

```{r ma.0.05, echo = FALSE}
p.icc0.05 <-
  pairwise(studlab = author, n = n.icc0.05, mean = mean, sd = sd,
    treat = int.mc.pa,
    data = bpi, sm = "SMD")
##
p.icc0.05$t1 <-
  ifelse(p.icc0.05$treat1 != "No/minimal intervention",
    "Active", p.icc0.05$treat1)
p.icc0.05$t2 <-
  ifelse(p.icc0.05$treat2 != "No/minimal intervention",
    "Active", p.icc0.05$treat2)
##
sel <- p.icc0.05$t1 != "Active"
tmp.t2 <- p.icc0.05$t2[sel]
p.icc0.05$t2[sel] <- p.icc0.05$t1[sel]
p.icc0.05$t1[sel] <- tmp.t2
p.icc0.05$TE[sel] <- -p.icc0.05$TE[sel]
##
p.icc0.05[, c("author", "t1", "t2")]
##
m.icc0.05 <-
  metagen(TE, seTE, data = p.icc0.05, sm = "SMD",
    studlab = author, subset = t1 != "Active" | t2 != "Active")
summary(m.icc0.05)
```

Search for potential outliers

```{r l1o.0.05, echo = FALSE}
metainf(m.icc0.05)
baujat(m.icc0.05, studlab = 4)
title(main = "Baujat plot",
  sub = "(labelling studies excessively contributing to heterogeneity)")
```

Results without Karatrantou

```{r dropkara.0.05, echo = FALSE}
update(m.icc0.05,
  subset = author != "Karatrantou" & (t1 != "Active" | t2 != "Active"))
```


Pairwise meta-analysis - active vs no / minimal intervention (ICC =
0.02)

```{r ma.0.02, echo = FALSE}
p.icc0.02 <-
  pairwise(studlab = author, n = n.icc0.02, mean = mean, sd = sd,
    treat = int.mc.pa,
    data = bpi, sm = "SMD")
##
```
```

```
p.icc0.02$t1 <-
  ifelse(p.icc0.02$treat1 != "No/minimal intervention",
    "Active", p.icc0.02$treat1)
p.icc0.02$t2 <-
  ifelse(p.icc0.02$treat2 != "No/minimal intervention",
    "Active", p.icc0.02$treat2)
##
sel <- p.icc0.02$t1 != "Active"
tmp.t2 <- p.icc0.02$t2[sel]
p.icc0.02$t2[sel] <- p.icc0.02$t1[sel]
p.icc0.02$t1[sel] <- tmp.t2
p.icc0.02$TE[sel] <- -p.icc0.02$TE[sel]
##
m.icc0.02 <-
  metagen(TE, seTE, data = p.icc0.02, sm = "SMD",
    studlab = author,
    subset = author != "Karatrantou" & (t1 != "Active" | t2 != "Active"))
summary(m.icc0.02)
```

Network meta-analysis (ICC = 0.05)

```{r icc.0.05, echo = FALSE}
bpi.nokara <- subset(bpi, author != "Karatrantou")
##
p.icc0.05 <-
  pairwise(studlab = author, n = n.icc0.05, mean = mean, sd = sd,
    treat = int.mc.pa,
    data = bpi.nokara, sm = "SMD")
##
net.icc0.05 <- netmeta(p.icc0.05, reference = "No/minimal")
cat("TE = SMD; seTE = seSMD\n")
print(summary(net.icc0.05), nchar.trts = 15,
  nma = FALSE, legend = FALSE, random = FALSE)
net.icc0.05
##
labs <- gsub("minimal ", "minimal\n", net.icc0.05$trts, fixed = TRUE)
netgraph(net.icc0.05, seq = "o",
  plastic = FALSE, number = TRUE,
  points = TRUE, cex.points = 10 * sqrt(n.trts / max(n.trts)),
  thickness = "se.fixed", col = "black",
  labels = paste0(labs, "\n(n=", round(n.trts), ")"),
  offset = 0.04)
##
forest(net.icc0.05)

ord <- c("MC intervention with physical activity",
  "Physical activity",
  "Other MC intervention",
  "Ergonomics",
  "Behavioural intervention",
  "Education",
  "No/minimal intervention")
nl.icc0.05 <- netleague(net.icc0.05, seq = ord, digits = 2)
writexl::write_xlsx(nl.icc0.05$random,
```

```
  path = "bpi-netleague-icc0.05.xlsx",
  col_names = FALSE)

grade.icc0.05 <-
  nettable(net.icc0.05, order = ord, digits = 2, text.NA = "")
```

```
### P-scores

```{r ps.0.05, echo = FALSE}
netrank(net.icc0.05, small.values = "good")
```

Evaluation of inconsistency

```{r dd.0.05, echo = FALSE}
decomp.design(net.icc0.05, nchar.trts = 21)
```

Comparison of direct and indirect evidence

```{r ns.0.05, echo = FALSE}
ns.icc0.05 <- netsplit(net.icc0.05, sep.trts = " vs ")
print(ns.icc0.05, nchar.trts = 10, indent = FALSE)
```

```{r forest.ns.0.05, echo = FALSE, fig.height = 9}
forest(ns.icc0.05, show = "with.direct",
  leftcols = c("studlab", "k", "prop", "effect", "ci"),
  rightcols = FALSE,
  col.by = "black")
```


Network graph with ROB2

```{r netgraph.rob2.0.05, echo = FALSE}
col.rob2 <- netmatrix(net.icc0.05, rob2, ties.method = "last",
  levels = 2:3, labels = c("yellow", "red"))
netgraph(net.icc0.05, seq = "o",
  plastic = FALSE, number = TRUE,
  points = TRUE, cex.points = 10 * sqrt(n.trts / max(n.trts)),
  col.points = "black",
  thickness = "se.fixed", col = col.rob2,
  labels = paste0(labs, "\n(n=", round(n.trts), ")"),
  offset = 0.04)
```


Sensitivity analysis: exclude studies with high overall RoB

```{r sens.rob2.some, echo = FALSE}
net.rob2.some <- netmeta(subset(p.icc0.05, rob2 == 2), ref = "No/minimal")
cat("TE = SMD; seTE = seSMD\n")
print(summary(net.rob2.some), nchar.trts = 15,
      nma = FALSE, legend = FALSE, random = FALSE)
```
```

```
net.rob2.some
labs <- net.rob2.some$trts
netgraph(net.rob2.some, seq = "o",
  plastic = FALSE, number = TRUE,
  points = TRUE, cex.points = 10 * sqrt(n.trts / max(n.trts)),
  col.points = "black",
  thickness = "se.fixed", col = "yellow",
  labels = paste0(labs, "\n(n=", round(n.trts), ")"),
  offset = 0.04)
forest(net.rob2.some)
netrank(net.rob2.some, small.values = "good")
```

All pairwise comparisons

```{r npw.0.05, echo = FALSE, fig.height = 8.5}
summary(netpairwise(net.icc0.05, separate = TRUE, sep.trts = " vs "))
forest(netpairwise(net.icc0.05, sep.trts = " vs ", reference.group =
"No/minimal"),
  leftcols = c("studlab", "effect", "ci"),
  rightcols = FALSE,
  colgap.studlab = "2cm", col.by = "black")
```

Network meta-analysis (ICC = 0.02)

```{r nma.0.02, echo = FALSE}
p.icc0.02 <-
  pairwise(studlab = author, n = n.icc0.02, mean = mean, sd = sd,
    treat = int.mc.pa, data = bpi.nokara, sm = "SMD")
##
net.icc0.02 <- netmeta(p.icc0.02, reference = "No/minimal")
net.icc0.02
##
forest(net.icc0.02)

nl.icc0.02 <- netleague(net.icc0.02, seq = ord, digits = 2)
writexl::write_xlsx(nl.icc0.02$random,
  path = "bpi-netleague-icc0.02.xlsx",
  col_names = FALSE)

grade.icc0.02 <-
  nettable(net.icc0.02, order = ord, digits = 2, text.NA = "")
```

P-scores

```{r ps.0.02, echo = FALSE}
netrank(net.icc0.02, small.values = "good")
```

Evaluation of inconsistency

```{r dd.0.02, echo = FALSE}
```

````
decomp.design(net.icc0.02, nchar.trts = 21)
```

### Comparison of direct and indirect evidence

```{r ns.0.02, echo = FALSE}
ns.icc0.02 <- netsplit(net.icc0.02, sep.trts = " vs ")
print(ns.icc0.02, nchar.trts = 10, indent = FALSE)
```

```{r forest.ns.0.02, echo = FALSE, fig.height = 9}
forest(ns.icc0.02, show = "with.direct",
  leftcols = c("studlab", "k", "prop", "effect", "ci"),
  rightcols = FALSE,
  col.by = "black")
```


### All pairwise comparisons

```{r npw.0.02, echo = FALSE, fig.height = 8.5}
summary(netpairwise(net.icc0.02, separate = TRUE, sep.trts = " vs "))
forest(netpairwise(net.icc0.02, sep.trts = " vs ", reference.group =
"No/minimal"),
  leftcols = c("studlab", "effect", "ci"),
  rightcols = FALSE,
  colgap.studlab = "2cm", col.by = "black")
```


## Component network meta-analysis (ICC = 0.05)

```{r icc.0.05.c, echo = FALSE}
p.icc0.05.c <-
  pairwise(studlab = author, n = n.icc0.05, mean = mean, sd = sd,
    treat = int.comps,
    data = bpi.nokara, sm = "SMD")
##
net.icc0.05.c <-
  netmeta(p.icc0.05.c, reference = "No/minimal")
cat("TE = SMD; seTE = seSMD\n")
print(summary(net.icc0.05.c), nchar.trts = 15,
      nma = FALSE, legend = FALSE, random = FALSE)
print(net.icc0.05.c, nchar.trts = 25)
labs <- gsub("+ ", "+\n", net.icc0.05.c$trts, fixed = TRUE)
netgraph(net.icc0.05.c, seq = "o",
  plastic = FALSE, number = TRUE,
  points = TRUE, cex.points = 10 * sqrt(n.trts / max(n.trts)),
  thickness = "se.fixed", col = "black",
  labels = paste0(labs, "\n(n=", round(n.trts), ")"),
  offset = 0.04)
forest(net.icc0.05.c)

netcomb(net.icc0.05.c, inactive = "No/minimal", nchar.comps = 4)
```
````
````

```
## Component network meta-analysis (ICC = 0.02)

```{r icc.0.02.c, echo = FALSE}
p.icc0.02.c <-
  pairwise(studlab = author, n = n.icc0.02, mean = mean, sd = sd,
    treat = int.comps, data = bpi.nokara, sm = "SMD")
##
net.icc0.02.c <-
  netmeta(p.icc0.02.c, reference = "No/minimal", nchar.trts = 25)
net.icc0.02.c
forest(net.icc0.02.c)

netcomb(net.icc0.02.c, inactive = "No/minimal", nchar.comps = 4)
```
```

Supplementary table S9. Description of interventions

Study	Intervention	Classifi-cation	Description	Provider	Length (months)
**Andersen 2008**	Specific resistance training	PA	Group-based training program for the neck and shoulder muscles, conducted three times per week, 20 minutes per training session.	Experienced instructors	12
	All-round physical exercise	MCIPA (EDU, BI, EEQ, PA)	Introduction of different forms of activities; participants were motivated to do different physical activities during leisure and work and made to fill out a contract indicating planned activities; different items and groups were installed/ initiated (e.g. steppers, punching bags, group sessions of Nordic walking); instructors visited one to four times a month.	Experienced instructors	12
	Reference intervention	EDU	Participants were encouraged to form groups that were asked to try to improve health and working conditions (participants themselves were responsible for organisation); participants received support from the study staff and an equal amount of attention compared to the other groups.	Study staff	12
**Baydur 2016**	Participatory ergonomic intervention	MCI (EDU, BI, ERG)	2-hour session introducing ergonomics, musculoskeletal disorders, adaptation of the work environment, implementation of exercises and relaxation and risk assessment; one month later, participants were visited at work, their individual risk was assessed using a checklist, solutions were jointly developed and implementation was planned.	Researchers	2
	Control group	NI	No intervention	-	-
**Bohr 2000**	Traditional education	EDU	1-h education session (lecture, informational handouts and brief question and answer session) about topics such as muscle physiology, ideal postures, task analysis, recommended office equipment location, general wellness information related to exercise, nutrition, and smoking.	Not specified	One time intervention
	Participatory education	ERG	2-h active learning sessions incorporating discussions and problem solving exercises to aid in applying ergonomic concepts to the work environment; including, e.g., hands-on demonstration of workstation evaluation and modification, case studies, supervised evaluation and modification of work areas.	Instructor	One time intervention
	Control group	NI	No intervention	-	-

**Brakenridge 2018**	Organisational support	MCI (EDU, BI)	Information booklet, welcome email, five fortnightly emails, workplace health presentations to participants, discussions with team managers; key intervention message: " stand up, sit less, move more"; participants received emailed feedback on their sitting, standing, and stepping time as measured via activity monitor (baseline and three months), as well as group-level summaries at baseline.	"Workplace champion" (head of workplace wellbeing of the organisation)	3
	Organisational support + tracker	MCI (EDU, BI)	Organisational support like other group; additionally, participants received an activity tracker (worn as a belt), which measures behaviours like sitting and standing and provides feedback on these behaviours through a mobile app.	"Workplace champion" (head of workplace wellbeing of the organisation)	3
**Brisson 1999**	Ergonomic training program	ERG	Intervention aimed at adjusting the postural and visual components of the workstation correctly and organising work activities in a preventive manner; 2 group sessions of 3 hours (with the workers supervisors present) involving demonstrations, simulations, discussions, and lectures as well as self-diagnosis of the participant's workstation using a photograph.	Occupational health and safety professionals	0.5
	Reference group	NI	No intervention	-	-
**Coenen 2017**	Stand up Victoria	MCI (ERG, EDU, BI)	Key intervention message: "stand up, sit less, move more"; individual, organisational and environmental components including, e.g., adaptation of the workstations, written instructions regarding appropriate postures, senior management consultation, representatives' consultation workshops, participant information and brainstorming sessions, tailored e-mails, role modelling by "team champions" (trained worksite team leaders), one individual coaching session with subsequent telephone support.	Health coaches (with training in psychology), research staff	12
	Control group	NI	Written feedback on activity and biomarker outcomes measured at 3 months and 12 months.	-	-
**Conlon 2008**	Conventional mouse	NI	Participants received a conventional mouse; chair and workstation were adjusted for all participants; one unannounced visit to all participants to confirm compliance one month after beginning of the intervention.	-	1

	Alternative mouse	ERG	Like "conventional mouse" group, but participants received a mouse with a vertical handle for grasping, a flat base and a roller ball for tracking for the duration of the study.	-	12
	Conventional mouse + forearm support board	ERG	Like "conventional mouse" group, but participants also received a forearm support board (a large butterfly-shaped board that is attached to a desk) for the duration of the study.	-	12
	Alternative mouse plus forearm support board	ERG	Like "alternative mouse" group, but participants also received a forearm support board for the duration of the study.	-	12
**Dalager 2017**	Training group	PA	Individually tailored 1-h exercise training program performed once weekly at or near the workplace; the first year of training was fully supervised, during the second year, monthly supervision was provided; participants additionally were encouraged by "health ambassadors" (trained peers) to engage in physical activity.	Exercise training specialist (training in sports science)	24
	Control group	NI	No intervention	-	-
**Edwardson 2018**	Stand More At Work Intervention	MCI (ERG, EDU, BI)	Provision of height-adjustable workstations, educational seminar (30 min) on health consequences of sitting and benefits of reducing sitting, information leaflet, demonstration of desk to each participant, activity monitor feedback on sitting time, goal setting and action planning, provision of activity tracker, educational/motivational posters, coaching sessions every three months (15 mins; either face-to face or by telephone); participants received the results of health measures (e.g. weight, blood pressure) taken at each follow-up.	Research team	12
	Control group	NI	Participants received the results of health measures (e.g. weight, blood pressure) taken at each follow-up.	-	-
**Eklöf 2006**	Individual feedback	MCI (ERG, BI)	1-h individual feedback session for each participant about computer ergonomics and psychosocial factors; information included self-reported extent of computer work, self-reported physical complaints, comfort during computer work (with reference to workplace ergonomics), expert-assessed ergonomic standard of workplace design and working technique.	Physiotherapists specialised in ergonomics	One time intervention

	Supervisor feedback	MCI (ERG, BI)	1-h feedback session (content like first group) for the group supervisor alone.	Physiotherapists specialised in ergonomics	One time intervention
	Group feedback	MCI (ERG, BI)	1-h feedback session (content like first group) for the entire group with the supervisor present.	Physiotherapists specialised in ergonomics	One time intervention
	Control group	NI	No intervention	-	-
**Gerr 2005**	Alternate intervention	ERG	Reconfiguration of participants' workstations based on results from a prospective study; provision of verbal and written instructions; visits of study staff three days and one week after reconfiguration to check on continued maintenance of the posture and provide adjustments, if necessary.	Study staff	One week
	Conventional intervention	ERG	Like “alternate intervention”, but workstations were reconfigured based on available recommendations from several sources (e.g. US Department of Labor).	Study staff	One week
	No intervention	NI	No intervention	-	-
**Joines 2015**	Intervention group	ERG	Participants received an adjustable LED task light; overhead lighting was adjusted if necessary; instructions were provided on how to use the new light.	Not specified	6
	Control group	NI	No intervention	-	-
**Karatrantou 2020**	Training group	PA	15-20 min chair-based supervised workplace training program, twice per day (2-3 hours rest between sessions) in small groups; including flexibility, strength, balance and aerobic exercise.	Physical trainer	6
	Control group	NI	No intervention	-	-
**King 2013**	Biofeedback mouse	BI	Participants received a biofeedback mouse (the mouse gently vibrated if the worker’s hand had been idle on the mouse for more than 12 seconds; the feedback was a reminder to rest the arm in neutral postures when not in use); 1-h study information session with time for questions and answers; invitation to watch an online manufacturer video about the mouse.	Not specified	6
	Control group	NI	Participants received the same biofeedback mouse, but with the vibration mechanism turned off over the study duration; 1-h study information session with time for questions and answers.	Not specified	One time intervention

**Konradt 2020**	Sit-stand office desks	MCI (ERG, EDU)	Participants received sit-stand desks (fully adjustable in height) and were instructed on how to use them; introductory course in "healthy standing" as well as "healthy sitting".	Two trained psychologists	6
	Control group	NI	Introductory course in "healthy sitting" (basic information on the problems associated with sedentary behaviour and guidance on healthy and unhealthy sitting).	Two trained psychologists	One time intervention
**Lee 2020**	Ergonomic workstation intervention	ERG	The workstations (i.e. height of table, chair, monitor, position of keyboard and mouse) were adjusted based on ergonomic recommendations and based on individual anthropometric measurements.	Not specified	One time intervention
	Control group	NI	No intervention	-	-
**Mahmud 2010**	Office ergonomics training	MCI (ERG, EDU)	Lecture on office ergonomics (e.g. on musculoskeletal discomforts, ergonomics improvements and adjustments of workstations, importance of break and stretching exercises); trainers visited participants' workstations and provided assistance with adjusting workstations; information leaflet.	Trainers from the National Institute of Safety and Health	One time intervention
	Control group	NI	Participants received an information leaflet.	-	One time intervention
**Meijer 2009**	Computer mouse with feedback signal	BI	Participants received a computer mouse with feedback signal (if the mouse was held for over 12 seconds without active usage, the mouse provided a feedback signal to remind the user to take his or her hand from the mouse and to relax his or her hand and forearm); invitation to watch an instructional online video.	-	8
	Control group	NI	Participants used a mouse without feedback signal.	-	-
**Moore 2012**	Daily exercise	PA	Instruction on 6 calisthenic exercises in a total of 5 group sessions; the exercises were to be done once a day for 15 min; compliance checks (interviews with principal investigator) during the study period; "travel card" with drawings and exercise times for trips away from home.	Principal investigator	12
	Normal activity	NI	No intervention	-	-

**Pereira 2019**	Ergonomics and exercise training	MCIPA (ERG, PA)	30-45 min workstation ergonomics assessment; individualized ergonomic intervention (including, e.g., individual adjustments and workstation items) based on the assessment; individualized neck-specific exercise program, carried out in groups for 20 minutes, three times weekly; the first two exercise sessions were supervised.	Physiotherapist /occupational therapist	3
	Ergonomics and health promotion	MCI (ERG, EDU)	Workstation ergonomics assessment and individualized ergonomic intervention like the first group; one weekly 60-min health promotion session (discussing, e.g., stress and conflict management and healthy eating).	Physiotherapist / occupational therapist, health professional	3
**Proper 2003**	Individual counselling	MCI (BI, EDU)	Seven individual counselling sessions (20 minutes each) based on the individual's stage of behavioural change; counselling focused primarily on the enhancement of the individual's level of physical activity and secondarily on the promotion of healthy nutrition habits and other lifestyle factors; written information about lifestyle factors.	Trained physiotherapist	9
	Control group	NI	Participants received written information about lifestyle factors.	-	One time intervention
**Rempel 2006**	Ergonomic training	ERG	Ergonomic training (involving, e.g., recommendations on maintaining an erect posture while sitting, adjusting the workplace correctly, scheduled breaks); one unannounced visit after one month to ensure compliance with the intervention.	Trained research associate	1
	Ergonomic training + trackball	ERG	Ergonomic training like the first group; a trackball was installed next to the keyboard.	Trained research associate	12
	Ergonomic training + armboard	ERG	Ergonomic training like the first group; an armboard (wraparound, padded arm support that attaches to the top, front edge of the work surface) was installed.	Trained research associate	12
	Ergonomic training + trackball + armboard	ERG	Ergonomic training like the first group; a trackball and an armboard were installed.	Trained research associate	12

**Renaud 2020**	Dynamic work intervention	MCI (ERG, EEQ, BI)	Instalment of electrically adjustable sit-stand workstations, desk bikes and office sit balls, meetings between physiotherapist and managers of each participating department, 2 group sessions (30 min) with a physiotherapist (topics: e.g. risks associated with prolonged sitting, correct usage of the new furniture, overcoming barriers to decrease sitting time / increase standing or stepping time); at least 2 on-site consultations with the physiotherapist (for tips and answering questions), provision of a sitting tracker and a self-help booklet on sitting less and moving more; additionally all intervention components of the second group.	Occupational physiotherapists	8
	Usual practice	MCI (ERG, EEQ)	Promotion of walking meetings, using stairways and mapped (telephone) walking routes with footsteps on the floor, availability of lunch bags to take along on a lunch walk; shared (short-stay) work zones including sit-stand workstations and desk bikes at the entrance of company buildings.	-	8
**Speklé 2010**	Intervention group	MCIPA (EDU, BI, ERG, PA)	Participants received feedback on their exposure to risk factors and prevalence of arm, shoulder and neck symptoms and a risk profile was created; based on the risk profiles of employees, tailored intervention programmes were proposed to the organisation (multiple pre-defined interventions/intervention components were available; e.g. ergonomic advice, education, stress relaxation; interventions on individual or group level); organisations were responsible for carrying out the programmes.	Depending on the programme; e.g. occupational physicians, physiotherapists	Not specified
	Usual care	NI	Participants received general advice; workers with severe arm, shoulder and neck symptoms were invited by their occupational physician for a consultation.	In some instances: Occupational physician	Not specified

PA: Physical activity; MCIPA: Multicomponent intervention with physical activity; EDU: Education; BI: Behavioural intervention, EEQ: Exercise Equipment; MCI: Other multicomponent intervention; ERG: Ergonomics; NI: No/minimal intervention.

Supplementary figure S3a-c. Network graphs displaying overall risk of bias

a) Outcome participants with back pain

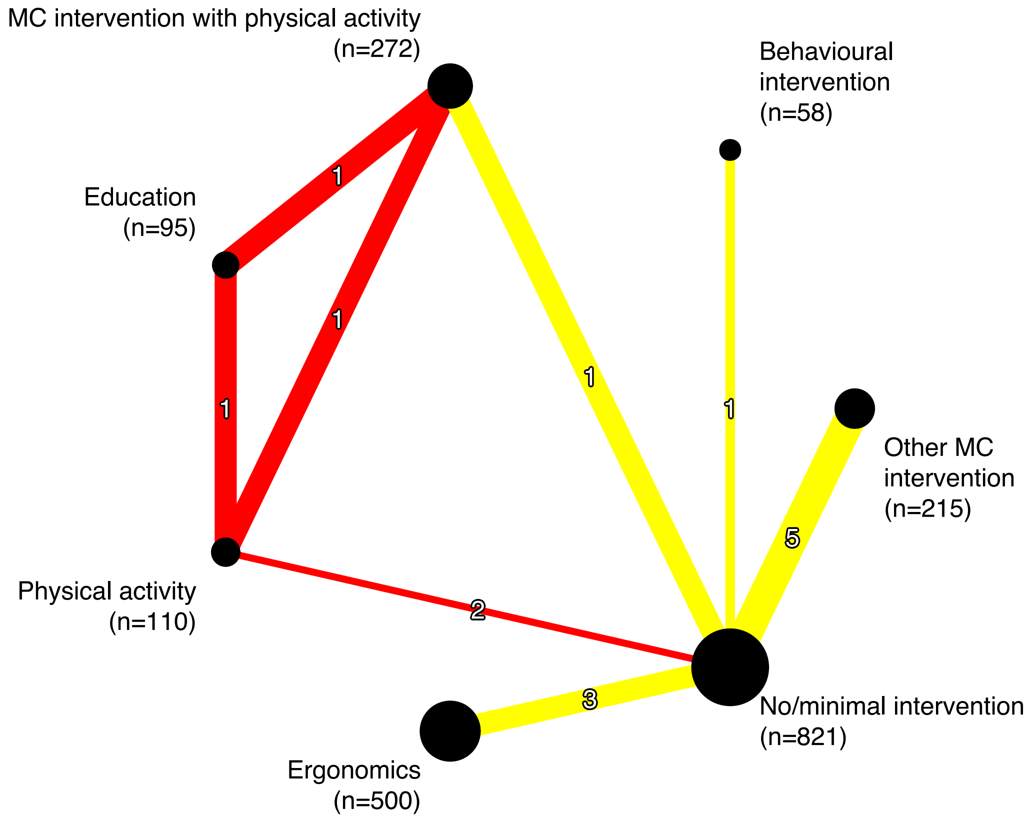

b) Outcome back pain intensity

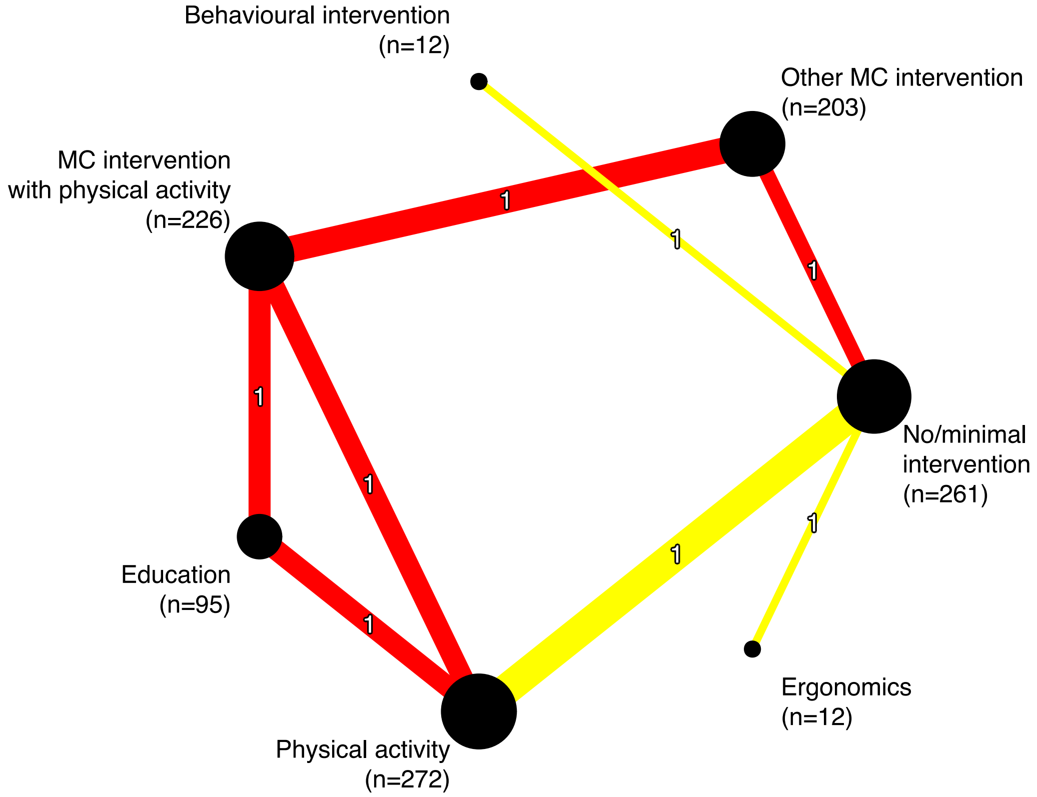

c) Outcome days of work absence

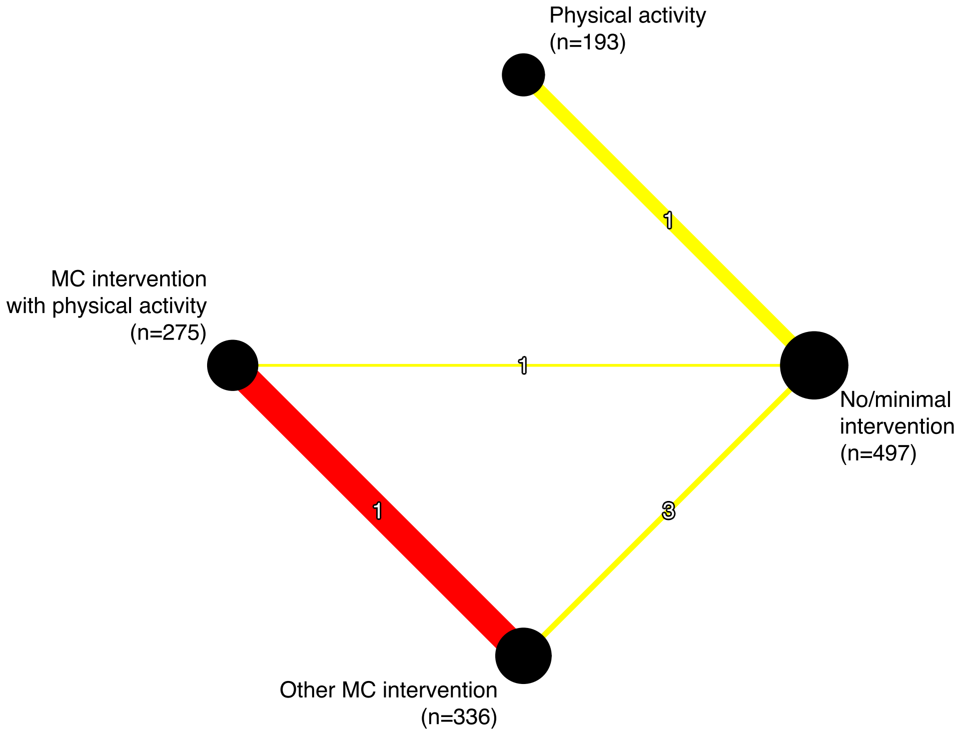

Red edges indicate high overall risk of bias (RoB) for the majority of studies forming the respective comparison, yellow edges indicate moderate overall RoB for the majority of studies. The size of each node is proportional to the total number of participants assigned to the respective intervention, the width of each line is proportional to the inverse of the standard error of the respective direct comparison, the numbers on the lines correspond to the numbers of studies contributing to the respective direct comparison.
MC: multicomponent.

Supplementary table S10a-c. GRADE evaluation for the network meta-analyses

a) Outcome participants with back pain

		Direct evidence		Indirect evidence		Network evidence	
Comparison	N studies	RR (95% CI)	Certainty of evidence	RR (95% CI)	Certainty of evidence	RR (95% CI)	Certainty of evidence
MC intervention with physical activity vs. physical activity	1	1.09 (0.73-1.65)	⊕⊕⊕◯[a]	1.91 (0.65-5.63)	⊕◯◯◯	1.17 (0.80-1.72)	⊕⊕◯◯[g]
MC intervention with physical activity vs. other MC intervention	0	-		0.84 (0.53-1.32)	⊕⊕⊕◯	0.84 (0.53-1.32)	⊕⊕◯◯[g]
MC intervention with physical activity vs. ergonomics	0	-		0.68 (0.40-1.17)	⊕⊕⊕◯	0.68 (0.40-1.17)	⊕⊕◯◯[g]
MC intervention with physical activity vs. behavioural intervention	0	-		0.71 (0.30-1.65)	⊕⊕⊕◯	0.71 (0.30-1.65)	⊕◯◯◯[h]
MC intervention with physical activity vs. education	1	0.98 (0.67-1.45)	⊕⊕⊕◯[a]	3.04 (0.31-30.12)	⊕⊕⊕◯	1.01 (0.69-1.49)	⊕⊕◯◯[g]
MC intervention with physical activity vs. no/minimal intervention	1	0.82 (0.55-1.22)	⊕⊕⊕◯[b]	0.47 (0.16-1.38)	⊕◯◯◯	0.76 (0.52-1.11)	⊕⊕◯◯[g]
Physical activity vs. other MC intervention	0	-		0.71 (0.41-1.25)	⊕◯◯◯	0.71 (0.41-1.25)	⊕◯◯◯[h]
Physical activity vs. ergonomics	0	-		0.58 (0.31-1.09)	⊕◯◯◯	0.58 (0.31-1.09)	⊕◯◯◯[h]
Physical activity vs. behavioural intervention	0	-		0.60 (0.24-1.49)	⊕◯◯◯	0.60 (0.24-1.49)	⊕◯◯◯[h]
Physical activity vs. education	1	0.90 (0.60-1.36)	⊕⊕⊕◯[a]	0.33 (0.04-2.52)	⊕⊕⊕◯	0.86 (0.58-1.30)	⊕⊕◯◯[g]
Physical activity vs. no/minimal intervention	2	0.43 (0.16-1.16)	⊕◯◯◯[c,d]	0.75 (0.42-1.32)	⊕⊕⊕◯	0.65 (0.40-1.07)	⊕⊕◯◯[g]
Other MC intervention vs. ergonomics	0	-		0.82 (0.52-1.30)	⊕⊕⊕◯	0.82 (0.52-1.30)	⊕⊕◯◯[g]
Other MC intervention vs. behavioural intervention	0	-		0.84 (0.38-1.89)	⊕⊕⊕◯	0.84 (0.38-1.89)	⊕◯◯◯[h]
Other MC intervention vs. education	0	-		1.21 (0.68-2.17)	⊕⊕⊕◯	1.21 (0.68-2.17)	⊕◯◯◯[h]
Other MC intervention vs. no/minimal intervention	5	0.91 (0.70-1.18)	⊕⊕⊕◯[e]	-		0.91 (0.70-1.18)	⊕⊕◯◯[g]
Ergonomics vs. behavioural intervention	0	-		1.03 (0.44-2.42)	⊕⊕⊕◯	1.03 (0.44-2.42)	⊕◯◯◯[h]
Ergonomics vs. education	0	-		1.48 (0.78-2.82)	⊕⊕⊕◯	1.48 (0.78-2.82)	⊕◯◯◯[h]
Ergonomics vs. no/minimal intervention	3	1.11 (0.76-1.63)	⊕⊕⊕◯[f]	-		1.11 (0.76-1.63)	⊕⊕◯◯[g]
Behavioural intervention vs. education	0	-		1.44 (0.57-3.61)	⊕⊕⊕◯	1.44 (0.57-3.61)	⊕◯◯◯[h]
Behavioural intervention vs. no/minimal intervention	1	1.08 (0.50-2.31)	⊕⊕⊕◯[b]	-		1.08 (0.50-2.31)	⊕◯◯◯[h]
Education vs. no/minimal intervention	0	-		0.75 (0.45-1.26)	⊕⊕⊕◯	0.75 (0.45-1.26)	⊕⊕◯◯[g]

RR: risk ratio; CI: confidence interval. ⊕⊕⊕⊕ high; ⊕⊕⊕◯ moderate; ⊕⊕◯◯ low; ⊕◯◯◯ very low.

[a]downgraded by one level for risk of bias (1/1 studies rated as “high”).
[b]downgraded by one level for risk of bias (1/1 studies rated as “some concerns”).
[c]downgraded by two levels for risk of bias (2/2 studies rated as “high”).
[d]downgraded by one level for inconsistency (I^2=54.7%; p=0.1375).
[e]downgraded by one level for risk of bias (3/5 studies rated as “some concerns”, 2/5 studies rated as “high”).
[f]downgraded by one level for risk of bias (2/3 studies rated as “some concerns”, 1/3 studies rated as “high”).
[g]downgraded by one level for imprecision due to wide 95% CI including a potential positive, null or negative effect.
[h]downgraded by two levels for imprecision due to very wide 95% CI including a potential positive, null or negative effect.

b) Outcome back pain intensity

		Direct evidence		Indirect evidence		Network evidence	
Comparison	N studies	SMD (95% CI)	Certainty of evidence	SMD (95% CI)	Certainty of evidence	SMD (95% CI)	Certainty of evidence
MC intervention with physical activity vs. Physical activity	1	-0.05 (-0.34-0.25)	⊕⊕⊕◯[a]	-0.25 (-0.77-0.26)	⊕⊕⊕◯	-0.10 (-0.36-0.16)	⊕⊕◯◯[d]
MC intervention with physical activity vs. Other MC intervention	1	-0.10 (-0.33-0.14)	⊕⊕◯◯[b]	0.11 (-0.44-0.65)	⊕⊕⊕◯	-0.06 (-0.28-0.15)	⊕⊕◯◯[d]
MC intervention with physical activity vs. Ergonomics	0	-		0.25 (-0.58-1.08)	⊕⊕⊕◯	0.25 (-0.58-1.08)	⊕◯◯◯[e]
MC intervention with physical activity vs. Behavioural intervention	0	-		-0.08 (-0.93-0.77)	⊕⊕⊕◯	-0.08 (-0.93-0.77)	⊕◯◯◯[f]
MC intervention with physical activity vs. Education	1	-0.12 (-0.40-0.16)	⊕⊕⊕◯[a]	-0.54 (-1.74-0.65)	⊕⊕⊕◯	-0.15 (-0.42-0.13)	⊕⊕◯◯[d]
MC intervention with physical activity vs. No minimal intervention	0	-		-0.23 (-0.52-0.05)	⊕⊕⊕◯	-0.23 (-0.52-0.05)	⊕⊕◯◯[g]
Physical activity vs. Other MC intervention	0	-		0.04 (-0.26-0.33)	⊕⊕⊕◯	0.04 (-0.26-0.33)	⊕⊕◯◯[h]
Physical activity vs. Ergonomics	0	-		0.35 (-0.46-1.15)	⊕⊕⊕◯	0.35 (-0.46-1.15)	⊕◯◯◯[i]
Physical activity vs. Behavioural intervention	0	-		0.02 (-0.80-0.85)	⊕⊕⊕◯	0.02 (-0.80-0.85)	⊕◯◯◯[j]
Physical activity vs. Education	1	-0.07 (-0.37-0.22)	⊕⊕⊕◯[a]	0.30 (-0.74-1.34)	⊕⊕⊕◯	-0.05 (-0.33-0.24)	⊕⊕◯◯[h]
Physical activity vs. No minimal intervention	1	-0.11 (-0.31-0.09)	⊕⊕⊕◯[c]	-0.31 (-0.87-0.25)	⊕⊕◯◯	-0.13 (-0.32-0.06)	⊕⊕◯◯[d]
Other MC intervention vs. Ergonomics	0	-		0.31 (-0.52-1.15)	⊕⊕⊕◯	0.31 (-0.52-1.15)	⊕◯◯◯[e]
Other MC intervention vs. Behavioural intervention	0	-		-0.01 (-0.87-0.84)	⊕⊕⊕◯	-0.01 (-0.87-0.84)	⊕◯◯◯[j]
Other MC intervention vs. Education	0	-		-0.08 (-0.41-0.25)	⊕⊕◯◯	-0.08 (-0.41-0.25)	⊕◯◯◯[h]
Other MC intervention vs. No minimal intervention	1	-0.26 (-0.67-0.15)	⊕⊕⊕◯[a]	-0.06 (-0.49-0.37)	⊕⊕◯◯	-0.17 (-0.46-0.13)	⊕⊕◯◯[d]
Ergonomics vs. Behavioural intervention	0	-		-0.33 (-1.45-0.79)	⊕⊕⊕◯	-0.33 (-1.45-0.79)	⊕◯◯◯[f]
Ergonomics vs. Education	0	-		-0.39 (-1.24-0.45)	⊕⊕⊕◯	-0.39 (-1.24-0.45)	⊕◯◯◯[k]
Ergonomics vs. No minimal intervention	1	-0.48 (-1.26-0.30)	⊕⊕⊕◯[c]			-0.48 (-1.26-0.30)	⊕◯◯◯[l]
Behavioural intervention vs. Education	0	-		-0.07 (-0.93-0.80)	⊕⊕⊕◯	-0.07 (-0.93-0.80)	⊕◯◯◯[j]
Behavioural intervention vs. No minimal intervention	1	-0.15 (-0.95-0.65)	⊕⊕⊕◯[c]			-0.15 (-0.95-0.65)	⊕◯◯◯[m]
Education vs. No minimal intervention	0	-		-0.09 (-0.41-0.24)	⊕⊕⊕◯	-0.09 (-0.41-0.24)	⊕⊕◯◯[h]

SMD: standardised mean difference; CI: confidence interval. ⊕⊕⊕⊕ high; ⊕⊕⊕◯ moderate; ⊕⊕◯◯ low; ⊕◯◯◯ very low.

[a]downgraded by one level for risk of bias (1/1 studies rated as "high").
[b]downgraded by two levels for risk of bias (1/1 studies rated as "high").
[c]downgraded by one level for risk of bias (1/1 studies rated as "some concerns").
[d]downgraded by one level for imprecision due to wide 95% CI including a potential small positive or null effect (2).
[e]downgraded by two levels for imprecision due to very wide 95% CI including a potential medium positive, null or large negative effect (2).
[f]downgraded by two levels for imprecision due to very wide 95% CI including a potential large positive, null or medium negative effect (2).
[g]downgraded by one level for imprecision due to wide 95% CI including a potential medium positive or null effect (2).
[h]downgraded by one level for imprecision due to wide 95% CI including a potential small positive, null or small negative effect (2).
[i]downgraded by two levels for imprecision due to very wide 95% CI including a potential small positive, null or large negative effect (2).
[j]downgraded by two levels for imprecision due to very wide 95% CI including a potential large positive, null or large negative effect (2).
[k]downgraded by two levels for imprecision due to very wide 95% CI including a potential large positive, null or small negative effect (2).
[l]downgraded by two levels for imprecision due to very wide 95% CI including a potential large positive, null or small negative effect (2) and very low number of participants (n=26).
[m]downgraded by two levels for imprecision due to very wide 95% CI including a potential large positive, null or medium negative effect (2) and very low number of participants (n=24).

c) Outcome days of work absence

	Direct evidence			Indirect evidence		Network evidence	
**Comparison**	**N studies**	**MD (95% CI)**	**Certainty of evidence**	**MD (95% CI)**	**Certainty of evidence**	**MD (95% CI)**	**Certainty of evidence**
MC intervention with physical activity vs. Physical activity	0	-		1.19 (-1.20-3.58)	⊕⊕⊕◯	1.19 (-1.20-3.58)	⊕⊕◯◯[d]
MC intervention with physical activity vs. Other MC intervention	1	-0.10 (-0.57-0.37)	⊕⊕◯◯[a]	-2.76 (-8.55-3.04)	⊕⊕⊕◯	-0.12 (-0.59-0.35)	⊕⊕⊕◯
MC intervention with physical activity vs. No minimal intervention	1	-2.12 (-7.42-3.18)	⊕⊕⊕◯[b]	0.54 (-1.86-2.94)	⊕⊕◯◯	0.09 (-2.10-2.27)	⊕◯◯◯[d]
Physical activity vs. Other MC intervention	0	-		-1.30 (-3.66-1.05)	⊕⊕⊕◯	-1.30 (-3.66-1.05)	⊕⊕◯◯[d]
Physical activity vs. No minimal intervention	1	-1.10 (-2.07- -0.13)	⊕⊕⊕◯[b]	-		-1.10 (-2.07- -0.13)	⊕⊕⊕◯
Other MC intervention vs. No minimal intervention	3	0.64 (-1.71-2.99)	⊕⊕⊕◯[c]	-2.02 (-7.34-3.30)	⊕⊕◯◯	0.20 (-1.95-2.35)	⊕◯◯◯[d]

MD: mean difference; CI: confidence interval. ⊕⊕⊕⊕ high; ⊕⊕⊕◯ moderate; ⊕⊕◯◯ low; ⊕◯◯◯ very low.

[a]downgraded by two levels for risk of bias (1/1 studies rated as "high").
[b]downgraded by one level for risk of bias (1/1 studies rated as "some concerns").
[c]downgraded by one level for of bias (3/3 studies rated as "some concerns").
[d]downgraded by one level for imprecision due to wide 95% CI including a potential positive, null or negative effect.

Supplementary figure S4a-b. Forest plots for the network meta-analysis – outcome participants with back pain

a) Comparisons with available direct evidence

Comparison	Number of Studies	Direct Evidence	RR	95%-CI
**Behavioural intervention vs No/minimal intervention**				
Direct estimate	1	1.00	1.08	(0.50-2.31)
Indirect estimate				
Network estimate			1.08	(0.50-2.31)
**Education vs MC intervention with physical activity**				
Direct estimate	1	0.97	1.02	(0.69-1.50)
Indirect estimate			0.33	(0.03-3.26)
Network estimate			0.99	(0.67-1.44)
**Education vs Physical activity**				
Direct estimate	1	0.96	1.11	(0.74-1.68)
Indirect estimate			3.05	(0.40-23.45)
Network estimate			1.16	(0.77-1.73)
**Ergonomics vs No/minimal intervention**				
Direct estimate	3	1.00	1.11	(0.76-1.63)
Indirect estimate				
Network estimate			1.11	(0.76-1.63)
**MC intervention with physical activity vs No/minimal intervention**				
Direct estimate	1	0.88	0.82	(0.55-1.22)
Indirect estimate			0.47	(0.16-1.38)
Network estimate			0.76	(0.52-1.11)
**MC intervention with physical activity vs Physical activity**				
Direct estimate	1	0.87	1.09	(0.73-1.65)
Indirect estimate			1.91	(0.65-5.63)
Network estimate			1.17	(0.80-1.72)
**Other MC intervention vs No/minimal intervention**				
Direct estimate	5	1.00	0.91	(0.70-1.18)
Indirect estimate				
Network estimate			0.91	(0.70-1.18)
**Physical activity vs No/minimal intervention**				
Direct estimate	2	0.25	0.43	(0.16-1.16)
Indirect estimate			0.75	(0.42-1.32)
Network estimate			0.65	(0.40-1.07)

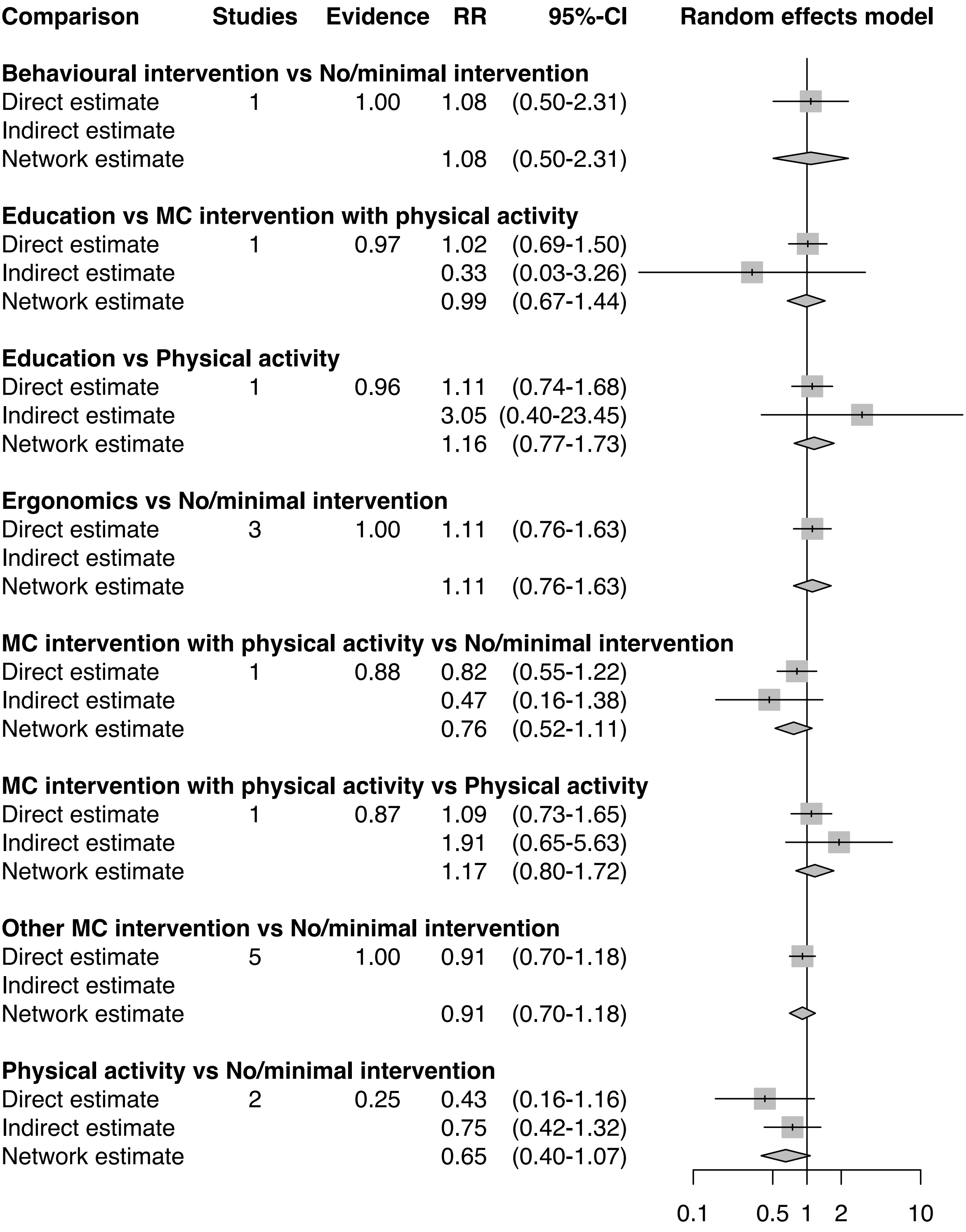

b) Active interventions versus no/minimal intervention

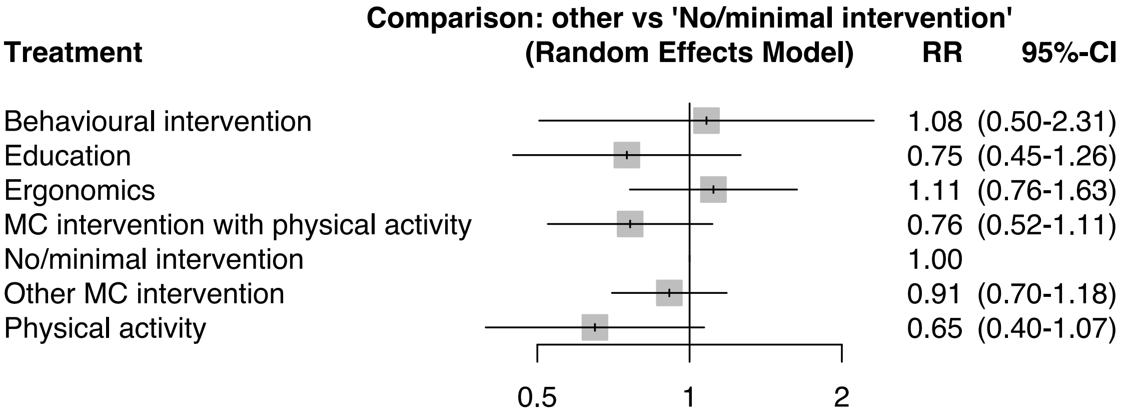

RR: risk ratio (RR<1 is beneficial); CI: confidence interval; MC: multicomponent.

Assessment of heterogeneity / inconsistency:

I^2=15.8%; Q (df=8)=9.5, p=0.3019; design-by-treatment interaction random effects model: Q (df=1)=0.89, p=0.3448; node-splitting-approach (for comparisons including both direct and indirect evidence):

Comparison (direct evidence proportion)	Ratio of ratios (direct versus indirect)	Z-value for disagreement (direct versus indirect)	p-value
Education vs MC intervention with physical activity (0.97)	3.09	0.95	0.3417
Education vs Physical activity (0.96)	0.36	-0.95	0.3417
MC intervention with physical activity vs No/minimal intervention (0.88)	1.75	0.95	0.3417
MC intervention with physical activity vs Physical activity (0.87)	0.57	-0.95	0.3417
Physical activity vs No/minimal intervention (0.25)	0.57	-0.95	0.3417

Supplementary table S11. P-scores for the network meta-analysis – outcome participants with back pain

Physical activity	0.8678
MC intervention with physical activity	0.6805
Education	0.6731
Other MC intervention	0.4694
Behavioural intervention	0.3099
No/minimal intervention	0.2998
Ergonomics	0.1996

Higher scores indicate greater benefit; MC: multicomponent.

Supplementary table S12. Results of the component meta-analysis (additive model) – outcome participants with back pain

Results for combinations of intervention components

Intervention components	RR (95%-CI)	p-value
Behavioural intervention	1.26 (0.84-1.89)	0.2631
Behavioural intervention + Education	1.01 (0.80-1.29)	0.9175
Education	0.80 (0.56-1.15)	0.2263
Education + Behavioural intervention + Ergonomics	1.04 (0.80-1.36)	0.7697
Education + Behavioural intervention + Ergonomics + Physical activity	0.76 (0.58-1.00)	0.0525
Education + Behavioural intervention + Exercise equipment + Physical activity	0.79 (0.53-1.20)	0.2756
Ergonomics	1.03 (0.79-1.34)	0.8433
Ergonomics + Education	0.83 (0.60-1.13)	0.2313
Ergonomics + Education + Behavioural intervention	1.04 (0.80-1.36)	0.7697
No/minimal intervention	1.00 (1.00-1.00)	-
Physical activity	0.73 (0.53-1.02)	0.0665

Results for intervention components

Intervention components	RR (95%-CI)	p-value
Behavioural intervention	1.26 (0.84-1.89)	0.2631
Education	0.80 (0.56-1.15)	0.2263
Ergonomics	1.03 (0.79-1.34)	0.8433
Exercise equipment	1.07 (0.74-1.55)	0.7152
Physical activity	0.73 (0.53-1.02)	0.0665

RR: risk ratio (RR<1 is beneficial); CI: confidence interval.
Heterogeneity/inconsistency: I^2=4%; Q (df=9)=9.38, p=0.4033.

Supplementary figure S5. Network graph for the component meta-analysis – outcome participants with back pain

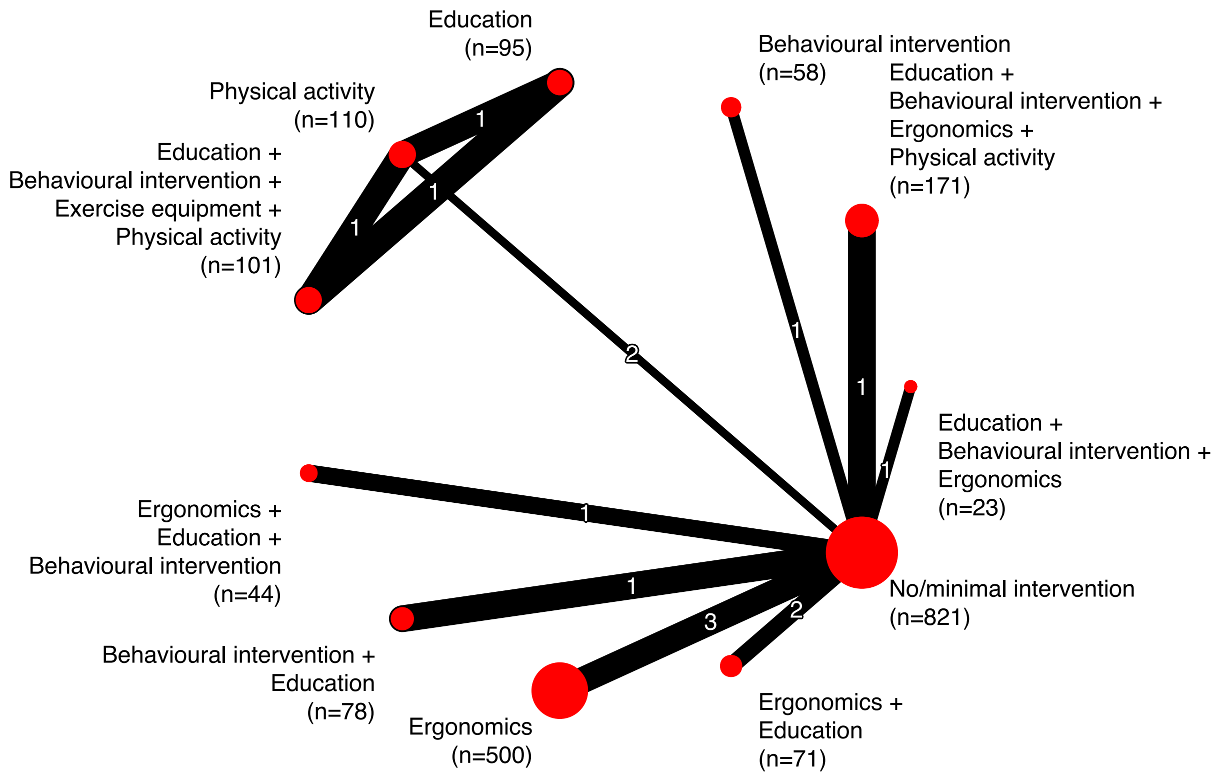

The size of each node is proportional to the total number of participants assigned to the respective intervention, the width of each line is proportional to the inverse of the standard error of the respective direct comparison, the numbers on the lines correspond to the numbers of studies contributing to the respective direct comparison.

Supplementary table S13. Description of results for studies not included in quantitative synthesis

Outcome	Study	Description of results
**Participants with back pain**	Bohr 2000	Statistically significant difference in total body pain/discomfort score (F(2;151)=3.16; p <.05) between intervention groups ("ergonomics", "education", "no/minimal intervention") over the course of the 12 month follow-up period. The score was calculated from questionnaire responses on whether pain/discomfort had been experienced during the last week in nine regions.
	Brakenridge 2018	No outcome data for follow-up ≥ 24 weeks available.
	Eklöf 2006	No statistically significant between groups ("other multicomponent intervention", "no/minimal intervention") in musculoskeletal symptoms or eye discomfort at 6 month follow-up (no estimates for between-group differences reported).
	Rempel 2006	Cox proportional hazard models revealed a statistically significant protective effect of the armboard for neck/shoulder disorders (HR=0.49; 95%-CI 0.24-0.97) at 12 month follow-up (four different "ergonomics" interventions).
	Renaud 2020	No statistically significant differences between groups (two different "other multicomponent interventions") in upper back, neck, shoulder complaints (OR=0.61; 95%-CI 0.19-3.11) and lower back complaints (OR=0.53; 95%-CI 0.19-1.43) at 8 month follow-up.
**Back pain intensity**	Brakenridge 2018	No outcome data for follow-up ≥24 weeks available.
	Conlon 2008	Linear regression analysis revealed no statistically significant difference in neck/shoulder discomfort score associated with the forearm support board (Beta=-0.02; 95%-CI -0.36-0.32; "no/minimal intervention"; three different "ergonomics" interventions). Discomfort scores were calculated cumulatively based on weekly survey results for the 12 month follow-up period.
	Joines 2015	Only data for one of the intervention groups ("ergonomics") reported, data for "no/minimal intervention" not reported.
	Rempel 2006	Linear regression analysis revealed a statistically significant reduction in neck/shoulder pain scores associated with the armboard (Beta=-0.48; 95%-CI -0.85- -0.10; four different "ergonomics" interventions). Pain scores were calculated cumulatively based on weekly survey results for the 12 month follow-up period.
	Renaud 2020	No outcome data reported.
**Participants absent from work**	Renaud 2020	No statistically significant differences between groups (two different "other multicomponent interventions") in sickness absenteeism (OR=0.61; 95%-CI 0.29-1.29) at 8 month follow-up.

OR: odds ratio; CI: confidence interval; HR: hazard ratio.

Supplementary figure S6a-b. Forest plots for the network meta-analysis – outcome back pain intensity

a) Comparisons with available direct evidence

Comparison	Number of Studies	Direct Evidence	SMD	95%-CI
**Behavioural intervention vs No/minimal intervention**				
Direct estimate	1	1.00	-0.15	(-0.95-0.65)
Indirect estimate				
Network estimate			-0.15	(-0.95-0.65)
**Education vs MC intervention with physical activity**				
Direct estimate	1	0.95	0.12	(-0.16-0.40)
Indirect estimate			0.54	(-0.65-1.74)
Network estimate			0.15	(-0.13-0.42)
**Education vs Physical activity**				
Direct estimate	1	0.92	0.07	(-0.22-0.37)
Indirect estimate			-0.30	(-1.34-0.74)
Network estimate			0.05	(-0.24-0.33)
**Ergonomics vs No/minimal intervention**				
Direct estimate	1	1.00	-0.48	(-1.26-0.30)
Indirect estimate				
Network estimate			-0.48	(-1.26-0.30)
**MC intervention with physical activity vs Other MC intervention**				
Direct estimate	1	0.84	-0.10	(-0.33-0.14)
Indirect estimate			0.11	(-0.44-0.65)
Network estimate			-0.06	(-0.28-0.15)
**MC intervention with physical activity vs Physical activity**				
Direct estimate	1	0.75	-0.05	(-0.34-0.25)
Indirect estimate			-0.25	(-0.77-0.26)
Network estimate			-0.10	(-0.36-0.16)
**Other MC intervention vs No/minimal intervention**				
Direct estimate	1	0.52	-0.26	(-0.67-0.15)
Indirect estimate			-0.06	(-0.49-0.37)
Network estimate			-0.17	(-0.46-0.13)
**Physical activity vs No/minimal intervention**				
Direct estimate	1	0.89	-0.11	(-0.31-0.09)
Indirect estimate			-0.31	(-0.87-0.25)
Network estimate			-0.13	(-0.32-0.06)

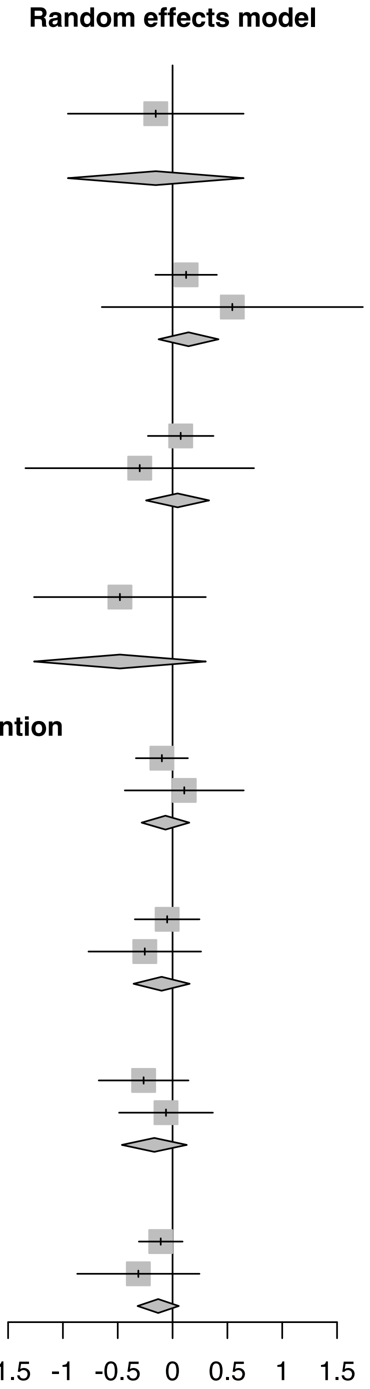

b) Active interventions versus no/minimal intervention

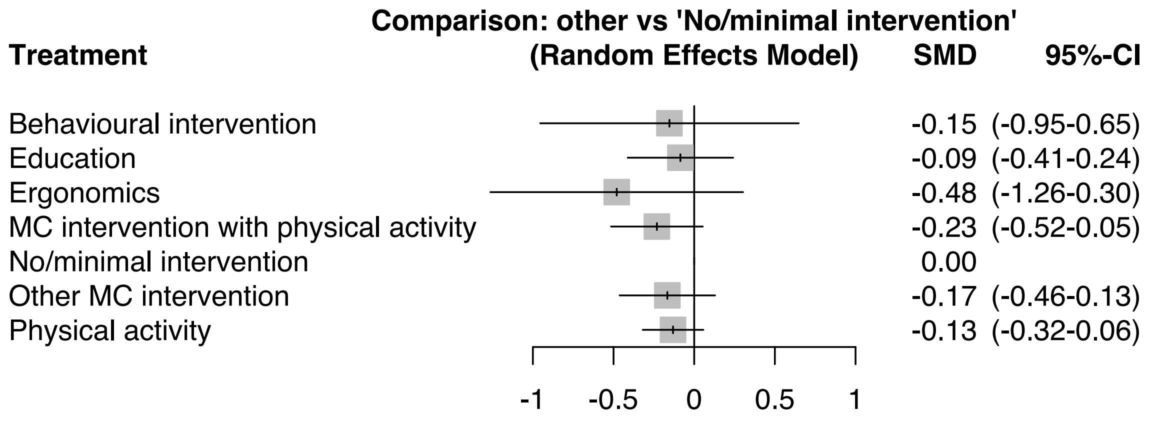

SMD: standardised mean difference (negative values are beneficial); CI: confidence interval; MC: multicomponent.

Assessment of heterogeneity / inconsistency

I^2=0%; Q (df=1)=0.46, p=0.4994; design-by-treatment interaction random effects model: Q (df=1)=0.46, p=0.4994; node-splitting-approach (for comparisons including both direct and indirect evidence):

Comparison (direct evidence proportion)	Difference direct and indirect estimates	Z-value for disagreement (direct versus indirect)	p-value
Education vs MC intervention with physical activity (0.95)	-0.42	-0.68	0.4994
Education vs Physical activity (0.92)	0.37	0.68	0.4994
MC intervention with physical activity vs Other MC intervention (0.84)	-0.20	-0.68	0.4994
MC intervention with physical activity vs Physical activity (0.75)	0.20	0.68	0.4994
Other MC intervention vs No/minimal intervention (0.52)	-0.20	-0.68	0.4994
Physical activity vs No/minimal intervention (0.89)	0.20	0.68	0.4994

Supplementary table S14. P-scores for the network meta-analysis – outcome back pain intensity

Ergonomics	0.7853
MC intervention with physical activity	0.6909
Other MC intervention	0.5277
Behavioural intervention	0.4882
Physical activity	0.4736
Education	0.3596
No/minimal intervention	0.1747

Higher scores indicate greater benefit; MC: multicomponent

Supplementary table S15. Results of the component meta-analysis (additive model) – outcome back pain intensity

Results for combinations of intervention components

**Intervention components**	**SMD (95%-CI)**	**p-value**
Behavioural intervention	-0.15 (-0.95-0.65)	0.7074
Education	0.00 (-0.26-0.26)	0.9951
Education + Behavioural intervention + Exercise equipment + Physical activity	-0.13 (-0.46-0.19)	0.4224
Ergonomics	-0.31 (-0.73-0.11)	0.1432
Ergonomics + Education	-0.31 (-0.68-0.06)	0.0976
Ergonomics + Physical activity	-0.41 (-0.80- -0.02)	0.0417
No/minimal intervention	0.00 (0.00-0.00)	-
Physical activity	-0.10 (-0.29-0.10)	0.3283

Results for intervention components

**Intervention components**	**SMD (95%-CI)**	**p-value**
Behavioural intervention	-0.15 (-0.95-0.65)	0.7074
Education	0.00 (-0.26-0.26)	0.9951
Ergonomics	-0.31 (-0.73-0.11)	0.1432
Exercise equipment	0.12 (-0.75-0.98)	0.7918
Physical activity	-0.10 (-0.29-0.10)	0.3283

SMD: standardised mean difference (negative values are beneficial); CI: confidence interval.
Heterogeneity/inconsistency: I^2=0%; Q (df=2)=0.26, p=0.8763.

Supplementary figure S7. Network graph for the component meta-analysis – outcome back pain intensity

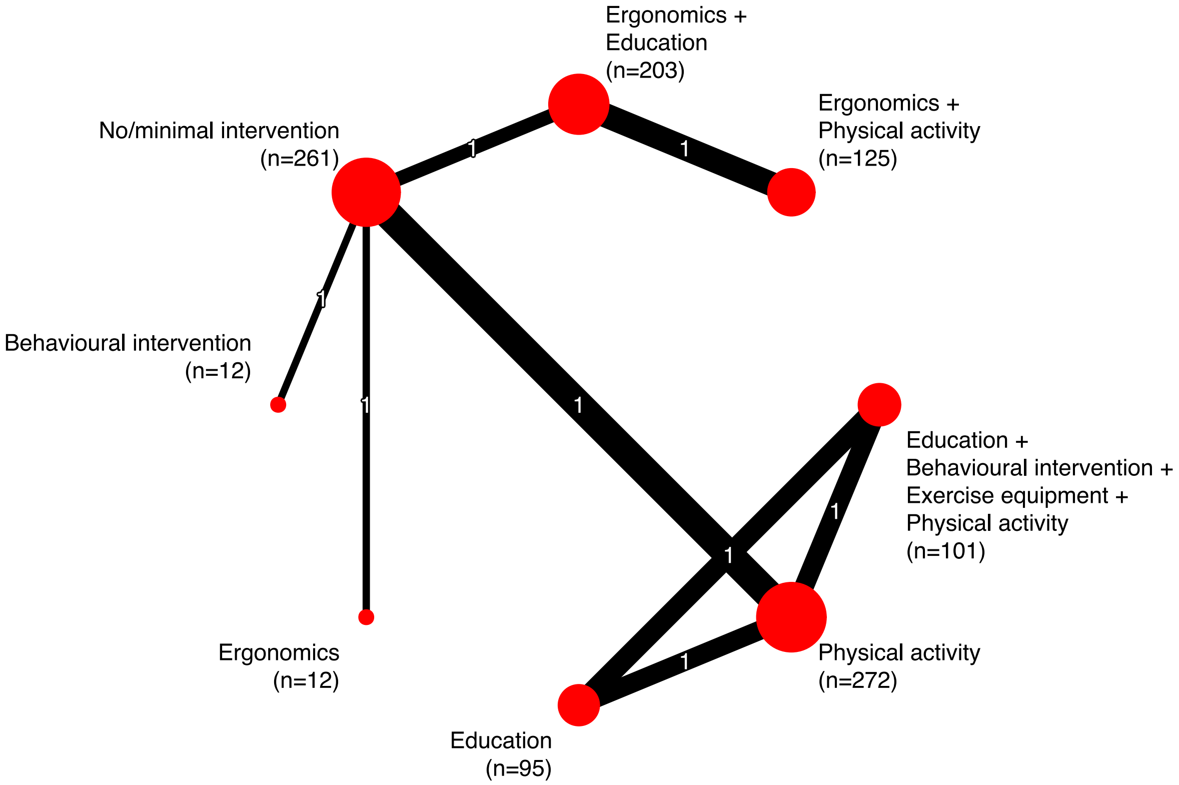

The size of each node is proportional to the total number of participants assigned to the respective intervention, the width of each line is proportional to the inverse of the standard error of the respective direct comparison, the numbers on the lines correspond to the numbers of studies contributing to the respective direct comparison.

Supplementary figure S8a-b. Forest plots for the network meta-analysis – outcome days of work absence

a) Comparisons with available direct evidence

Comparison	Number of Studies	Direct Evidence	MD	95%-CI
**MC intervention with physical activity vs No/minimal intervention**				
Direct estimate	1	0.17	-2.12	(-7.42-3.18)
Indirect estimate			0.54	(-1.86-2.94)
Network estimate			0.09	(-2.10-2.27)
**MC intervention with physical activity vs Other MC intervention**				
Direct estimate	1	0.99	-0.10	(-0.57-0.37)
Indirect estimate			-2.76	(-8.55-3.04)
Network estimate			-0.12	(-0.59-0.35)
**Other MC intervention vs No/minimal intervention**				
Direct estimate	3	0.84	0.64	(-1.71-2.99)
Indirect estimate			-2.02	(-7.34-3.30)
Network estimate			0.20	(-1.95-2.35)
**Physical activity vs No/minimal intervention**				
Direct estimate	1	1.00	-1.10	(-2.07- -0.13)
Indirect estimate				
Network estimate			-1.10	(-2.07- -0.13)

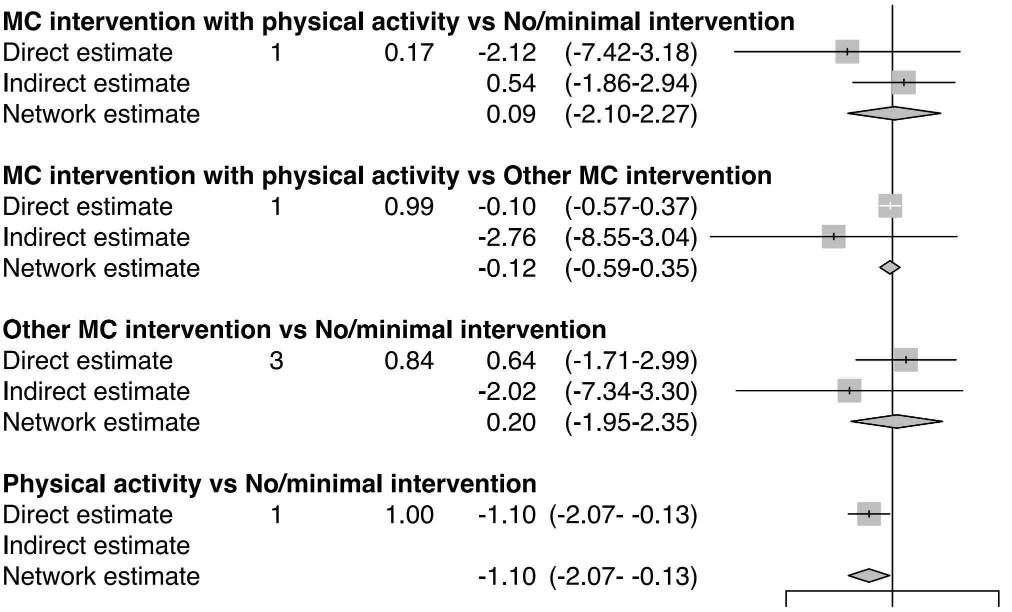

b) Active interventions versus no/minimal intervention

Treatment	MD	95%-CI
MC intervention with physical activity	0.09	(-2.10-2.27)
No/minimal intervention	0.00	
Other MC intervention	0.20	(-1.95-2.35)
Physical activity	-1.10	(-2.07- -0.13)

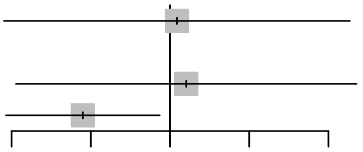

MD: mean difference (negative values are beneficial); CI: confidence interval; MC: multicomponent.

Assessment of heterogeneity / inconsistency:

I^2=0%; Q (df=3)=1.61, p=0.6567; design-by-treatment interaction random effects model: Q (df=1)=0.80, p=0.3700; node-splitting-approach (for comparisons including both direct and indirect evidence):

Comparison (direct evidence proportion)	Difference direct and indirect estimates	Z-value for disagreement (direct versus indirect)	p-value
MC intervention with physical activity vs No/minimal intervention (0.17)	-2.66	-0.90	0.3700
MC intervention with physical activity vs Other MC intervention (0.99)	2.66	0.90	0.3700
Other MC intervention vs No/minimal intervention (0.84)	2.66	0.90	0.3700

Supplementary table S16. P-scores for the network meta-analysis – outcome days of work absence

Physical activity	0.8942
MC intervention with physical activity	0.4404
No/minimal intervention	0.3728
Other MC intervention	0.2926

Higher scores indicate greater benefit; MC: multicomponent.

Supplementary table S17. Results of the component meta-analysis (additive model) - outcome days of work absence

Results for combinations of intervention components

Intervention components	MD (95%-CI)	p-value
Education + Behavioural intervention + Ergonomics + Physical activity	-0.17 (-3.35-3.01)	0.9159
Ergonomics + Education	-0.18 (-3.14-2.78)	0.9053
Ergonomics + Education + Behavioural intervention	0.99 (-2.14-4.13)	0.5344
Ergonomics + Physical activity	-0.28 (-3.28-2.72)	0.8550
No/minimal intervention	0.00 (0.00-0.00)	-
Physical activity	-1.17 (-2.13- -0.21)	0.0174

Results for intervention components

Intervention components	MD (95%-CI)	p-value
Behavioural intervention	1.17 (-3.14-5.49)	0.5939
Education	-1.07 (-2.14-0.01)	0.0512
Ergonomics	0.89 (-2.27-4.04)	0.5819
Physical activity	-1.17 (-2.13- -0.21)	0.0174

MD: mean difference (negative values are beneficial); CI: confidence interval.
Heterogeneity/inconsistency: I^2=0%; Q (df=2)=0.83, p=0.6603.

Supplementary figure S9. Network graph for the component network meta-analysis – outcome days of work absence

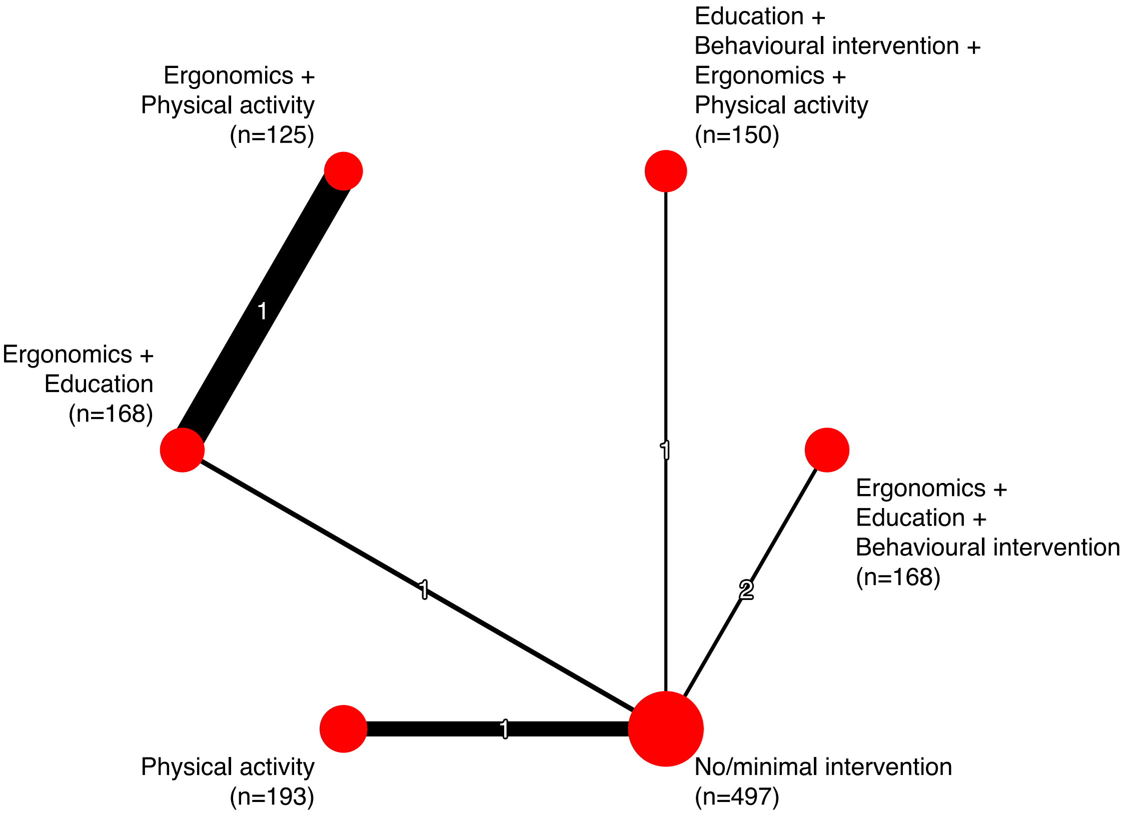

The size of each node is proportional to the total number of participants assigned to the respective intervention, the width of each line is proportional to the inverse of the standard error of the respective direct comparison, the numbers on the lines correspond to the numbers of studies contributing to the respective direct comparison.

Supplementary table S18. Intervention satisfaction

Study	Outcome	n assessed	Assessment	Intervention group	Follow-ups (months)	Results
**Brakenridge 2018[a]**	Acceptability	33 (quest.), 27 (interviews)	Questionnaire with questions regarding perceived usefulness, comfortability and ease of set-up, navigation/use and calibration of the LUMOback activity tracker; semi-structured telephone interviews	Organisational support + Tracker	3 (quest.), 6-10 (interviews)	About two thirds of participants rated the activity tracker as (somewhat) comfortable, while one third found it not at all comfortable. Most participants perceived set-up, navigation/use and calibration as easy and the features of the LUMOback at least somewhat useful.
**Coenen 2017[a]**	Acceptability	21 (interviews), 7 (focus groups)	Semi-structured interviews and 2 focus groups evaluating participants' perspectives on the intervention	Stand Up Victoria	12	Participants' overall experience of the intervention was very positive indicating acceptability of the intervention. Participants perceived that the increased workplace standing time had had positive impacts, e.g. on alertness, concentration and energy.
**Edwardson 2018[a]**	Feedback on intervention components	58 at 6 months, 55 (quest.) and 29 (focus groups) at 12 months	Questionnaire and 7 focus groups evaluating the different intervention components (e.g. height-adjustable workstations, educational seminar)	Stand More At Work Intervention	6 and 12 (quest.), 12 (focus groups)	Participants had positive attitudes towards the height-adjustable workstation, the educational seminar, feedback on sitting time and coaching sessions. The Darma cushion and action planning/goal setting diary were perceived as less helpful.
**Joines 2015**	Usability	48	Questionnaire with 9 items assessing usability, usefulness and desirability on a 6 point Likert scale	Adjustable light	6	Assessments of the light's usability, usefulness and desirability were positive. The participants reported benefiting from the use of the task light and indicated they would like the light in their workspace.

**Karatrantou 2020**	Enjoyment	n.r.	Quest. with 4 items on enjoyment using a 5-point Likert scale	Training group	6	Almost all participants (94.4%) reported high levels of enjoyment.
**King 2013**	Satisfaction	11	Quest. on satisfaction / willingness to continue using the biofeedback mouse in the future.	Biofeedback Mouse	6	About half of the participants (6 of 11) was willing to continue using the mouse in the future. Those not satisfied reported a general dislike for the mouse.
**Pereira 2019**[a]	Overall program satisfaction	n.r.	Quest. on overall program satisfaction and what participants liked and did not like about the study	EET, EHP	3	EET: Several participants noted that the intervention had changed their thinking about exercises, and that they would be more likely to think positively about similar exercises in the future. Some participants could see improvements which motivated them to continue attending the intervention. Several participants found it difficult to fit participation into their work day, or were uncomfortable performing the neck exercises. No results reported for EHP.
**Rempel 2006**	Subjective ratings of the intervention	46 (ET, ETA), 45 (ETT, ETTA)	Exit quest. to identify the reason for dropout and the participant's subjective ratings of the intervention	ET, ETT, ETA, ETTA	12 or at time of dropout	About half of the participants in ETT, ETA and ETTA and slightly less than half of the participants in the ET group liked their intervention or considered it helpful. Very few participants indicated that they did not like their intervention (all groups), some participants in the ETT and ETTA groups found intervention components difficult to use.

[a] data reported in Brakenridge et al (3), Hadgraft et al (4), Biddle et al (5), Welch et al (6);
n.r.: not reported; EET: Ergonomics and exercise training; EHP: Ergonomics and health promotion; ET: Ergonomic training only; ETT: Ergonomic training + trackball; ETA: Ergonomic training + armboard; ETTA: Ergonomic training + trackball + armboard.

Supplementary figure S10a-c. Forest plots for the sensitivity analyses excluding studies with high risk of bias – active interventions versus no/minimal intervention

a) Outcome participants with back pain

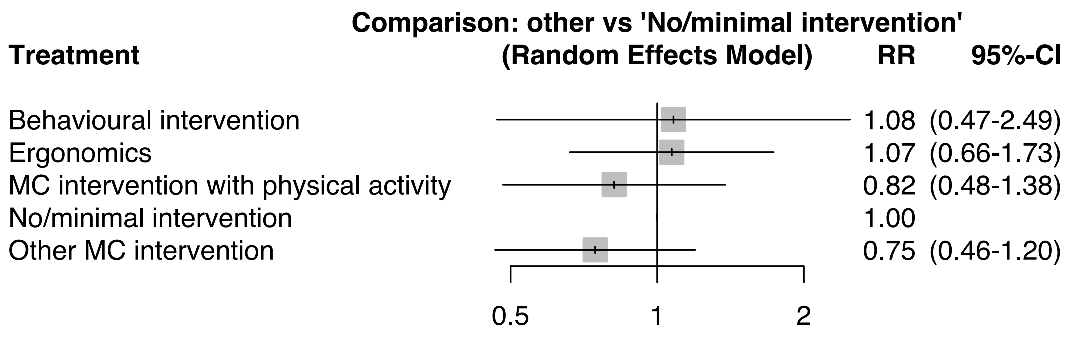

Number of studies: 7, number of pairwise comparisons: 7; RR: risk ratio (RR<1 is beneficial); CI: confidence interval; MC: multicomponent. Heterogeneity/inconsistency: I^2=27.7%; Q (df=3)=4.15, p=0.2457.

b) Outcome back pain intensity

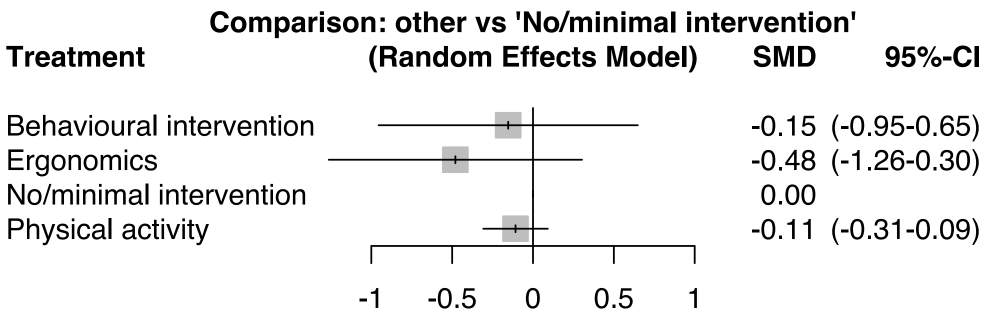

Number of studies: 3, number of pairwise comparisons: 3; SMD: standardised mean difference (negative values are beneficial); CI: confidence interval. Heterogeneity/inconsistency: I^2, Q: not applicable.

c) Outcome days of work absence

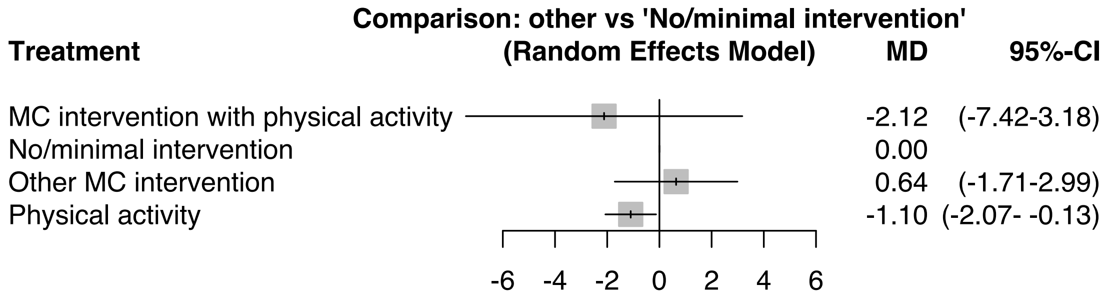

Number of studies: 5, number of pairwise comparisons: 5; MD: mean difference (negative values are beneficial); CI: confidence interval; MC: multicomponent. Heterogeneity/inconsistency: I^2=0%; Q (df=2)=0.81, p=0.6677.

Supplementary table S19a-c. P-scores for the sensitivity analyses excluding studies with high risk of bias

a) Outcome participants with back pain

Other MC intervention	0.7786
MC intervention with physical activity	0.6655
No/minimal intervention	0.3795
Behavioural intervention	0.3588
Ergonomics	0.3175

b) Outcome back pain intensity

Ergonomics	0.8060
Physical activity	0.4988
Behavioural intervention	0.4910
No/minimal intervention	0.2042

c) Outcome days of work absence

MC intervention with physical activity	0.7510
Physical activity	0.7507
No/minimal intervention	0.3109
Other MC intervention	0.1874

Higher scores indicate greater benefit; MC: multicomponent.

Supplementary figure S11a-g. Forest plots for the additional network meta-analyses for different localisations of back pain – active versus no/minimal intervention

a) Outcome participants with lower back pain

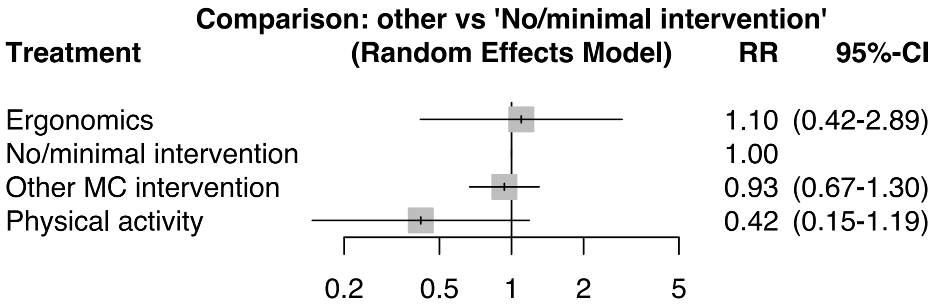

Number of studies: 7, number of pairwise comparisons: 7; RR: risk ratio (RR<1 is beneficial); CI: confidence interval; MC: multicomponent. Heterogeneity/inconsistency: I^2=36.1%; Q (df=4)=6.26, p=0.1805.

b) Outcome participants with neck (-shoulder) pain

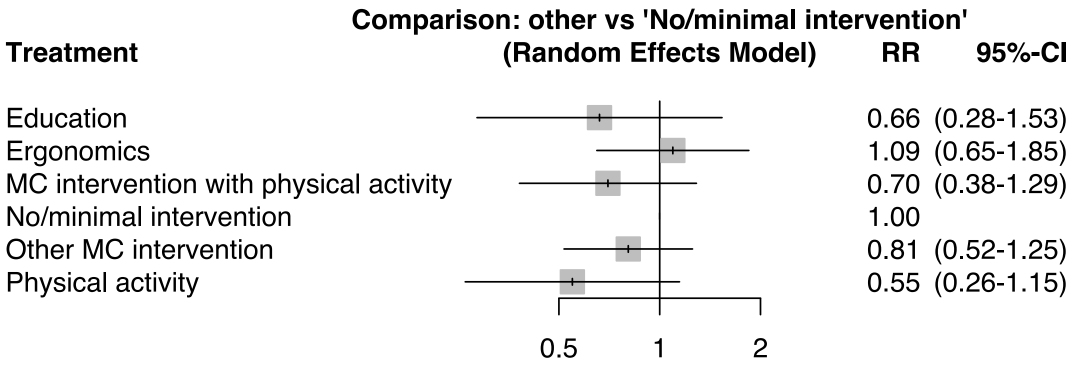

Number of studies: 10, number of pairwise comparisons: 12; RR: risk ratio (RR<1 is beneficial); CI: confidence interval; MC: multicomponent. Heterogeneity/inconsistency: I^2=49.4%; Q (df=6)=11.86, p=0.0651.

c) Outcome participants with upper back pain

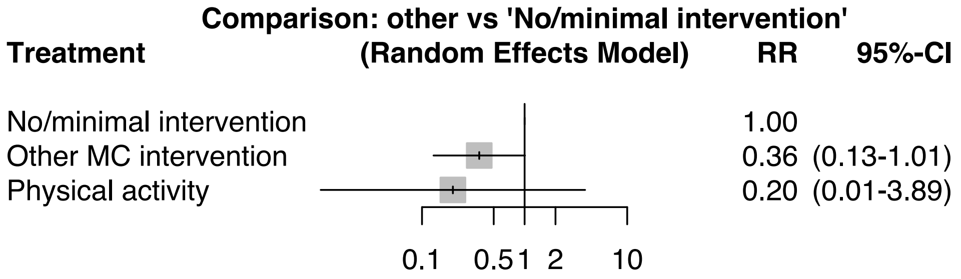

Number of studies: 2, number of pairwise comparisons: 2; RR: risk ratio (RR<1 is beneficial); CI: confidence interval; MC: multicomponent. Heterogeneity/inconsistency: I^2, Q: not applicable.

d) Outcome lower back pain intensity

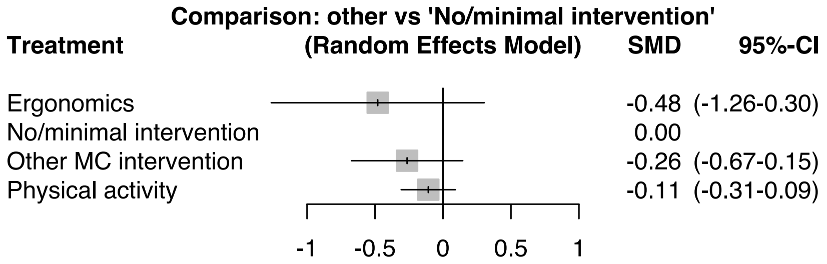

Number of studies: 3, number of pairwise comparisons: 3; SMD: standardised mean difference (negative values are beneficial); CI: confidence interval; MC: multicomponent. Heterogeneity/inconsistency: I^2, Q: not applicable.

e) Outcome neck (-shoulder) pain intensity

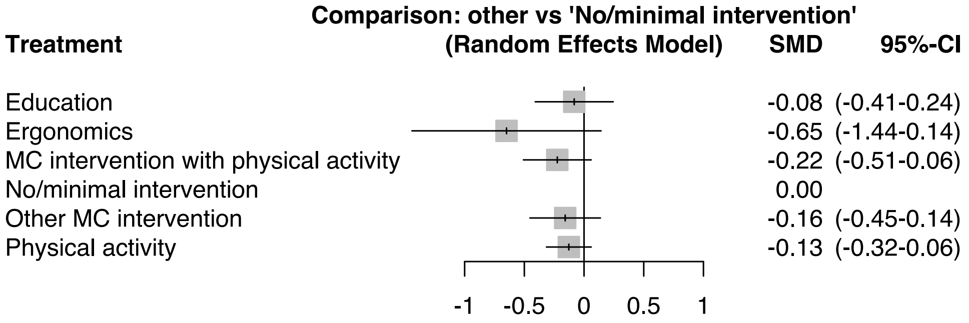

Number of studies: 5, number of pairwise comparisons: 7; SMD: standardised mean difference (negative values are beneficial); CI: confidence interval; MC: multicomponent. Heterogeneity/inconsistency: I^2=0%; Q (df=1)=0.42, p=0.5181.

f) Outcome upper back pain intensity

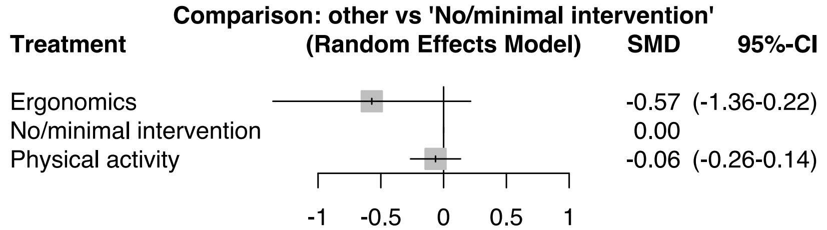

Number of studies: 2, number of pairwise comparisons: 2; SMD: standardised mean difference (negative values are beneficial); CI: confidence interval. Heterogeneity/inconsistency: I^2, Q: not applicable.

g) Outcome intensity of back pain including various regions

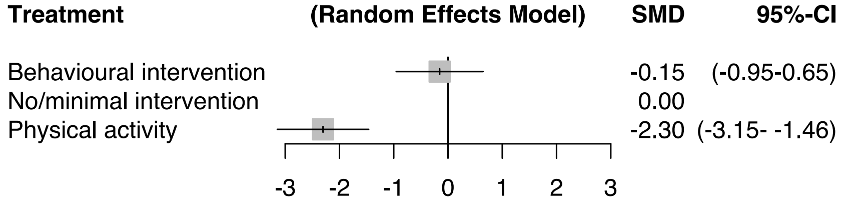

Number of studies: 2, number of pairwise comparisons: 2; SMD: standardised mean difference (negative values are beneficial); CI: confidence interval. Heterogeneity/inconsistency: I^2, Q: not applicable.

Supplementary table S20a-g. P-scores for the additional network meta-analyses for different localisations of back pain

a) Outcome participants with lower back pain

Physical activity	0.9276
Other MC intervention	0.4515
No/minimal intervention	0.3227
Ergonomics	0.2982

b) Outcome participants with neck (-shoulder) pain

Physical activity	0.8356
Education	0.6385
MC intervention with physical activity	0.6055
Other MC intervention	0.5062
No/minimal intervention	0.2300
Ergonomics	0.1842

c) Outcome participants with upper back pain

Physical activity	0.7501
Other MC intervention	0.6650
No/minimal intervention	0.0849

d) Outcome lower back pain intensity

Ergonomics	0.7953
Other MC intervention	0.6539
Physical activity	0.4301
No/minimal intervention	0.1206

e) Outcome neck (-shoulder) pain intensity

Ergonomics	0.8912
MC intervention with physical activity	0.6881
Other MC intervention	0.5021
Physical activity	0.4561
Education	0.3294
No/minimal intervention	0.1331

f) Outcome upper back pain intensity

Ergonomics	0.9062
Physical activity	0.4232
No/minimal intervention	0.1706

g) Outcome intensity of back pain including various regions

Physical activity	0.9999
Behavioural intervention	0.3232
No/minimal intervention	0.1768

Higher scores indicate greater benefit; MC: multicomponent.

Supplementary figure S12a-b. Forest plots for the sensitivity analyses for medium and long term follow-up – active interventions versus no/minimal intervention

a) Medium term follow-up (6- <12 months) – Outcome participants with back pain

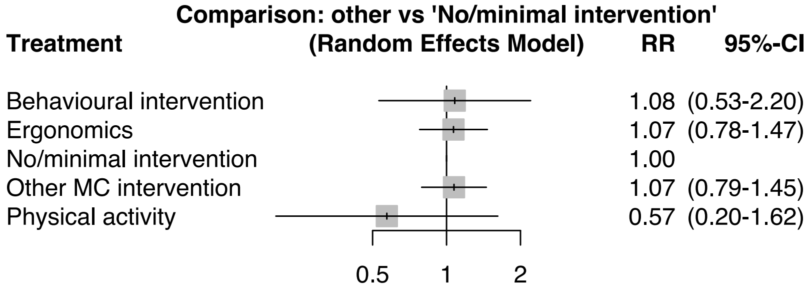

Number of studies: 5, number of pairwise comparisons: 5; RR: risk ratio (RR<1 is beneficial); CI: confidence interval; MC: multicomponent. Heterogeneity/inconsistency: I^2=0%; Q (df=1)=0, p=0.9453.

b) Long term follow-up (≥12 months) – Outcome participants with back pain

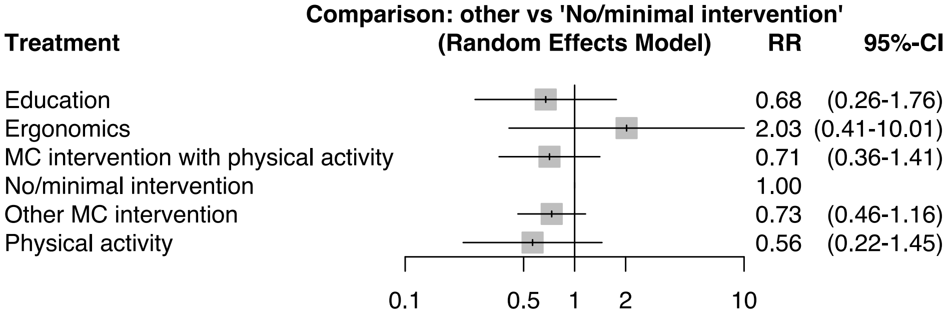

Number of studies: 8, number of pairwise comparisons: 10; RR: risk ratio (RR<1 is beneficial); CI: confidence interval; MC: multicomponent. Heterogeneity/inconsistency: I^2=44.8%; Q (df=4)=7.24, p=0.1236.

Supplementary table S21a-b. P-scores for the sensitivity analyses for medium and long term follow-up

a) Medium term follow-up (6- <12 months) – Outcome participants with back pain

Physical activity	0.8589
No/minimal intervention	0.5151
Behavioural intervention	0.3896
Ergonomics	0.3735
Other MC intervention	0.3630

b) Long term follow-up (≥12 months) – Outcome participants with back pain

Physical activity	0.7827
Education	0.6183
Other MC intervention	0.6036
MC intervention with physical activity	0.5891
No/minimal intervention	0.2783
Ergonomics	0.1279

Higher scores indicate greater benefit; MC: multicomponent.

Supplementary figure S13a-b. Forest plot for the sensitivity analyses for different intervention durations – active interventions versus no/minimal intervention

a) Intervention duration ≤6 months – Outcome participants with back pain

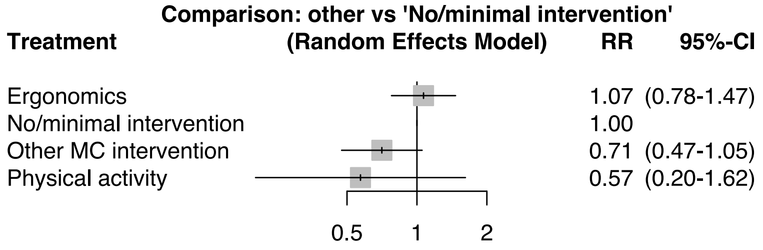

Number of studies: 6, number of pairwise comparisons: 6; RR: risk ratio (RR<1 is beneficial); CI: confidence interval; MC: multicomponent. Heterogeneity/inconsistency: I^2=0%; Q (df=3)=2.66, p=0.4464.

b) Intervention duration >6 months – Outcome participants with back pain

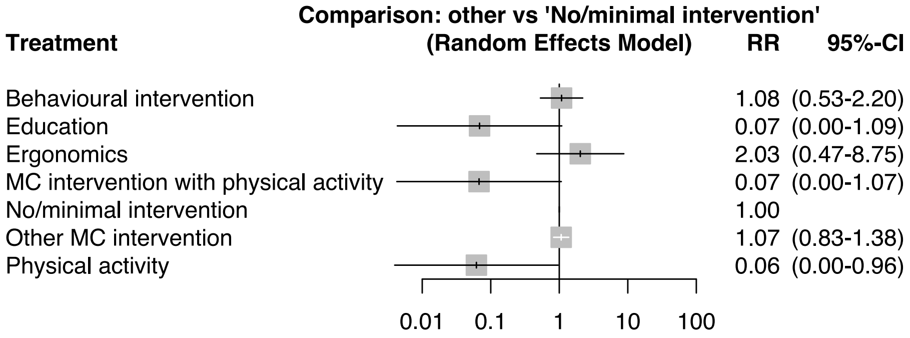

Number of studies: 6, number of pairwise comparisons: 8; RR: risk ratio (RR<1 is beneficial); CI: confidence interval; MC: multicomponent. Heterogeneity/inconsistency: I^2=0%; Q (df=1)=0, p=0.9449.

Supplementary table S22a-b. P-scores for the sensitivity analyses for different intervention durations

a) Intervention duration ≤6 months – Outcome participants with back pain

Physical activity	0.7898
Other MC intervention	0.7516
No/minimal intervention	0.2820
Ergonomics	0.1767

b) Intervention duration >6 months – Outcome participants with back pain

Physical activity	0.8968
MC intervention with physical activity	0.7888
Education	0.7673
No/minimal intervention	0.3635
Behavioural intervention	0.2934
Other MC intervention	0.2832
Ergonomics	0.1071

Higher scores indicate greater benefit; MC: multicomponent.

Supplementary figure S14. Comparison-adjusted funnel plot

Outcome participants with back pain

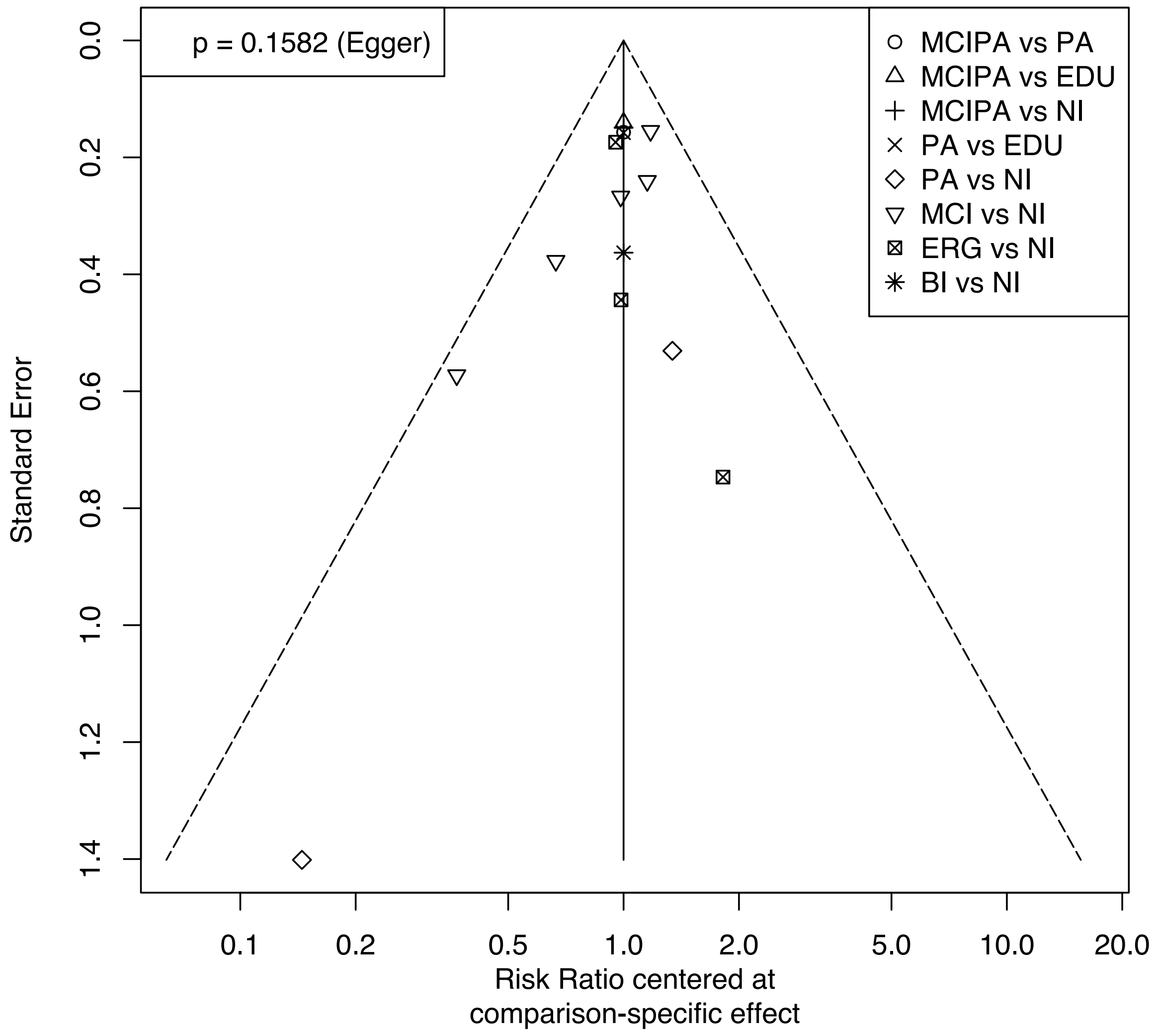

MCIPA: Multicomponent intervention with physical activity; PA: Physical activity; EDU: Education; NI: No/minimal intervention; MCI: Other multicomponent intervention; ERG: Ergonomics; BI: Behavioural intervention.

References

1. Hutton B, Salanti G, Caldwell DM, Chaimani A, Schmid CH, Cameron C, et al. The PRISMA Extension Statement for Reporting of Systematic Reviews Incorporating Network Meta-analyses of Health Care Interventions: Checklist and Explanations. Ann Intern Med. 2015;162(11):777-84.

2. Cohen J. A power primer. Psychological Bulletin. 1992;112(1):155-9.

3. Brakenridge CL, Healy GN, Winkler EA, Fjeldsoe BS. Usage, Acceptability, and Effectiveness of an Activity Tracker in a Randomized Trial of a Workplace Sitting Intervention: Mixed-Methods Evaluation. Interact J Med Res. 2018;7(1):e5.

4. Hadgraft NT, Willenberg L, LaMontagne AD, Malkoski K, Dunstan DW, Healy GN, et al. Reducing occupational sitting: Workers' perspectives on participation in a multi-component intervention. Int J Behav Nutr Phys Act. 2017;14(1):73.

5. Biddle SJH, O'Connell SE, Davies MJ, Dunstan D, Edwardson CL, Esliger DW, et al. Reducing sitting at work: process evaluation of the SMArT Work (Stand More At Work) intervention. Trials. 2020;21(1):403.

6. Welch A, Healy G, Straker L, Comans T, O'Leary S, Melloh M, et al. Process evaluation of a workplace-based health promotion and exercise cluster-randomised trial to increase productivity and reduce neck pain in office workers: a RE-AIM approach. BMC Public Health. 2020;20(1):180.
